# Characterization of Venom Components and Their Phylogenetic Properties in Some Aculeate Bumblebees and Wasps

**DOI:** 10.3390/toxins12010047

**Published:** 2020-01-14

**Authors:** Kyungjae Andrew Yoon, Kyungmun Kim, Woo-Jin Kim, Woo Young Bang, Neung-Ho Ahn, Chang-Hwan Bae, Joo-Hong Yeo, Si Hyeock Lee

**Affiliations:** 1 Research Institute of Agriculture and Life Sciences, Seoul National University, Seoul 08826, Korea; 2 Department of Agricultural Biology, Seoul National University, Seoul 08826, Korea; kkamboosi@snu.ac.kr; 3 EntoCode Co., Seoul 06028, Korea; woojin.kim@snu.ac.kr; 4 National Institute of Biological Resources, Environmental Research Complex, Incheon 22689, Korea; wybang@korea.kr (W.Y.B.); lepi@korea.kr (N.-H.A.); bae0072@korea.kr (C.-H.B.); y1208@korea.kr (J.-H.Y.)

**Keywords:** venom, social wasp, solitary hunting wasp, bumblebee, venom gland, transcriptome analysis

## Abstract

To identify and compare venom components and expression patterns, venom gland-specific transcriptome analyses were conducted for 14 Aculeate bees and wasps. TPM (transcripts per kilobase million) values were normalized using the average transcription level of a reference housekeeping gene (dimethyladenosine transferase). Orthologous venom component genes across the 14 bee and wasp species were identified, and their relative abundance in each species was determined by comparing normalized TPM values. Based on signal sequences in the transcripts, the genes of novel venom components were identified and characterized to encode potential allergens. Most of the allergens and pain-producing factors (arginine kinase, hyaluronidase, mastoparan, phospholipase A1, phospholipase A2, and venom allergen 5) showed extremely high expression levels in social wasps. Acid phosphatase, neprilysin, and tachykinin, which are known allergens and neurotoxic peptides, were found in the venom glands of solitary wasps more often than in social wasps. In the venom glands of bumblebees, few or no transcripts of major allergens or pain-producing factors were identified. Taken together, these results indicate that differential expression patterns of the venom genes in some Aculeate species imply that some wasps and bumblebee species have unique groups of highly expressed venom components. Some venom components reflected the Aculeate species phylogeny, but others did not. This unique evolution of specific venom components in different groups of some wasps and bumblebee species might have been shaped in response to both ecological and behavioral influences.

## 1. Introduction

Wasps are an extremely diverse group of hymenopteran insects that includes an estimated 70,000 species worldwide. Wasps belong to the suborder Apocrita (Hymenoptera), which is divided into Parasitica and Aculeata [1]. Parasitica comprises most of the parasitoid wasps, whereas Aculeata contains parasitoids and predatory wasps, in which the ovipositor is modified to transmit venom rather than being an egg-positioning device [2]. Aculeate wasps are divided into solitary and social groups [3]. 

Most of Aculeate wasps, approximately 95%, are solitary wasps, which build their own nests and store their prey [4]. Most solitary wasps paralyze and preserve prey to provision their young using venom composed of bioactive molecules with various functions, including paralysis, antimicrobial activity, and developmental arrest [5].

All social wasps belong to the family Vespidae, which occupies only a small portion of Aculeata. Social wasps form colonies of various sizes, and their sting is generally reserved for defensive use against intruders [6,7,8]. Some social vespid wasps have guards that are more likely than others in the colony to sting intruders, and old individuals of the species *Polybia occidentalis* are reported to defend their colonies more actively than young individuals [9]. Once attacked or disturbed by intruders, including humans, an attack pheromone can cause fatal mass envenomation by attracting all wasps in the colony [10]. Social wasps capture arthropod prey without stinging and masticate the prey for their brood meal [11]. Because social wasps do not paralyze or preserve prey, their venom appears to have evolved as a defensive tool [5]. Because the venoms of social wasps contain various allergens that can cause an anaphylaxis reaction in humans, their medical and clinical aspects have been studied extensively. Social wasp venoms cause pain, local edema, erythema, cardiovascular disturbances, and anaphylactic shock by increasing the permeability of blood vessels [12]. Many patients exposed to wasp venoms develop systemic reactions and immediate or late reactions such as acute renal failure, hemolysis and coagulopathy, and severe neurological disturbances such as cerebral infarction [13,14,15]. Immunological reactions, such as type I hypersensitivity caused by venom allergens, have also been observed [16]. Those symptoms are caused by biologically active peptides in venom, such as mastoparan, chemotactic peptides and kinins [5,17,18]. Venom allergens include arginine kinase, phospholipase A (A1 and A2), hyaluronidase, cysteine-rich secretory proteins, antigen 5, pathogenesis-related proteins (e.g., CAP), and serine proteases [5].

Bumblebees, which belong to the family Apidae, are increasingly used for pollination of greenhouse plants because of their naive characteristics and lifestyle fit for pollinating. They are not aggressive and do not sting unless disturbed near their nests or intentionally touched [19]. Although the frequency of stinging incidents is expected to increase with increased use of bumblebees for pollination in greenhouses, little information has been available on the comparative toxicity of bumblebee venom.

Venom components and their abundance in bee and wasp venom have mostly been studied in individual species [5,20]. Only a few studies have compared the components in venoms of two closely-related wasp species [21,22]. A comparative analysis of venom gland transcriptomes of two closely-related social hornet species (*Vespa crabro* and *V. analis*) revealed similar overall venom components, but most of the major venom component genes were more abundantly transcribed in *V. crabro* [21]. However, little attempt has been made for the systematic analysis and comparison of the venom components in multiple bee and wasp species. The recent introduction of cost-effective, high-throughput deep-sequencing technologies has enabled rapid identification of genes encoding various venom peptides and proteins from venom gland transcriptomes.

Although the transcriptome profiles of venom gland may not reflect the actual composition profiles of venom proteins and peptides, venom gland transcriptomic approaches have shed some light on the composition and evolution of venom in various venomous animals (reviewed by [23]). The transcriptomic approach is particularly useful when target animals have limited sizes as in case of bees and wasps, and thus venom proteomics are not feasible (reviewed by [23]). In addition, high correlations were also reported between venom gland transcriptomes and venom proteomes in 10 of our 11 comparisons of snake venoms [24].

Therefore, we conducted a comparative transcriptomic characterization of the venom glands of 14 Aculeate bee and wasp species. The TPM (transcripts per kilobase million) values of representative venom components were normalized using the average transcription level of a reference housekeeping gene. The orthologous venom component genes were identified across the 14 bee and wasp species, and their relative abundance was compared across species. Based on signal sequences, genes for putative novel venom components were identified and characterized. Finally, we investigated the evolutionary aspects of the venom components in accordance with ecological and behavioral features. This systematic and comparative study of the venom gland transcriptomes of different bee and wasp species provides new insights into the evolution and phylogeny of venom. 

## 2. Results and Discussion

### 2.1. Differential Transcriptional Profiles of the Venom Glands of Some Aculeate Species

Total reads of 5.89, 6.39, 7.13, 5.74, 6.22, 6.16, 11.3, 11.4, 8.2, 8.07, 8.1, 7.66, 10.9, and 10.6 Gb were obtained from RNA sequencing of the venom glands of *Eumenes decoratus*, *Sphecidae* sp., *Anterhynchium flavomarginatum*, *Sceliphron deforme*, *V. crabro*, *V. analis*, *V. dybowskii*, *V. simillima*, *Parapolybia varia*, *Polistes snelleni*, *Polistes rothneyi*, *Bombus ardens*, *B. consobrinus*, and *B. ussurensis*, respectively. De novo assembly of the trimmed sequence data resulted in 18,062, 31,134, 21,512, 24,483, 11,097, 12,531, 21,227, 29,150, 20,378, 27,742, 25,436, 31,366, 21,625, and 26,425 assembled contigs, respectively. A total of 16,357, 27,423, 19,033, 20,097, 10,321, 11,284, 19,627, 27,065, 18,539, 25,879, 23,475, 26,158, 20,050, and 22,619 genes, respectively, were identified by BLAST search (Table 1).

To verify the transcriptional abundance of major venomic genes in Aculeate bees and wasps, 10 venomic gens of two social wasps (*P. rothneyi* and *P. snelleni*), one solitary hunting wasp (*E. decoratus*) and one bumblebee (*B. ardens*) were investigated by conducting quantitative real-time PCR (qPCR) and compared with TPM value of their transcriptome data (Figure S1). Relative transcription levels showed significantly similar expression pattern with that of TPM values indicating that relative transcriptional abundances of major venomic genes estimated from TPM values are reliable.

#### 2.1.1. Venom Proteins Highly Expressed in Solitary Hunting Wasps

**Figure 1:**
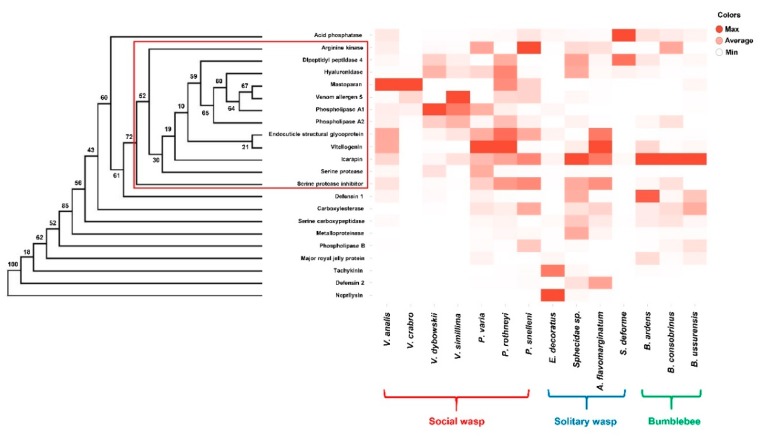
Hierarchial clustering of the 22 major orthologous venom genes in the 14 Aculeate bumblebee and wasp species (left panel), and their differential expression profiles represented in a heat map (right panel). In the heat map, each row represents the venom gene analyzed, and each column represents the species tested. The percentages of bootstrap values based on 1000 pseudoreplicates are shown on each node of the dendrogram. Red rectangle indicates the clade clustered with most of the allergens and pain-producing factors, which showed significantly high expression levels in the social wasps.

Some venom component genes, such as acid phosphatase, neprilysin, and tachykinin, were abundantly expressed in solitary hunting wasps and expression levels of allergens and pain-producing factors were low in social wasps and bumblebees. Acid phosphatase was found with a high frequency in the venom glands of solitary wasps (Figure 1). In particular, the solitary hunting wasp *S. deforme* exhibited an extremely high expression level of acid phosphatase (TPM = 83,708). The overall TPM values for acid phosphatase in other wasps and bumblebee species were less than 100 (except that of *V. analis*), suggesting that acid phosphatase might have a particularly significant role in the venom of solitary hunting wasps (Figure 1). Acid phosphatase in the venom of the endoparasitoid wasp *Pteromalus puparum* was suggested to affect the host’s immune response and physiology [25]. However, that of the endoparasitoid wasp *Pimpla hypochondriaca* plays no known role in hemocyte immunology [26]. 

Neprilysin showed an extremely high expression level (TPM = 7387) in *E. decoratus* and low expression levels (TPM < 50) in two solitary hunting wasps, *Sphecidae* sp. and *A. flavomarginatum* (Figure 1). The social wasps showed none of the transcript for neprilysin, indicating that neprilysin might be specifically expressed in the venom glands of solitary hunting wasps. Neprilysin is involved in metabolism of regulatory peptides in mammalian nervous systems, and it is known to inactivate peptide transmitters and their modulators by terminating brain neuropeptides at peptidergic synapses [27,28]. This implies that paralysis might be one of the major functions of the venom of solitary hunting wasps.

Tachykinin was highly expressed in the solitary hunting wasp *E. decoratus* (TPM = 4241), and the other wasps and bumblebee species tested showed TPM values lower than 40 (Figure 1). Tachykinin is a neurotoxic peptide in wasp venom that is known to induce hypokinesia, a “zombie-like” behavioral state caused by direct envenomation into the central nervous system. Synthetic AcVTK mimics the venom tachykinins of the parasitoid jewel wasp *Ampulex compressa*. Injection of synthetic AcVTK into the subesophageal ganglion of the American cockroach (the jewel wasp host) produced behavioral changes, including suppression of the escape response and reduced spontaneous walking [29]. Thus, solitary wasps might use tachykinin to change a prey’s physiology and behavior, ensuring availability of fresh prey for their larvae. 

Defensin 2 was abundantly identified in the venom glands of solitary hunting wasps (*E. decoratus*, *Sphecidae* sp., and *A. flavomarginatum*), with only slight expression in some social wasps (*Parapolybia varia* and *Polistes rothneyi*). This finding implies that defensin 2 in solitary hunting wasps might be used to preserve prey.

#### 2.1.2. Venom Proteins Highly Expressed in Social Wasps

According to the hierarchical clustering analysis for the 22 representative venom genes, most of the allergens and pain-producing factors (arginine kinase, dipeptidyl peptidase 4, endocuticle structural glycoprotein, hyaluronidase, mastoparan, phospholipase A1, phospholipase A2, serine protease, serine protease inhibitor, venom allergen 5, and vitellogenin) were clustered in a clade although its bootstrap value (52%) did not strongly support the topology (Figure 1; see red rectangle). These venom genes showed significantly high expression levels in the social wasps examined. This suggests that social wasps might have evolved to use these components as defensive tools against intruding species (Figure 1). 

Arginine kinase, which is known to cause an allergic reaction in vertebrates [30], was identified in the venom glands of most Aculeate wasps and bumblebee species, suggesting its ancient origins. The arginine kinase of *P. varia* exhibited extremely high TPM value among the 14 species tested, and most of the social wasps showed values above 300 (except *V. crabro*, *V. simillima* and *P. rothneyi*). Solitary hunting wasps and bumblebees (except *B. ardens* and *B. ussurensis*) also showed considerable levels of arginine kinase expression, and expression levels were low in social wasps (i.e., TPM values < 100). *B. consobrinus* showed a high expression level of arginine kinase (TPM = 832) (Figure 1). Presence of arginine kinase in both wasps and bumblebees suggests that it has likely been used as an allergen in the lineages of both bees and wasps.

Most solitary and social wasps showed high expression levels of dipeptidyl peptidase 4, with particularly high expression in *S. deforme* (TPM = 54,119) (Figure 1). Dipeptidyl peptidase 4 is reported to be a major allergen in the social wasp *Polistes dominula*, with more than 60% of Polistes venom sensitized—patients showing IgE-reactivity [31]. Its relatively low expression levels in bumblebees suggest that dipeptidyl peptidase 4 may function as a wasp-specific allergen.

Endocuticle structural glycoprotein, which is known to cause allergic reactions, was highly expressed in most social wasps except *V. crabro* (Figure 1). The carbohydrate moiety of insect glycoproteins is reported to play a pivotal role in human IgE binding [32], implying that endocuticle structural glycoprotein may contribute to allergic reactions to social wasp venoms.

Most social wasps, *V. crabro* and *V. dybowskii* in particular, showed high expression levels of hyaluronidase (TPM values > 1000), although some of the solitary hunting wasps (*S. deforme* and *Sphecidae* sp.) also exhibited a moderate level of expression (TPM = 893 and 556) (Figure 1). This result indicates that hyaluronidase, which is a venom spreading factor, might play a major role in the venom of both social and solitary wasps [5].

Mastoparan was identified in the venom of most social wasps (*V. crabro*, *V. analis*, *P. snelleni*, and *P. rothneyi*), some solitary hunting wasps (*A. flavomarginatum* and *S. deforme*), and a bumblebee species (*B. ussurensis*). *V. crabro* mastoparan showed an extremely high TPM value among all venom genes in that species. Mastoparan causes allergic inflammation and mast cell degranulation in vertebrates [21]. Therefore, social wasp venoms containing relatively high levels of mastoparan are likely to be highly allergenic to vertebrates (Figure 1). Identification of mastoparan in both wasp and bumblebee species suggests its ancient origin. 

Phospholipase A1 was identified in the venom glands of all social wasps and some of the solitary hunting wasps and bumblebees (Figure 1). Most social wasps exhibited extremely high expression of phospholipase A1 (with the exception of *P. snelleni* and *P. rothneyi*). Phospholipase A2 was also highly expressed in most social wasps, exhibiting TPM value > 600, especially in *V. crabro*, *V. dybowskii,* and *V. simillima* (Figure 1). As phospholipase A1 and A2 are major Hymenopteran venom allergens in humans [33,34], their high expression in the venoms social wasps suggests that they are likely to be involved in the defensive function of venom.

Abundant transcripts of serine protease were found in the social wasps, among which *V. dybowskii* showed the highest expression (TPM = 1916). Few or no transcripts of serine protease were expressed in solitary wasps and bumblebees (Figure 1). Serine protease is an important allergen with significant IgE binding activity [35,36,37], which indicates that it might have defensive role to social wasps.

Most of the Aculeate wasps and bumblebee species tested showed abundant transcripts of serine protease inhibitor (Serpin), except *V. crabro*, *V. simillima*, *E. decorates,* and *S. deforme*. Social wasps such as *V. analis*, *P. varia,* and *P. rothneyi* showed moderate expression of serpin (TPM = 736, 660, and 604, respectively) (Figure 1). Serpin is known to have IgE-sensitizing potential in honeybee venom-allergic patients, indicating its potency as an allergen [38]. 

The social wasps *V. crabro* and *V. simillima* exhibited extremely high expression of venom allergen 5, which is a major allergen identified in many insect venoms [39]. This finding suggests that the venom of social wasps contains abundant allergens (Figure 1).

Vitellogenin is a newly found honeybee venom allergen that shows IgE-sensitizing reactivity in venom-sensitized patients [40]. *V. analis*, *P. varia*, *P. rothneyi,* and *A. flavomarginatum* exhibited extremely high expression of vitellogenin, and *V. dybowskii,* and *B. ardens* showed considerable expression (Figure 1). Thus, social wasp venoms with high expression of vitellogenin might possess high allergenicity.

#### 2.1.3. Venom Proteins Highly Expressed in Bumblebees

In the venom glands of bumblebees, few transcripts for major allergens or pain-producing factors were found, implying that bumblebee venoms are likely to have low toxicity (Figure 1). Interestingly, defensin 2 was not detected in the venom glands of the three bumblebees tested, but defensin 1 (an antimicrobial peptide) showed extremely high expression in *B. ardens* and *B. ussurensis*. 

Bumblebees (except *B. consobrinus*) had high expression level of defensin 1. The social wasps *V. analis*, *V. dybowskii,* and *P. varia* showed TPM values > 200; other wasps (except *Sphecidae* sp.) exhibited little to no defensin 1 transcript expression (Figure 1). The high bumblebee-specific expression of defensin 1, which is well known as an antimicrobial peptide, suggests that bumblebees may have exploited defensin to sanitize their nests, which are built underground. The small number of transcripts for defensin 1 in *B. consobrinus* suggest that this species might be more susceptible to infection than the other bumblebees examined in this study. 

#### 2.1.4. Expression Patterns of Other Venom Proteins

Carboxylesterase 6 causes allergic reactions in humans and is also known as Api m 8, which is a honeybee venom allergen [41]. The social wasp *P. varia* showed the highest expression of carboxylesterase 6, and the bumblebees *B. consobrinus* and *B. ussurensis* exhibited TPM values higher than 200 (Figure 1). Solitary wasps had few or no carboxylesterase 6 transcripts, suggesting that this protein might have a defensive role in social wasp and bumblebee venoms.

Bumblebees, especially *B. consobrinus,* showed a high expression level of icarapin (TPM = 8901), but all solitary (except for *E. decoratus*) and social wasps also exhibited high TPM value (Figure 1). Icarapin is a glycoprotein allergen with low abundance in honeybee venom [42] that nonetheless shows greater than 50% IgE-reactivity in honeybee venom-sensitized patients [43]. The extremely high expression of icarapin in bumblebees and its considerable expression in most social and solitary hunting wasps indicate that some Aculeate wasps and bumblebee species are likely to commonly use icarapin for defense by causing allergic reactions.

Metalloproteinase generally showed low expression in the Aculeate wasps and bumblebee species tested, except the solitary hunting wasp *Sphecidae* sp., which exhibited a considerable number of transcripts (TPM = 516) (Figure 1). Metalloproteinase is a toxin found abundantly in snake venom and causes hemorrhaging and interferes with the hemostatic system [44]. Metalloproteinase is also known to play a role in tumor invasion and metastasis by degrading extracellular matrix [45]. The generally low abundance of metalloproteinase in the venoms of Aculeate species may indicate its minor role for wasps and bumblebee species.

Phospholipase B showed a low abundance in most Aculeate wasps and bumblebee species examined (Figure 1). Although phospholipase B was reported to have cytotoxicity in snake venom [46], its low expression may imply its minor role as a venom component of Aculeate species, especially compared with phospholipase A1 and A2.

Serine carboxypeptidase is an exopeptidase that is a major allergen in honeybee venom [47]. It is known to participate in protein digestion in the guts of animals [48] and is found among the yolk proteins in mosquito oocytes [49]. Serine carboxypeptidase showed low abundance in solitary wasps and bumblebees (except *B. consobrinus*, TPM = 161) (Figure 1). The presence of few or no transcripts in social wasps indicates that serine carboxypeptidase might have a minor function in the venom of some Aculeate wasps and bumblebee species.

### 2.2. Identification of Putatively Novel Venom Components in Some Aculeate Bees and Wasps

To identify the properties of novel venom genes, we conducted signal peptide prediction with the top 100 highly expressed genes (Tables S1–S14) and summarized the resulting secretory venom components in Table 2. A total of 15 genes coding secretory proteins were identified in all 14 Aculeate bee/wasp species. Among them, nine genes were found to encode known venom proteins: defensin 1, endocuticle structural glycoprotein, hyaluronidase, icarapin, neprilysin, phospholipase A1 and A2, serine protease, and venom allergen 5 (Table 2). One of the reasons that not all known venom component genes were identified by signal peptide prediction is that many of the venom component genes lack the 5’ end transcript sequences where the signal peptide sequences are located, probably due to incomplete reverse transcription of the 5’end mRNA by reverse transcriptase [50] or incomplete reconstruction of the full-length transcripts from short cDNA reads [51]. 

Nevertheless, signal peptide prediction enabled identification of six genes encoding uncharacterized secretory proteins (USPs; USP1–USP6) that were commonly expressed in all examined social wasps, bumblebees, and one solitary hunting wasp, *A. flavomarginatum* (Table 2). Such common and high expression pattern in most Aculeate wasps and bumblebee species examined suggests that the components are likely putative constituents of venom.

#### Properties of the Six USPs (USP1–USP6) as Putative Allergenic Proteins

USP1 exhibited high expression in most social wasps, especially in *V. simillima* (TPM = 29,700) (Table 2). The USP1 gene contained 106 to 112 amino acid sequences and was identified as a putative pilosulin from the Australian ant *Myrmecia banksi* (56.3%, accession number: BAD36780), implying its allergenic function in social wasp venom (Figure S2).

USP2 exhibited high expression in two social wasps, *V. analis* and *P. varia* (TPM > 2600), and a considerable expression level in the solitary hunting wasp *A. flavomarginatum* (TPM = 865). USP2 *P. varia* showed 70.3% sequence similarity with putative tropomyosin from the Pacific oyster *Crassostrea gigas* (BAH10152), suggesting USP2 as a putative allergen (Figure S3).

USP3 was highly expressed in two bumblebees, *B. consobrinus* and *B. ussurensis* (TPM = 1805 and 1537, respectively). USP3 encodes 167 to 173 amino acid sequences that were identified as related to the putative IgE-binding protein MnSOD of the rubber tree *Hevea brasiliensis* (CAC13961), with 59.6% sequence similarity (Figure S4). 

USP4 was identified in the venom gland of the bumblebees *B. ardens* and *B. ussurensis* and had high expression (TPM = 1800 and 1741, respectively). In these two bumblebees, USP4 encoded the same 140-amino acid sequence and exhibited 58.8% sequence similarity with the allergen alpha amylase inhibitor-like protein from common wheat, *Triticum aestivum* (CAA35597) (Figure S5). Thus, USP3 and USP4 in bumblebees might have the potential to induce allergic sensitization.

USP5 was identified in the social wasp *P. varia* and the solitary hunting wasp *A. flavomarginatum* with high expression (TPM = 2439 and 1322, respectively). Only USP5 in *A. flavomarginatum,* which encodes a 143-amino acid sequence, was identified as the putative venom allergen Sol g 4.01 precursor from the tropical fire ant *Solenopsis geminata* (AF230383), with 54.6% sequence similarity (Figure S6).

USP6 showed high expression in the social wasp *V. dybowskii* and the solitary hunting wasp *A. flavomarginatum* (TPM = 1584 and 1944, respectively). USP6, composed of 129 to 130-amino acid sequences in two species, could not be identified in a BLAST search or an allergen database search (Figure S7).

### 2.3. Evolutionary Patterns in the Venom of Some Aculeate Bees and Wasps

Aculeata belongs to Apocrita, Hymenoptera and is divided into the three superfamilies Chrysidoidea, Apoidea, and Vespoidea [5]. Bumblebees belong to the Apidae family, and *Sphecidae* sp. And *S. deforme* belong to the Sphecidae family, which is divided from the superfamily Apoidea. The Vespa species (*V. analis*, *V. crabro*, *V. dybowskii,* and *V. simillima*) belong to the subfamily Vespinae, and the Polistes species (*P. rothneyi* and *P. snelleni*) and *P. varia* belong to the subfamily Polistinae, which is grouped with Vespinae. *E. decoratus* and *A. flavomarginatum* belong to the subfamily Eumeninae [5].

In the phylogenetic tree of venom components in some Aculeate bees and wasps, eight venom proteins (carboxylesterase 6, dipeptidyl peptidase 4, endocuticle structural glycoprotein, icarapin, major royal jelly protein, phospholipase B, serine protease inhibitor and vitellogenin) matched the evolutionary pattern of Aculeate species (Figures S8–S15). In phylogenetic analysis, icarapin of social wasps and that of bumblebees were clearly separated into two branches. *Sphecidae* sp. and *S. deforme* were grouped and closely related to the bumblebee branch, whereas *E. decoratus* and *A. flavomarginatum* were grouped and showed a close relationship with the social wasp branch (Figure S11). Thus, icarapin appears to reflect the phylogenetic relationships among some Aculeate bees and wasps.

However, the number of venom component proteins that do not phylogenetically reflect the Aculeate lineage was larger than the number that do reflect. The results from the phylogenetic analyses of acid phosphatase, defensin 2, hyaluronidase, mastoparan, metalloproteinase, neprilysin, phospholipase A1, serine carboxypeptidase and serine protease revealed that one species was positioned as an out group or grouped with another branch in a way not coincident with the evolutionary pattern of Aculeate species (Figures S16–S24). For example, *S. deforme* was clustered as an out group in acid phosphatase (Figure S16), and *Sphecidae* sp. was grouped with Formicidae in the phylogenetic tree of defensin 2 (Figure S17). Thus, these venom proteins only appear to semi-reflect the phylogenetic pattern of Aculeate species, and in the cases when venom proteins exhibiting an isolated phylogeny, they likely have evolved to have unique functions. In the phylogenetic trees of arginine kinase, defensin 1, phospholipase A2, tachykinin, and venom allergen 5, two to five species were grouped with other branches or positioned independently (Figures S25–S29). An amino acid sequence alignment showed a high degree of sequence conservancy, but the phylogeny of venom allergen 5 did not clearly match the species phylogeny (Figure S29). Two solitary hunting wasps that belong to different clades in the Aculeate phylogeny were grouped together. Likewise, social wasps and *B. ussurensis* were grouped together. Thus, venom allergen 5 is a major allergen in the venom of social wasps, but it can be speculated that its evolutionary history is different across different Hymenopteran species. However, as only a limited number of wasps and bumblebee species were compared in this study, a larger scale observation should be conducted to confirm this notion. 

The unique evolutionary patterns of these venom proteins or allergens might be caused by specific ecological behaviors. Adaptive traits of the venom could provide a selective advantage in some species, so they exhibit differential evolutionary patterns through the phylogenetic trees. To confirm this idea, however, a robust phylogenetic comparative analysis with larger numbers of species should be conducted. Further studies to identify unique traits, such as ecological behavior or function of the venom, would provide a clearer understanding of venom evolution.

We conducted venom gland—specific transcriptome analyses in 14 Aculeate bees and wasps and found novel venomic genes that show conserved domains with annotated major allergens. These novel genes are potent allergens to be verified and could provide fundamental data for venom immunotherapy (VIT). VIT is accepted as a safe and effective treatment. However, due to genetic differences in Hymenoptera species, side effects can be frequent, and success rates vary [41,52]. To improve the safety and efficacy of Hymenoptera VIT, use of recombinant venom allergens that have domains highly conserved in bees and wasps could provide a solution for systemic allergic side effects. The venom proteins in some Aculeate bees and wasps possessed average amino acid sequence similarities of 38% to 96%. Arginine kinase showed the highest percentage similarity among all venom proteins, exhibiting 92–100% sequence similarity. Because arginine kinase showed considerable expression in the venom glands of solitary and social wasps and bumblebees (Figure 1), use of an epitope region in conserved domains and a recombinant protein could improve the safety and quality of diagnosis approaches and Hymenoptera VIT.

## 3. Conclusions

Acid phosphatase, neprilysin, and tachykinin, which possess neurotoxic activity, were most highly expressed in solitary hunting wasps, suggesting that they use their venom to paralyze and preserve prey to ensure fresh provisions for their progeny. Most major venom allergens and pain-producing factors (arginine kinase, dipeptidyl peptidase 4, endocuticle structural glycoprotein, hyaluronidase, mastoparan, phospholipase A1, phospholipase A2, serine protease, serine protease inhibitor, venom allergen 5, and vitellogenin) exhibited high expression in social wasps, suggesting that social wasps may use venom as a defensive tool against intruding species and possesses high toxicity. Bumblebees showed few transcripts for allergens or pain-producing factors but the highest expression of an antimicrobial peptide, defensin 1, suggesting that bumblebees possess venom with low toxicity, and that they use their venom for sanitizing purposes. Taken together, these results indicate that some Aculeate bees and wasps tested exhibit distinct venom properties and differential venomic gene expression patterns depending on species and sociality. Some venom components (carboxylesterase 6, dipeptidyl peptidase 4, hyaluronidase, icarapin, phospholipase B, serine carboxypeptidase, serine protease inhibitor, and vitellogenin) reflected the Aculeate species phylogeny, but others (acid phosphatase, arginine kinase, defensin 1, defensin 2, major royal jelly protein, mastoparan, metalloproteinase, phospholipase A1, phospholipase A2, serine protease, tachykinin, and venom allergen 5) did not. It can be speculated that this unique evolution of some venom components might have been shaped by specific ecological behaviors. To confirm this notion, however, a phylogenetic comparative analysis with larger numbers of species should be conducted. To the best of our knowledge, this is the first systematic study of venom gland transcriptomic data to compare major venom components across various species of some Aculeate bumblebees and wasps. A broader scale of comparison for venom components (including minor components across diverse bee and wasp species) would elucidate how venoms evolved according to ecological and behavioral pressures.

## 4. Materials and Methods

### 4.1. Wasp and Bumblebee Collection and Total RNA Purification

Female solitary hunting wasps (*E. decoratus*, *Sphecidae* sp., *A. flavomarginatum* and *S. deforme*), social wasps (*V. analis*, *V. crabro*, *V. dybowskii*, *V. simillima*, *P. varia*, *P. snelleni*, and *P. rothneyi*), and bumblebees (*B. ardens*, *B. consobrinus*, and *B. ussurensis*) were collected from several southern regions in Korea. Wasps and bumblebee species were collected and placed in 50 mL tube for 2 h at room temperature to unify the stress conditions of collected specimens. Wasps and three bumblebee species were then anesthetized using low-pressure carbon dioxide. Venom glands were only dissected from the venom apparatus, and total RNA was extracted from 20 dissected venom glands using 200 μL of TRI reagent (Molecular Research Center, Cincinnati, OH, USA) according to the manufacturer’s protocol. As the main goal of this study was to overview the overall expression patterns of putative venom genes across several bumblebees and wasp species, no statistical treatment based on multiple transcriptome analyses were made. Instead, we used the same conditions of venom gland dissection and total RNA extraction for all bumblebee and wasp species to minimize the temporal expression bias between different species.

### 4.2. Construction of an in Silico cDNA Library and Computational Analysis

RNA purity and total RNA integrity were evaluated using a NanoDrop 8000 spectrophotometer (Thermo Fisher Scientific, Waltham MA, USA) and Agilent Technologies 2100 Bioanalyzer (Agilent Technologies, Santa Clara, CA, USA). De novo assembly of Illumina reads was performed based on a previously reported method [53]. The raw reads from RNA-Seq were filtered using the FASTP program [54] to remove low-quality reads, which were base called with an error rate higher than 0.1% (Q-score < 30). The quality-filtered clean reads were subjected to the Trinity de novo assembler v2.8.4 program (GitHub, San Francisco, CA, USA) [51] to construct contigs. To streamline the contigs, sequences with high homology were clustered by the CD-HIT-EST program [55]. Subsequently, the clustered sequences with protein coding capability were selected by the standalone version of the TransDecoder v5.5 program, which was introduced with Trinity [51], to obtain the final version of the in silico cDNA library. The cDNA sequences were annotated by the BLASTX program with the GenBank reference peptide nr database. The Illumina short reads were mapped to the cDNA sequences using the Kallisto program [56] to calculate the expression rate in transcripts per kilobase million (TPM). To identify genes encoding secreted venom components, the top 100 highly expressed genes with signal peptide sequences of 14 Aculeate bees and wasps (*E. decoratus*, *Sphecidae* sp., *A. flavomarginatum*, *S. deforme*, *V. dybowskii*, *V. simillima*, *P. varia*, *P. snelleni*, *P. rothneyi*, *B. ardens*, *B. consobrinus*, and *B. ussurensis*) were predicted using the Phobius program [57] and SignalP-5.0 server (www.cbs.dtu.dk/services/SignalP/). For the USPs, an allergen database search (www.allergenonline.org) was conducted to predict potential allergenicity. The raw data of transcriptome sequencing from following species have been uploaded to NCBI’s Short Read Archive; *E*. *decoratus* (SRR10286816), *Sphecidae* sp. (SRR10286815), *A*. *flavomarginatum* (SRR10286814), *S*. *deforme* (SRR10286825), *V. crabro* (SRR10286826), *V. analis* (SRR10286827), *V. dybowskii* (SRR10286821), *V. simillima* (SRR10286820), *P*. *varia* (SRR10286819), *P*. *snelleni* (SRR10286817), *P*. *rothneyi* (SRR10286818), *B*. *ardens* (SRR10286824), *B. consobrinus* (SRR10286823), and *B. ussurensis* (SRR10286822).

### 4.3. Ortholog Analysis of Venom Component Genes 

We used the OrthoMCL program v2.0.9 [58] with the Markov Cluster algorithm to identify the orthologous paralog group of venom component genes across wasp and bumblebee species. To do this, we used the BLAST v2.2.28+ program (NCBI, Bethesda, MD, USA) before self-alignment of all proteins, with an e-value cutoff of 1e^−5^ and a minimum alignment length of 50 bp. False-positives were removed by filtering at least 50% of the query cover. Domain analysis was performed with the HMMER v3.1b2 [59] program using the Pfam [60] (http://pfam.sanger.ac.uk) database to identify additional functions of each protein.

A reference gene (dimethyladenosine transferase) [61] was selected for verification of transcriptional abundance, and total reads were divided and normalized using the average TPM values for the reference gene in the 14 Aculeate bees and wasps (Table S15). Hierachial clustering analysis for the 22 orthologous venom genes was conducted with UPGMA (unweighted-pair group method with arithmetic mean) clustering method and the genetic distance was calculated by Nei’s D*_A_* distance measurement [62] using POPTREE2 software. The percentages of bootstrap values based on 1000 replicates are shown on each node. Heat map was generated using the heat map function in TIBCO Spotfire^®^ software (TIBCO Software Inc., Palo Alto, CA, USA).

### 4.4. Construction of a Phylogenetic Tree

To investigate structural differences in venom proteins, we aligned the deduced amino acid sequences obtained from the transcriptome data with full-length sequences using CLC Main Workbench 7 (CLC Bio, Waltham, MA, USA). Based on amino acid alignment of the orthologous venom genes, we constructed a phylogenetic tree using maximum likelihood method based on the JTT matrix-based model [63] with 1000 bootstrap replications. The percentages of trees in which the associated taxa clustered together in the bootstrap test (1000 replicates) were shown on each node. The tree was drawn to scale, with branch lengths measured in the number of substitutions per site. Phylogenetic analyses were conducted in MEGA X (Pennsylvania State University, PA, USA). Acid phosphatase (AF321918), defensin 1 (AAA52303), defensin 2 (AAC69554), hyaluronidase (AAC70915), metalloproteinase (AAA58658), phospholipase A2 (AAF09020), and serine protease (CCA61110) of *Homo sapiens* were used as outgroups. The following were also used as outgroups: arginine kinase (JAV48307), phospholipase A1 (JAV47955), and phospholipase B (JAV47950) of *Hadrurus spadix*; mastoparan of an uncultured bacterium (AP032449) [64]; venom allergen 5 (XP_022659921) of *Varroa destructor*; carboxylesterase 6 (KK119399) of *Stegodyphus mimosarum*; icarapin (FX985504) of *Odontomachus monticola*; dipeptidyl peptidase 4 (XP_021001451) and major royal jelly protein (XP_021000670) of *Parasteatoda tepidariorum*; and endocuticle structural glycoprotein (PSN39565), neprilysin (PSN37766), serine carboxypeptidase (PSN48057), serine protease inhibitor (PSN55974), tachykinin (PSN47497), and vitellogenin (CAA06379) of *Blattella germanica*. Major venomic genes of other Hymenopterans, Dipterans, Hemipterans, Lepidopterans and Dictyopterans were included and used as outgroups. Any robust phylogenetic comparative analysis was not feasible due to the limited number of Aculeate wasps and bumblebee species tested in this study.

### 4.5. Quantitative Real-Time PCR

DNase I (Takara, Kyoto, Japan) was used to remove DNA contamination of total RNA of two social wasps (*P. rothneyi* and *P. snelleni*), one solitary hunting wasp (*E. decoratus*) and one bumblebee (*B. ardens*). cDNA was synthesized using SuperScriptIV reverse transcriptase (Invitrogen, Carlsbad, CA, USA). Total 10 venomic genes (arginine kinase, defensin 1, dipeptidyl peptidase 4, hyaluronidase, icarapin, phospholipase A2, serine protease inhibitor, tachykinin, vitellogenin, and neprilysin) and a reference housekeeping gene (dimethyladenosine transferase) were selected for verification of transcriptional abundance. Venomic gene specific primers were designed to have similar lengths and %GC content (Table S16) and qPCR was conducted using a previously described protocol [53].

## Figures and Tables

**Table 1:** Summary of the venom gland transcriptome cDNA libraries of 14 Aculeate bees/wasps.

Species	Total Number of Reads	Total Base Pairs	Trimmed Reads	Total Number of Assembled Transcripts	Total Number of Annotated Transcripts
*E. decorates*	58,988,410	5,957,829,410	55,681,360	18,062	16,357
*Sphecidae* sp.	63,954,192	6,459,373,392	60,641,236	31,134	27,423
*A. flavomarginatum*	71,380,878	7,209,468,678	67,788,990	21,512	19,033
*S. deforme*	57,404,468	5,797,851,268	52,911,920	24,483	20,097
*V. crabro*	62,246,264	5,756,774,997	56,318,830	11,097	10,321
*V. analis*	61,648,078	5,686,981,519	55,419,968	12,531	11,284
*V. dybowskii*	113,343,134	11,447,656,534	103,185,332	21,227	19,627
*V. simillima*	114,592,946	11,573,887,546	89,279,802	29,150	27,065
*P. varia*	82,025,386	8,284,563,986	75,702,936	20,378	18,539
*P. snelleni*	80,798,216	8,160,649,816	75,374,540	27,742	25,879
*P. rothneyi*	81,060,754	8,187,136,154	76,096,056	25,436	23,475
*B. ardens*	76,622,404	7,738,862,804	71,427,168	31,366	26,158
*B. consobrinus*	109,962,550	11,106,217,550	72,432,174	21,625	20,050
*B. ussurensis*	106,973,794	10,804,353,194	99,138,794	26,425	22,619

**Table 2:** Expression levels of major secretory venom components in 14 Aculeate bees/wasps.

Description	TPM Value of Top 100 Highly Expressed Genes ^a^													
	V. an	V. cr	V. dy	V. si	P. va	P. ro	P. sn	E. de	Sph	A. fl	S. de	B. ar	B. co	B. us
Defensin 1			-	-	1652	-	-	-	-	-	-	1163	-	-
Endocuticle structural glycoprotein	6209		-	1132	4404	3779	-	-	4847	-	-	-	-	-
Hyaluronidase		10,229	4566	1409	-	1275	-	8511	-	-	-	-	-	-
Icarapin	1495		1167	725	-	-	-	-	2857	2081	-	2039	4623	6943
Neprilysin			-	-	-	-	-	4174	-	-	1895	-	-	-
Phospholipase A1		49,149	106,395	29,423	6440	1246	-	-	-	-	-	-	-	-
Phospholipase A2		4722	1547	952	-	-	-	-	-	-	-	-	-	1157
Serine protease			1301	-	1737	947	-	22,355	-	-	-	-	-	-
Venom allergen 5		150,567	65,462	32,241	13,212	-	-	-	-	-	-	-	-	-
Uncharacterized protein 1		2677	-	29,700	4756	-	874	-	-	-	-	-	6955	-
Uncharacterized protein 2	17,769		1199	-	2611	-	-	-	-	865	-	-	-	-
Uncharacterized protein 3			2125	-	-	-	-	-	-	-	-	-	1805	1537
Uncharacterized protein 4			-	-	-	-	-	-	-	-	-	1800	-	1741
Uncharacterized protein 5			1584	-	-	-	-	-	-	1944	-	-	-	-
Uncharacterized protein 6			-	-	2439	-	-	-	-	1322	-	-	-	-

^a^ Wasp species abbreviations: V. an, *Vespa analis*; V. cr, *Vespa crabro*; V. dy, *Vespa dybowskii*; V. si, *Vespa simillima*; P. va, *Parapolybia varia*; P. ro, *Polistes rothneyi*; P. sn, *Polistes snelleni*; E. de, *Eumenes decoratus*; Sph, *Sphecidae* sp.; A. fl, *Anterhynchium flavomarginatum*; S. de, *Sceliphron deforme*; B. ar, *Bombus ardens*; B. co, *Bombus consobrinus*; B. us, *Bombus ussurensis*.

## Supplementary Materials

The following are available online at https://www.mdpi.com/2072-6651/12/1/47/s1, Figure S1: Comparison of the relative transcription levels and TPM values of (A) arginine kinase, (B) defensin 1, (C) dipeptidyl peptidase 4, (D) hyaluronidase, (E) icarapin, (F) phospholipase A2, (G) serine protease inhibitor, (H) tachykinin, (I) vitallogenin and (J) neprilysin from *P. rothneyi*, *P. snelleni*, *E. decoratus* and *B. ardens*, Figure S2: Amino acid alignments of uncharacterized protein 1 from *V. crabro*, *V. simillima*, *P. varia*, *P. snelleni*, *B. consobrinus* and *M. banksi*, Figure S3: Amino acid alignments of uncharacterized protein 2 from *V. analis*, *V. dybowskii*, *P. varia*, *A. flavomarginatum* and *Crassostrea gigas*, Figure S4: Amino acid alignments of uncharacterized protein 3 from *V. dybowskii*, *B. consobrinus*, *B. ussurensis* and *Hevea brasiliensis*, Figure S5: Amino acid alignments of uncharacterized protein 4 from *B. ardens*, *B. ussurensis* and *Triticum aestivum*, Figure S6: Amino acid alignments of uncharacterized protein 5 from *P. varia*, *A. flavomarginatum* and *S. geminate*, Figure S7: Amino acid alignments of uncharacterized protein 6 from *V. dybowskii* and *A. flavomarginatum*, Figure S8: Amino acid alignments of carboxylesterase 6. (A) Alignment of amino acid sequences from *V. crabro*, *V. analis*, *V. dybowskii*, *V. simillima*, *P. varia*, *P. snelleni*, *P. rothneyi*, *A. flavomarginatum*, *Sphecidae* sp., *B. ussurensis* and *S. mimosarum*. (B) Phylogenetic analysis of carboxylesterase 6, Figure S9: Amino acid alignments of dipeptidyl peptidase 4. (A) Alignment of amino acid sequences from *V. crabro*, *V. analis*, *V. dybowskii*, *V. simillima*, *P. rothneyi*, *A. flavomarginatum*, *S. deforme*, *Sphecidae* sp., *B. ardens*, *B. consobrinus*, *B. ussurensis* and *P. tepidariorum*. (B) Phylogenetic analysis of dipeptidyl peptidase 4, Figure S10: Amino acid alignments of endocuticle structural glycoprotein. (A) Alignment of amino acid sequences from *V. crabro*, *V. analis*, *V. dybowskii*, *V. simillima*, *P. varia*, *P. snelleni*, *P. rothneyi*, *A. flavomarginatum*, *E. decoratus*, *S. deforme*, *B. consobrinus*, *B. ussurensis* and *Blattella germanica*. (B) Phylogenetic analysis of endocuticle structural glycoprotein, Figure S11: Amino acid alignments of icarapin. (A) Alignment of amino acid sequences from *V. crabro*, *V. analis*, V. dybowskii, *V. simillima*, *P. varia*, *P. snelleni*, *P. rothneyi*, *A. flavomarginatum*, *E. decoratus*, *S. deforme*, *Sphecidae* sp., *B. ardens*, *B. consobrinus*, *B. ussurensis* and *O. monticola*. (B) Phylogenetic analysis of icarapin, Figure S12: Amino acid alignments of major royal jelly protein. (A) Alignment of amino acid sequences from *P. varia*, *P. rothneyi*, *E. decoratus*, *S. deforme*, *B. ardens*, *B. consobrinus*, *B. ussurensis* and *P. tepidariorum*. (B) Phylogenetic analysis of major royal jelly protein, Figure S13: Amino acid alignments of phospholipase B. (A) Alignment of amino acid sequences from *V. analis*, *P. varia*, *P. snelleni*, *P. rothneyi*, *E. decoratus*, *Sphecidae* sp., *B. ardens*, *B. consobrinus*, *B. ussurensis* and *H. spadix*. B) Phylogenetic analysis of phospholipase B, Figure S14: Amino acid alignments of serine protease inhibitor. A) Alignment of amino acid sequences from *V. crabro*, *V. analis*, *V. dybowskii*, *V. simillima*, *P. varia*, *P. snelleni*, *P. rothneyi*, *A. flavomarginatum*, *E. decoratus*, *S. deforme*, *Sphecidae* sp., *B. ardens*, *B. consobrinus* and *B. germanica*. B() Phylogenetic analysis of serine protease inhibitor, Figure S15: Amino acid alignments of vitellogenin. (A) Alignment of amino acid sequences from *V. crabro*, *V. analis*, *V. dybowskii*, *V. simillima*, *P. varia*, *P. snelleni*, *P. rothneyi*, *A. flavomarginatum*, *Sphecidae* sp., *B. ardens*, *B. consobrinus*, *B. ussurensis* and *B. germanica*. (B) Phylogenetic analysis of vitellogenin, Figure S16: Amino acid alignments of acid phosphatase. (A) Alignment of amino acid sequences from *V. crabro*, *V. analis*, *V. dybowskii*, *P. varia*, *P. snelleni*, *A. flavomarginatum*, *S. deforme*, *Sphecidae* sp., *B. ardens*, *B. consobrinus*, *B. ussurensis* and *H. sapiens*. (B) Phylogenetic analysis of acid phosphatase, Figure S17: Amino acid alignments of defensin 2. (A) Alignment of amino acid sequences from *P. varia*, *P. snelleni*, *P. rothneyi*, *A. flavomarginatum*, *E. decoratus*, *Sphecidae* sp. and *H. sapiens*. (B) Phylogenetic analysis of defensin 2, Figure S18: Amino acid alignments of hyaluronidase. (A) Alignment of amino acid sequences from *V. crabro*, *V. analis*, *V. dybowskii*, *V. simillima*, *P. varia*, *P. snelleni*, *P. rothneyi*, *A. flavomarginatum*, *S. deforme*, *Sphecidae* sp., *B. ardens*, *B. ussurensis* and *H. sapiens*. (B) Phylogenetic analysis of hyaluronidase, Figure S19: Amino acid alignments of mastoparan. (A) Alignment of amino acid sequences from *V. crabro*, *V. analis*, *P. snelleni*, *P. rothneyi*, *A. flavomarginatum*, *S. deforme*, *B. ussurensis* and uncultured bacterium. (B) Phylogenetic analysis of mastoparan, Figure S20: Amino acid alignments of metalloproteinase. (A) Alignment of amino acid sequences from *V. crabro*, *V. analis*, *V. dybowskii*, *V. simillima*, *P. varia*, *P. snelleni*, *P. rothneyi*, *A. flavomarginatum*, *E. decoratus*, *S. deforme*, *Sphecidae* sp., *B. ardens*, *B. ussurensis* and *H. sapiens*. (B) Phylogenetic analysis of metalloproteinase, Figure S21: Amino acid alignments of neprilysin. (A) Alignment of amino acid sequences from *A. flavomarginatum*, *E. decoratus*, *B. consobrinus* and *B. germanica*. (B) Phylogenetic analysis of neprilysin, Figure S22: Amino acid alignments of phospholipase A1. (A) Alignment of amino acid sequences from *V. crabro*, *V. analis*, *V. dybowskii*, *V. simillima*, *P. varia*, *P. rothneyi*, *E. decoratus*, *B. consobrinus*, *B. ussurensis* and *H. spadix*. (B) Phylogenetic analysis of phospholipase A1, Figure S23: Amino acid alignments of serine carboxypeptidase. (A) Alignment of amino acid sequences from *V. crabro*, *V. analis*, *P. varia*, *P. rothneyi*, *A. flavomarginatum*, *E. decoratus*, *S. deforme*, *Sphecidae* sp., *B. ardens*, *B. consobrinus* and *B. germanica*. (B) Phylogenetic analysis of serine carboxypeptidase, Figure S24: Amino acid alignments of serine protease. (A) Alignment of amino acid sequences from *V. crabro*, *V. analis*, *V. dybowskii*, *P. varia*, *P. snelleni*, *P. rothneyi*, *A. flavomarginatum*, *E. decorates*, *S. deforme*, *Sphecidae* sp., *B. ardens*, *B. ussurensis* and *H. sapiens*. (B) Phylogenetic analysis of serine protease, Figure S25: Amino acid alignments of arginine kinase. (A) Alignment of amino acid sequences from *V. crabro*, *V. analis*, *V. dybowskii*, *V. simillima*, *P. varia*, *P. snelleni*, *A. flavomarginatum*, *E. decoratus*, *S. deforme*, *Sphecidae* sp., *B. consobrinus* and *Hadrurus spadix*. (B) Phylogenetic analysis of arginine kinase, Figure S26: Amino acid alignments of defensin 1. (A) Alignment of amino acid sequences from *V. crabro*, *V. analis*, *V. dybowskii*, *P. varia*, *P. rothneyi*, *A. flavomarginatum*, *Sphecidae* sp., *B. ardens*, *B. consobrinus*, *B. ussurensis* and *H. sapiens*. (B) Phylogenetic analysis of defensin 1, Figure S27: Amino acid alignments of phospholipase A2. (A) Alignment of amino acid sequences from *V. crabro*, *V. analis*, *V. dybowskii*, *V. simillima*, *P. varia*, *P. rothneyi*, *A. flavomarginatum*, *Sphecidae* sp., *B. ardens*, *B. ussurensis* and *H. sapiens*. (B) Phylogenetic analysis of phospholipase A2, Figure S28: Amino acid alignments of tachykinin. (A) Alignment of amino acid sequences from *V. analis*, *V. dybowskii*, *P. varia*, *P. snelleni*, *P. rothneyi*, *E. decorates*, *Sphecidae* sp., *B. ardens*, *B. consobrinus*, *B. ussurensis*, and *B. germanica*. (B) Phylogenetic analysis of tachykinin, Figure S29: Amino acid alignments of venom allergen 5. (A) Alignment of amino acid sequences from *V. crabro*, *V. analis*, *V. simillima*, *P. snelleni*, *P. rothneyi*, *A. flavomarginatum*, *S. deforme*, *Sphecidae* sp., *B. ussurensis* and *Varroa destructor*. (B) Phylogenetic analysis of venom allergen 5, Table S1: Annotation of top 100 highly expressed genes in the venom gland of *Vespa analis*, Table S2: Annotation of top 100 highly expressed genes in the venom gland of *Vespa crabro*, Table S3: Annotation of top 100 highly expressed genes in the venom gland of *Vespa dybowskii*, Table S4: Annotation of top 100 highly expressed genes in the venom gland of *Vespa simillima*, Table S5: Annotation of top 100 highly expressed genes in the venom gland of *Parapolybia varia*, Table S6: Annotation of top 100 highly expressed genes in the venom gland of *Polistes rothneyi*, Table S7: Annotation of top 100 highly expressed genes in the venom gland of *Polistes snelleni*, Table S8: Annotation of top 100 highly expressed genes in the venom gland of *Eumenes decoratus*, Table S9: Annotation of top 100 highly expressed genes in the venom gland of *Sphecidae* sp., Table S10: Annotation of top 100 highly expressed genes in the venom gland of *Anterhynchium flavomarginatum*, Table S11: Annotation of top 100 highly expressed genes in the venom gland of *Sceliphron deforme*, Table S12: Annotation of top 100 highly expressed genes in the venom gland of *Bombus ardens*, Table S13: Annotation of top 100 highly expressed genes in the venom gland of *Bombus consobrinus*, Table S14: Annotation of top 100 highly expressed genes in the venom gland of *Bombus ussurensis*, Table S15: TPM values of reference housekeeping gene dimethyladenosine transferase in 14 Aculeate bee and wasp species, Table S16: Primers used in quantitative real-time PCR.
