# Supplementary material for: Characterization of Venom Components and Their Phylogenetic Properties in Some Aculeate Bumblebees and Wasps
Source: Toxins (Basel). 2020 Jan 14;12(1):47. doi: 10.3390/toxins12010047 (PMC7020409; doi:10.3390/toxins12010047)
Supplement: Supplementary file 1 [file toxins-12-00047-s001.pdf]

Supplementary Materials: Characterization of Venom Components and Their Phylogenetic Properties in Some Aculeate Bumblebees and Wasps

Kyungjae Andrew Yoon, Kyungmun Kim, Woo-Jin Kim, Woo Young Bang, Neung-Ho Ahn, Chang-Hwan Bae, Joo-Hong Yeo and Si Hyeock Lee

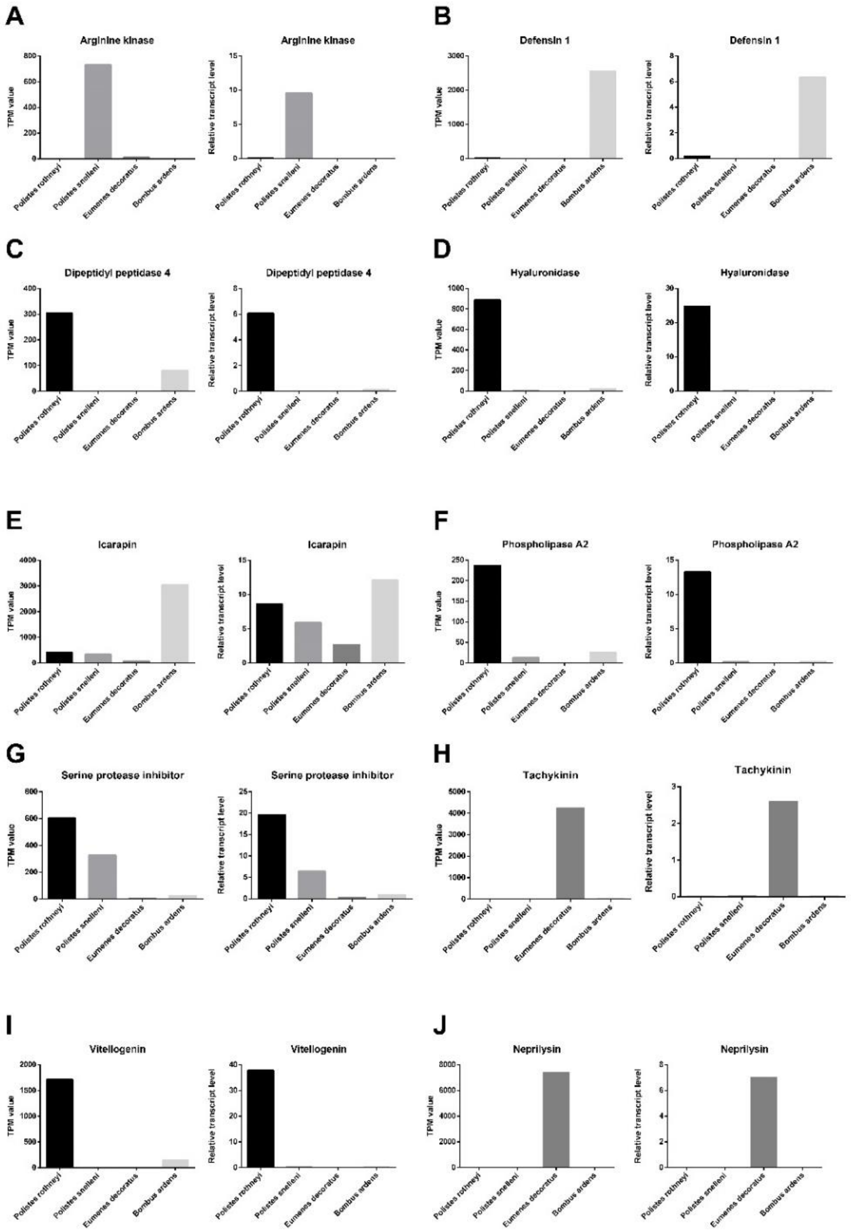

Figure S1. Comparison of the relative transcription levels and TPM values of A) arginine kinase, B) defensin 1, C) dipeptidyl peptidase 4, D) hyaluronidase, E) icarapin, F) phospholipase A2, G) serine protease inhibitor, H) tachykinin, I) vitallogenin and J) neprilysin from *P. rothneyi*, *P. snelleni*, *E. decoratus* and *B. ardens*.

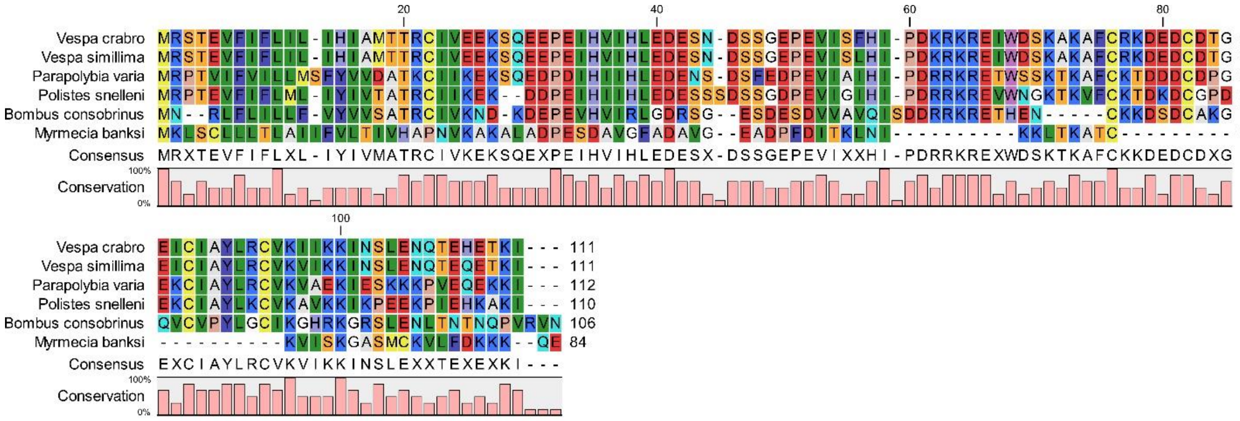

Figure S2. Amino acid alignments of uncharacterized protein 1 from *V. crabro*, *V. simillima*, *P. varia*, *P. snelleni*, *B. consobrinus* and *M. banksi*.

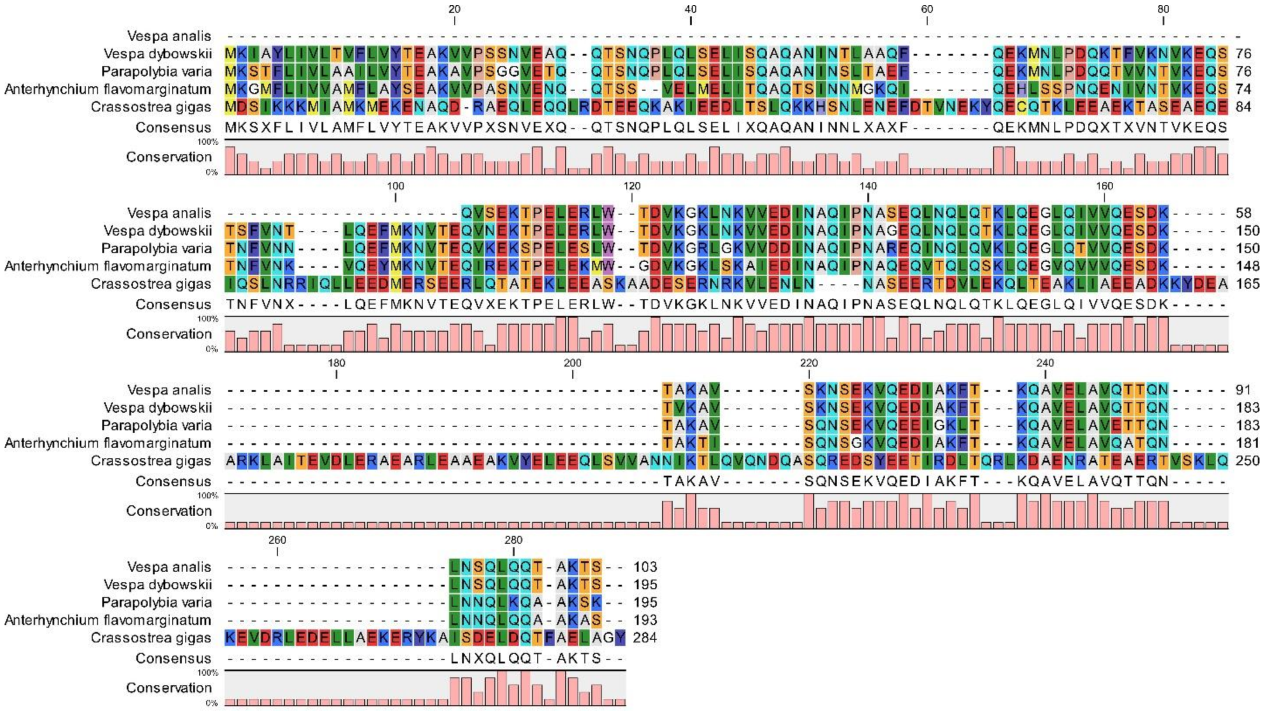

Figure S3. Amino acid alignments of uncharacterized protein 2 from *V. analis*, *V. dybowskii*, *P. varia*, *A. flavomarginatum* and *Crassostrea gigas*.

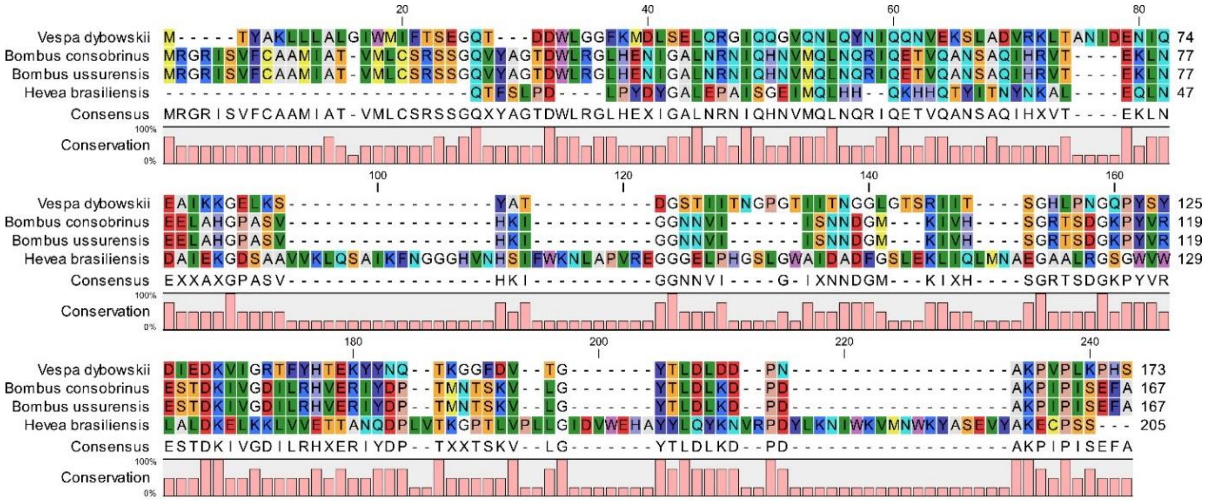

Figure S4. Amino acid alignments of uncharacterized protein 3 from *V. dybowskii*, *B. consobrinus*, *B. ussurensis* and *Hevea brasiliensis*.

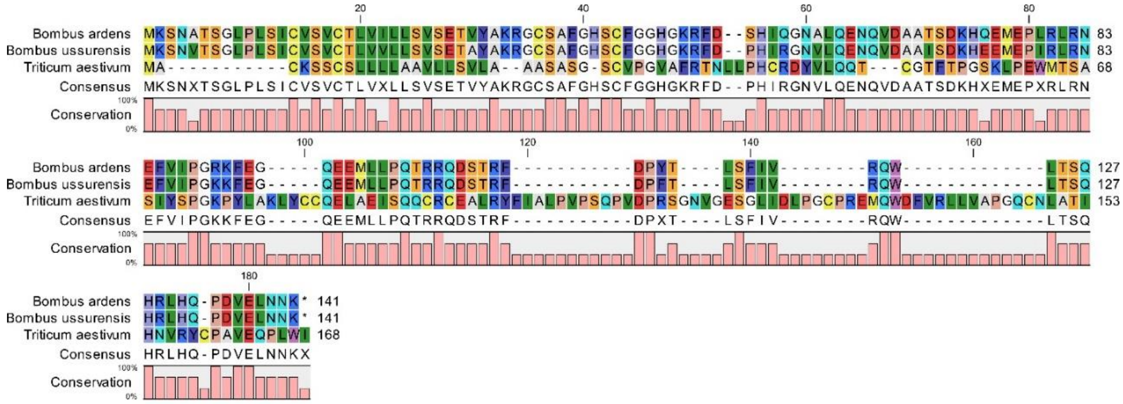

Figure S5. Amino acid alignments of uncharacterized protein 4 from *B. ardens*, *B. ussurensis* and *Triticum aestivum*.

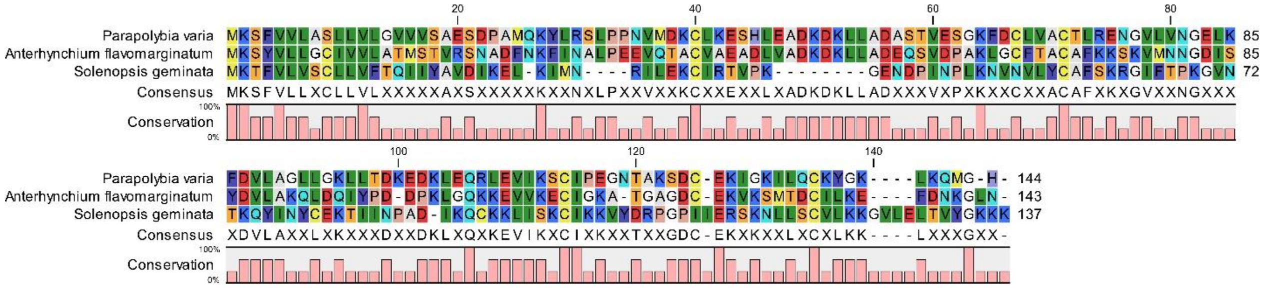

Figure S6. Amino acid alignments of uncharacterized protein 5 from *P. varia*, *A. flavomarginatum* and *S. geminate*.

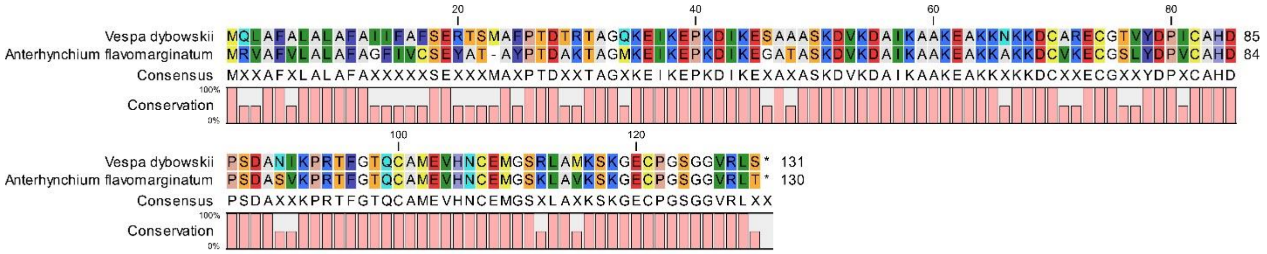

Figure S7. Amino acid alignments of uncharacterized protein 6 from *V. dybowskii* and *A. flavomarginatum*.

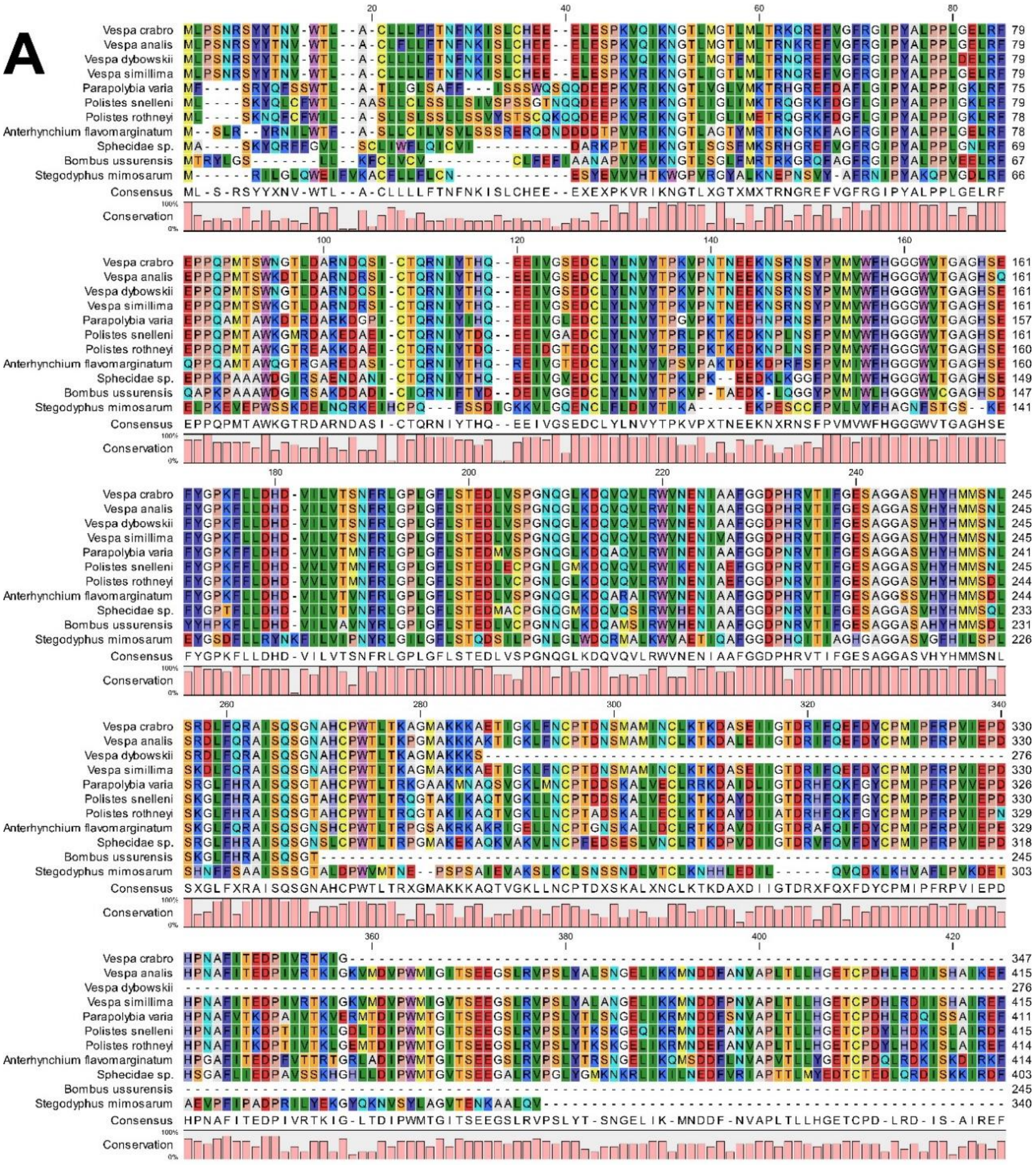

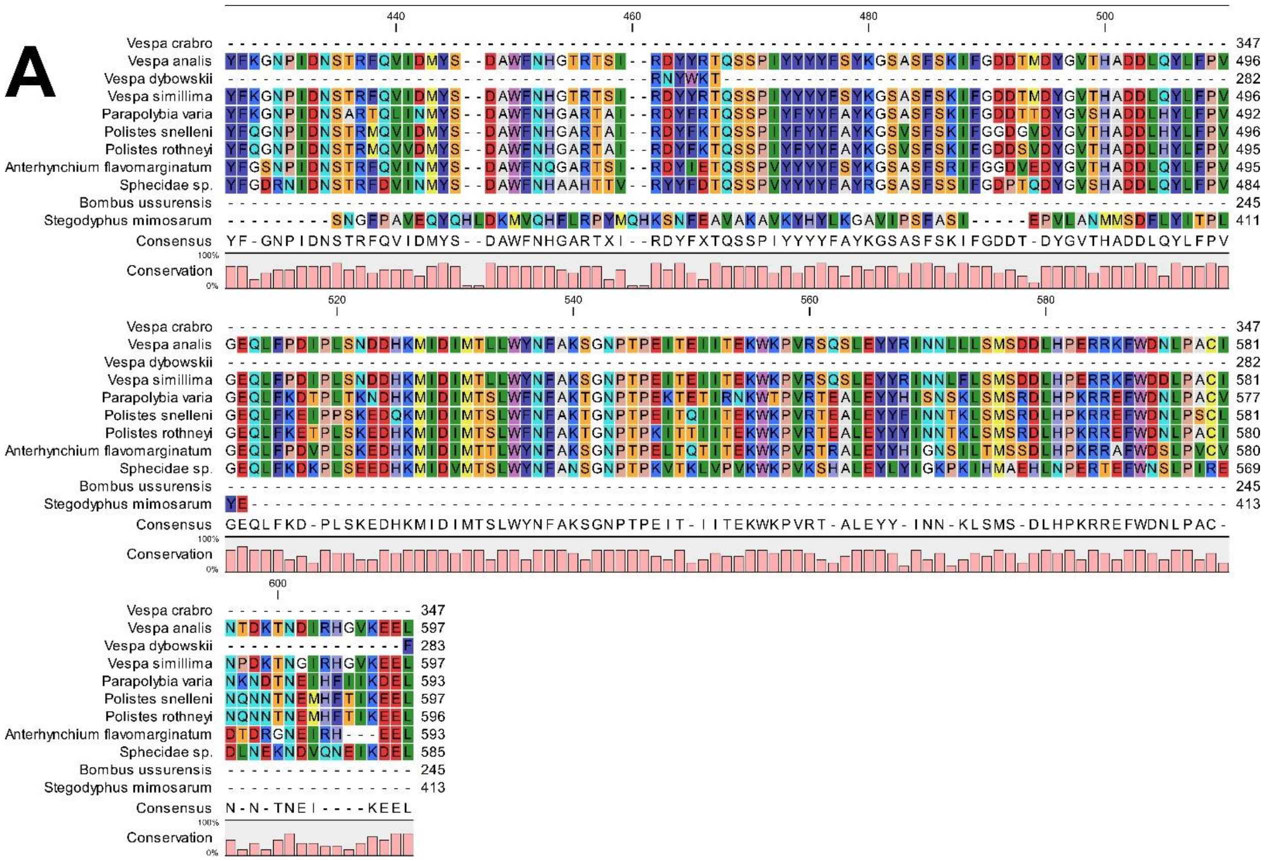
A
Vespa crabro
Vespa analis
Vespa dybowskii
Vespa simillima
Parapolybia varia
Polistes snelleni
Polistes rothneyi
Anterhynchium flavomarginatum
Sphecidae sp.
Bombus ussurensis
Stegodyphus mimosarum
Consensus
Conservation

Figure S8B

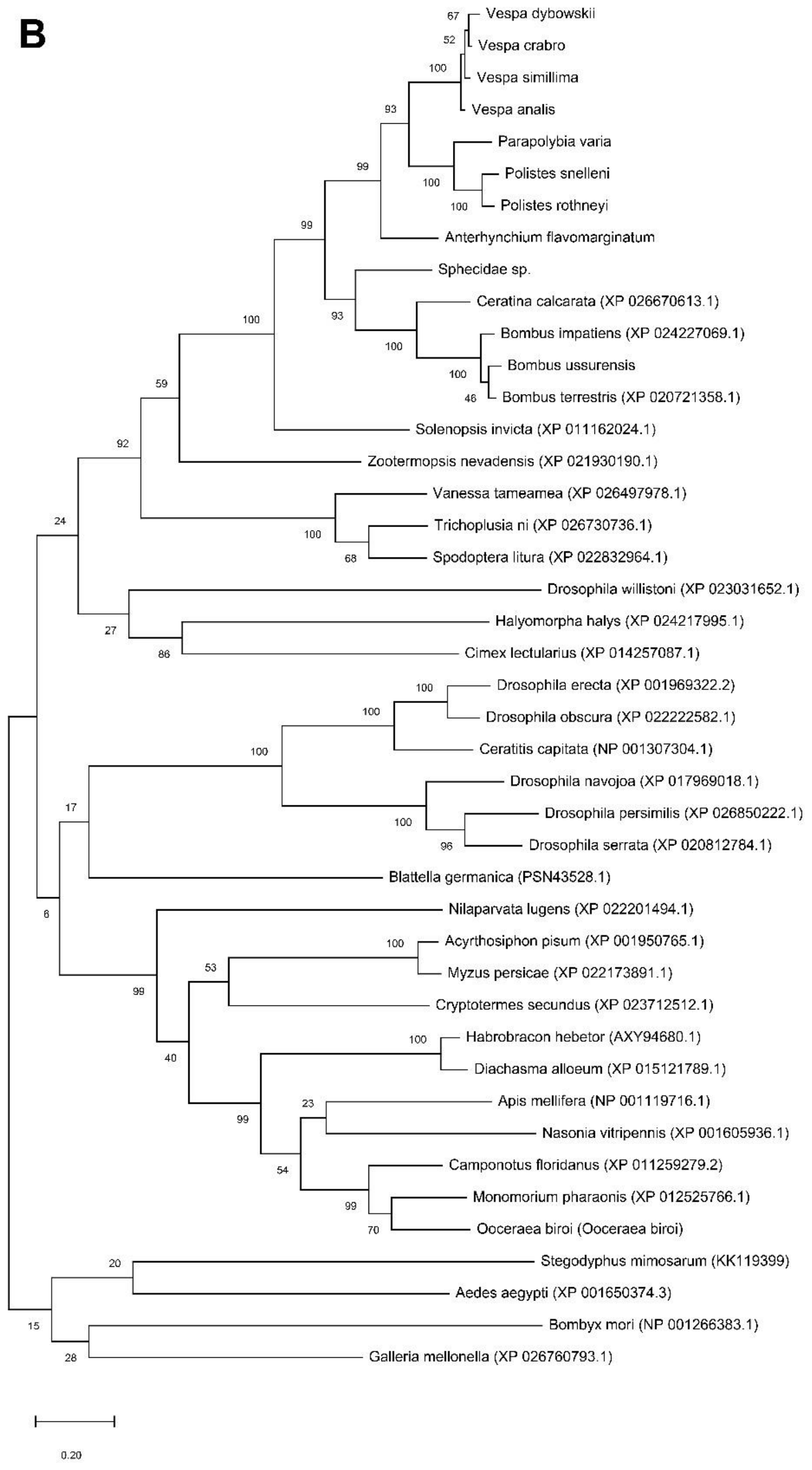

Figure S8. Amino acid alignments of carboxylesterase 6. A) Alignment of amino acid sequences from *V. crabro, V. analis, V. dybowskii, V. simillima, P. varia, P. snelleni, P. rothneyi, A. flavomarginatum, Sphecidae* sp., *B. ussurensis* and *S. mimosarum*. B) Phylogenetic analysis of carboxylesterase 6.

A

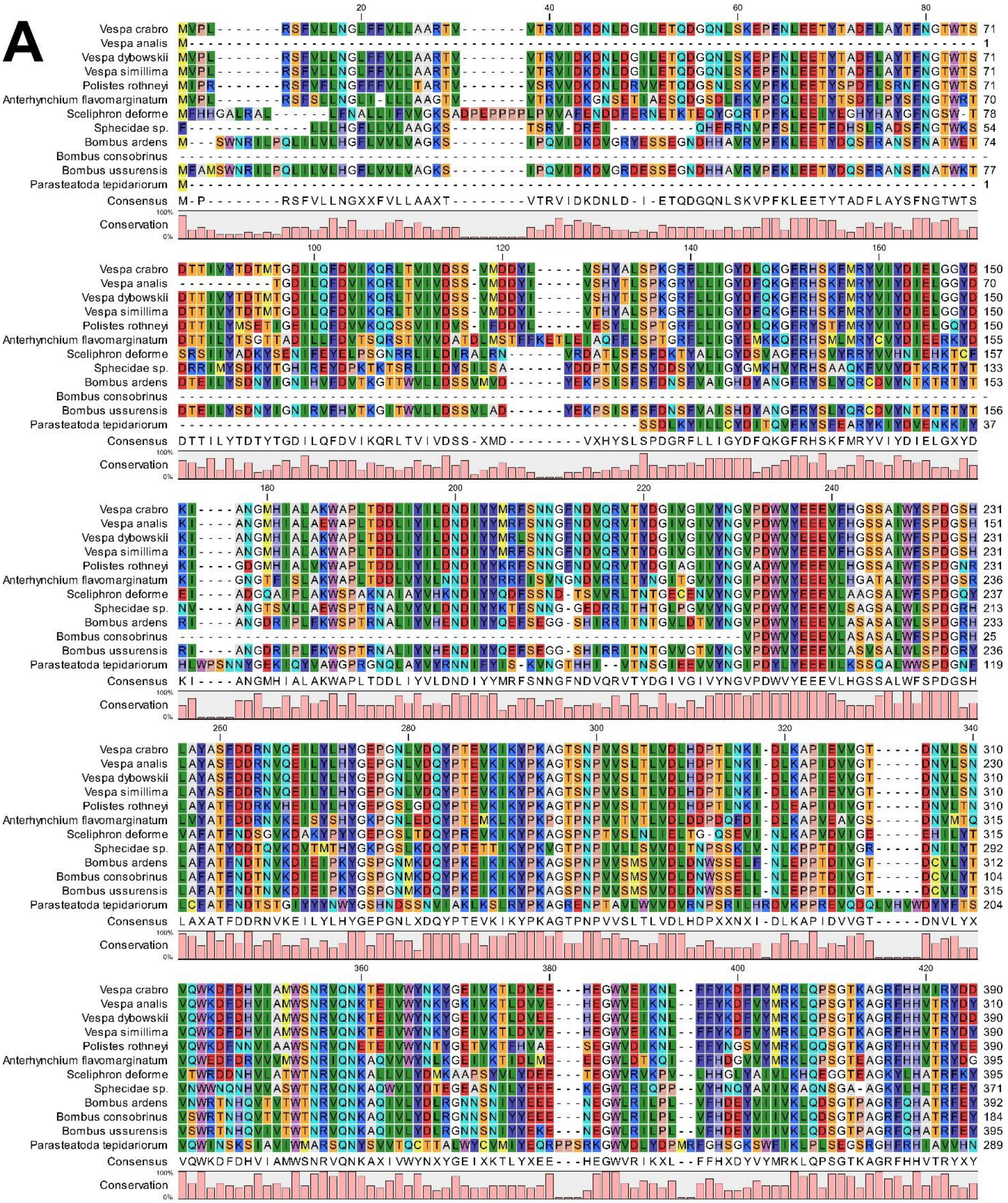
Vespa crabro
Vespa analis
Vespa dybowskii
Vespa simillima
Polistes rothneyi
Anterhynchium flavomarginatum
Sceliphron deforme
Sphecidae sp.
Bombus ardens
Bombus consobrinus
Bombus ussurensis
Parasteatoda tepidariorum
Consensus
Conservation

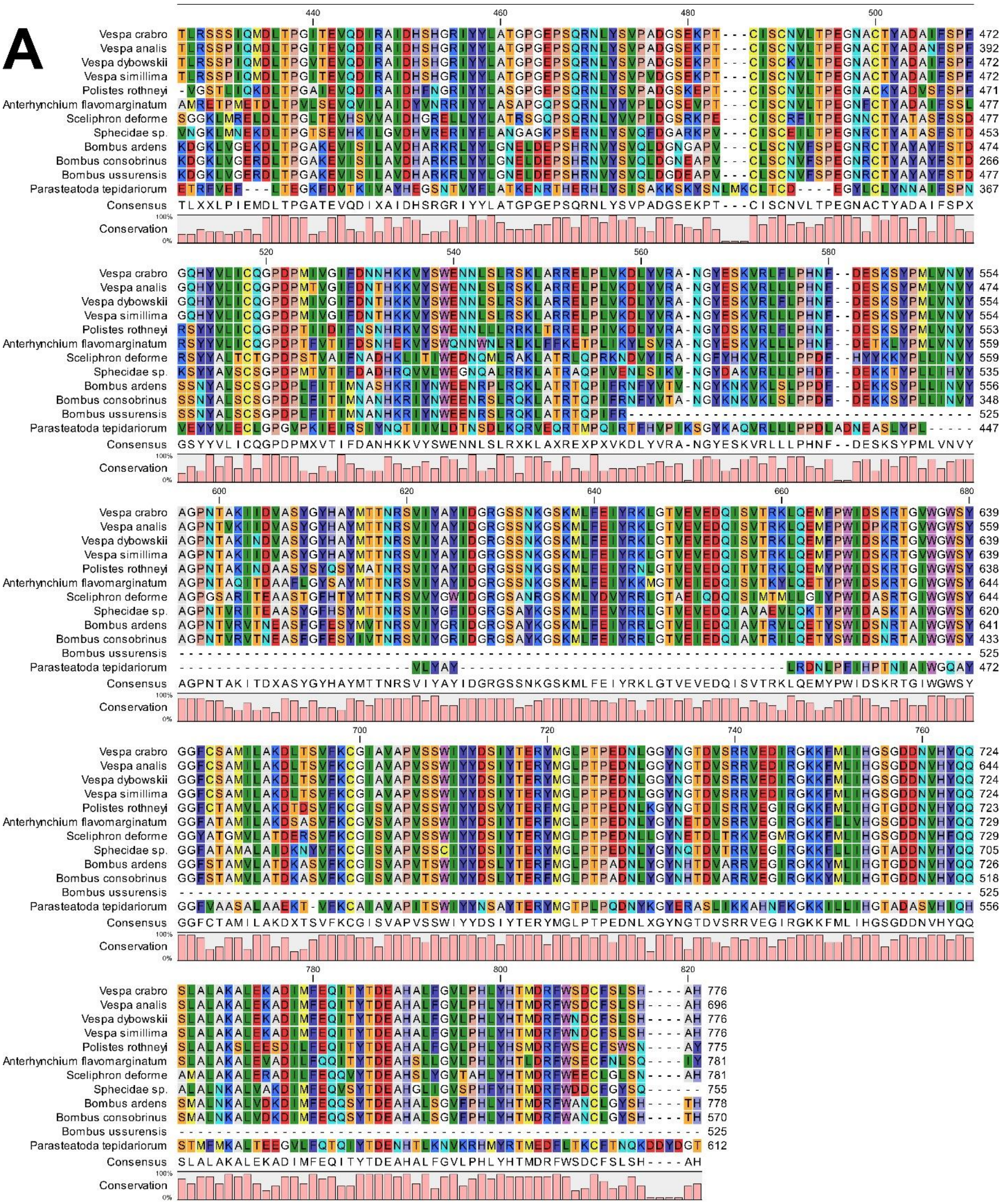
A
Vespa crabro
Vespa analis
Vespa dybowskii
Vespa simillima
Polistes rothneyi
Anterhynchium flavomarginatum
Sceliphron deforme
Sphecidae sp.
Bombus ardens
Bombus consobrinus
Bombus ussurensis
Parasteatoda tepidariorum
Consensus
Conservation

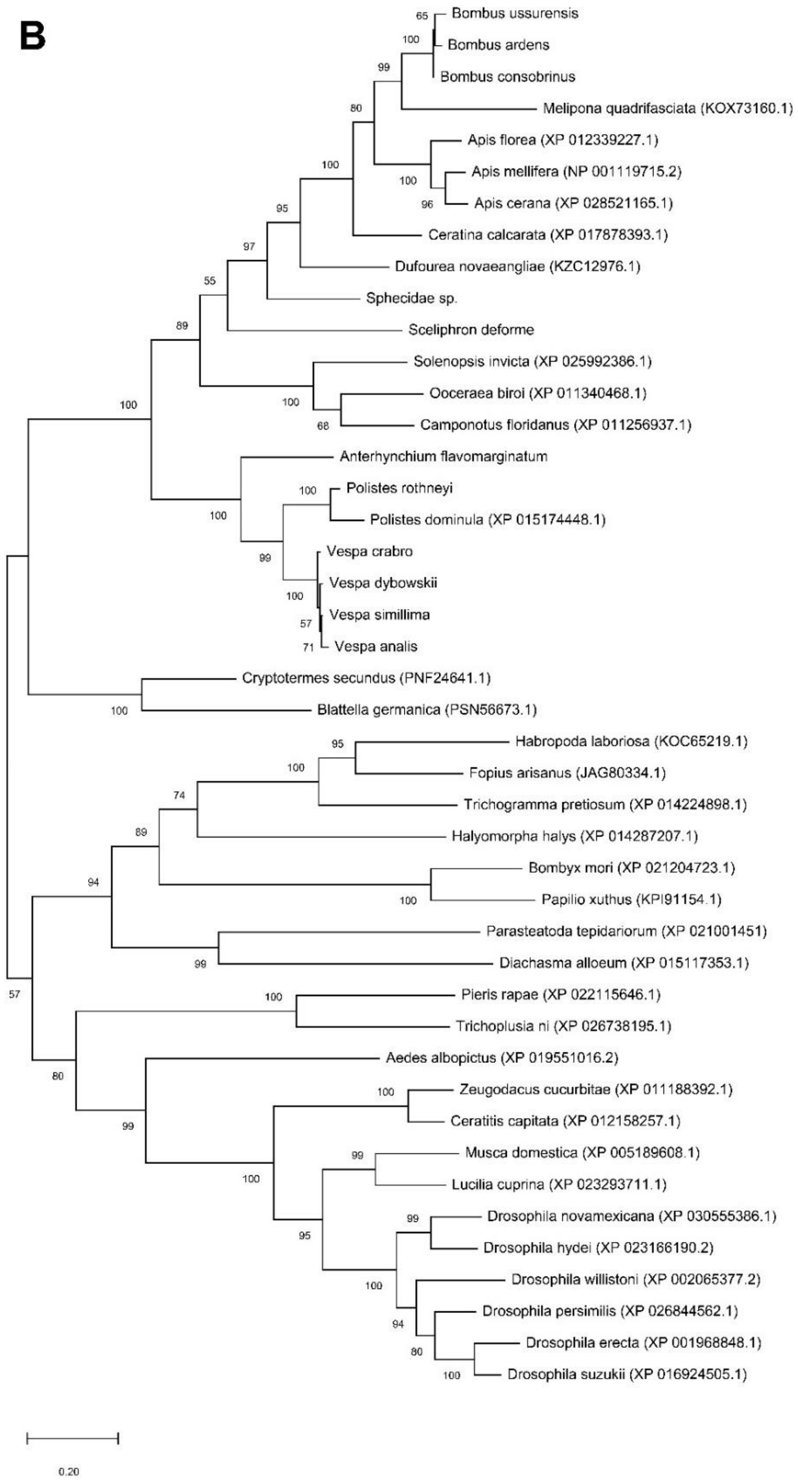

Figure S9. Amino acid alignments of dipeptidyl peptidase 4. A) Alignment of amino acid sequences from *V. crabro*, *V. analis*, *V. dybowskii*, *V. simillima*, *P. rothneyi*, *A. flavomarginatum*, *S. deforme*, *Sphecidae* sp., *B. ardens*, *B. consobrinus*, *B. ussurensis* and *P. tepidariorum*. B) Phylogenetic analysis of dipeptidyl peptidase 4.

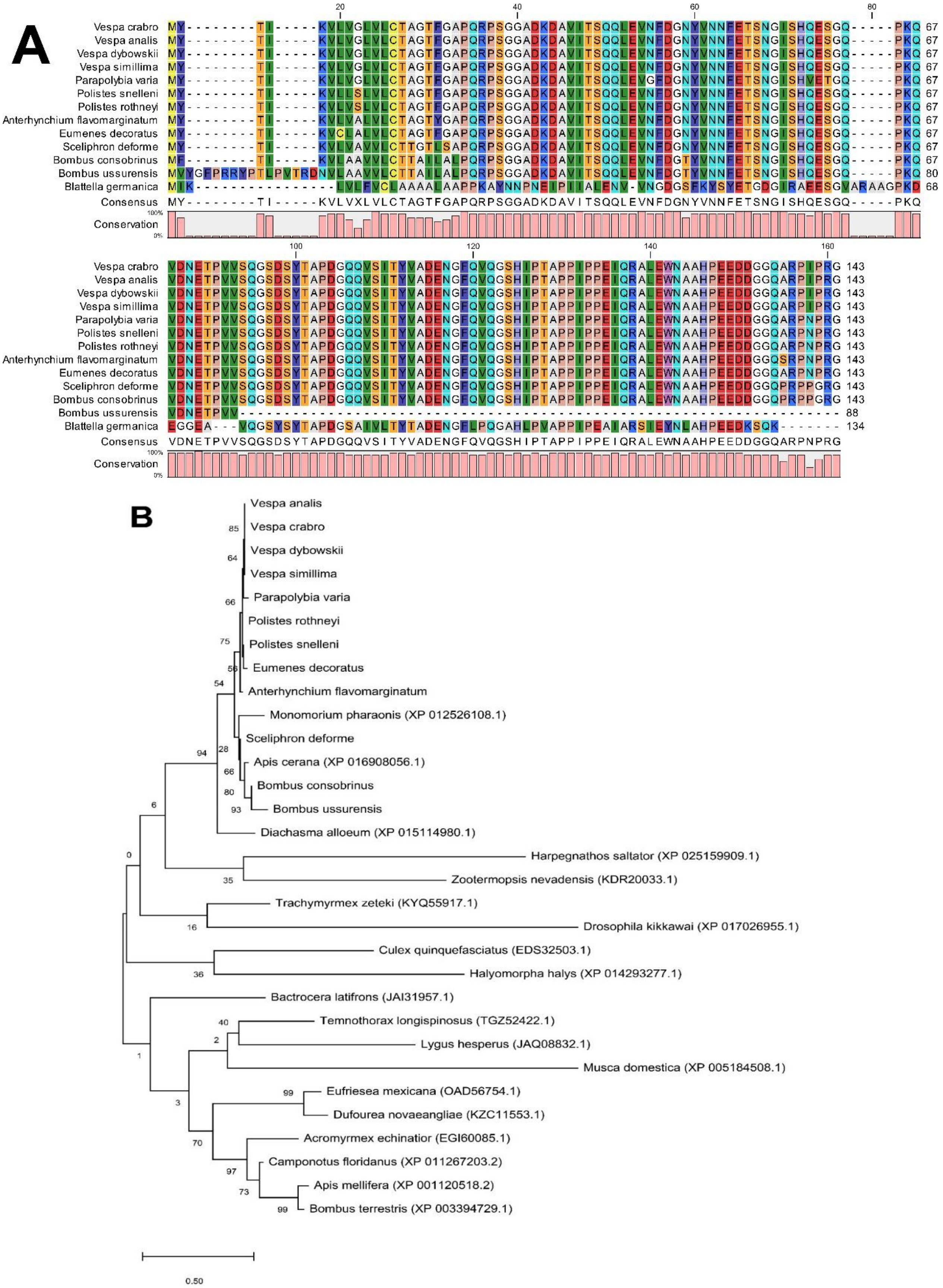
A
Vespa crabro
Vespa analis
Vespa dybowskii
Vespa simillima
Parapolybia varia
Polistes snelleni
Polistes rothneyi
Anterhynchium flavomarginatum
Eumenes decoratus
Sceliphron deforme
Bombus consobrinus
Bombus ussurensis
Blattella germanica
Consensus
Conservation
B
Vespa analis
Vespa crabro
Vespa dybowskii
Vespa simillima
Parapolybia varia
Polistes rothneyi
Polistes snelleni
Eumenes decoratus
Anterhynchium flavomarginatum
Monomorium pharaonis (XP 012526108.1)
Sceliphron deforme
Apis cerana (XP 016908056.1)
Bombus consobrinus
Bombus ussurensis
Diachasma alloeum (XP 015114980.1)
Harpegnathos saltator (XP 025159909.1)
Zootermopsis nevadensis (KDR20033.1)
Trachymyrmex zeteki (KYQ55917.1)
Drosophila kikkawai (XP 017026955.1)
Culex quinquefasciatus (EDS32503.1)
Halyomorpha halys (XP 014293277.1)
Bactrocera latifrons (JAI31957.1)
Temnothorax longispinosus (TGZ52422.1)
Lygus hesperus (JAQ08832.1)
Musca domestica (XP 005184508.1)
Eufriesea mexicana (OAD56754.1)
Dufourea novaeangliae (KZC11553.1)
Acromyrmex echinatior (EGI60085.1)
Camponotus floridanus (XP 011267203.2)
Apis mellifera (XP 001120518.2)
Bombus terrestris (XP 003394729.1)
0.50

Figure S10. Amino acid alignments of endocuticle structural glycoprotein. A) Alignment of amino acid sequences from *V. crabro*, *V. analis*, *V. dybowskii*, *V. simillima*, *P. varia*, *P. snelleni*, *P. rothneyi*, *A. flavomarginatum*, *E. decoratus*, *S. deforme*, *B. consobrinus*, *B. ussurensis* and *Blattella germanica*. B) Phylogenetic analysis of endocuticle structural glycoprotein.

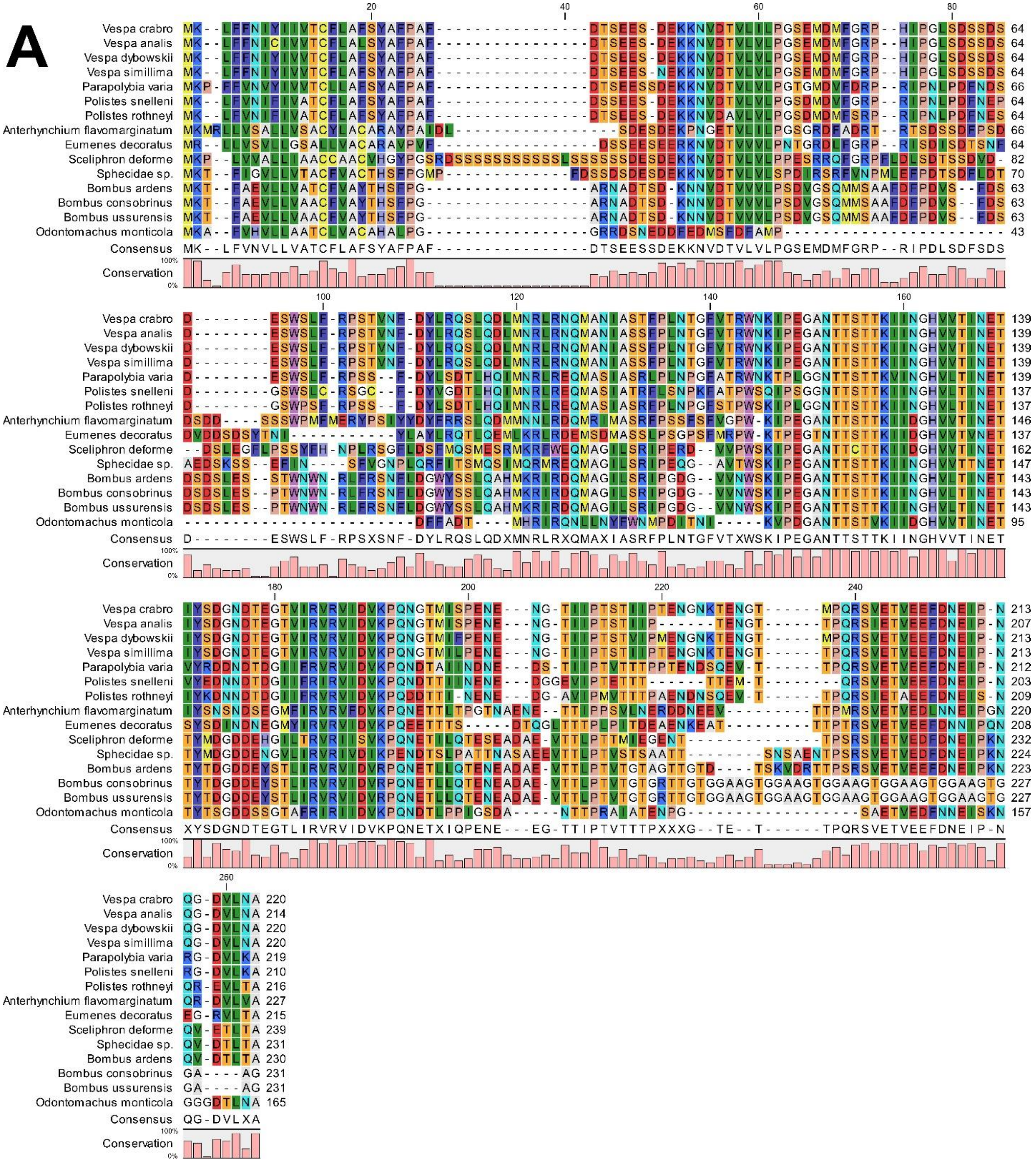

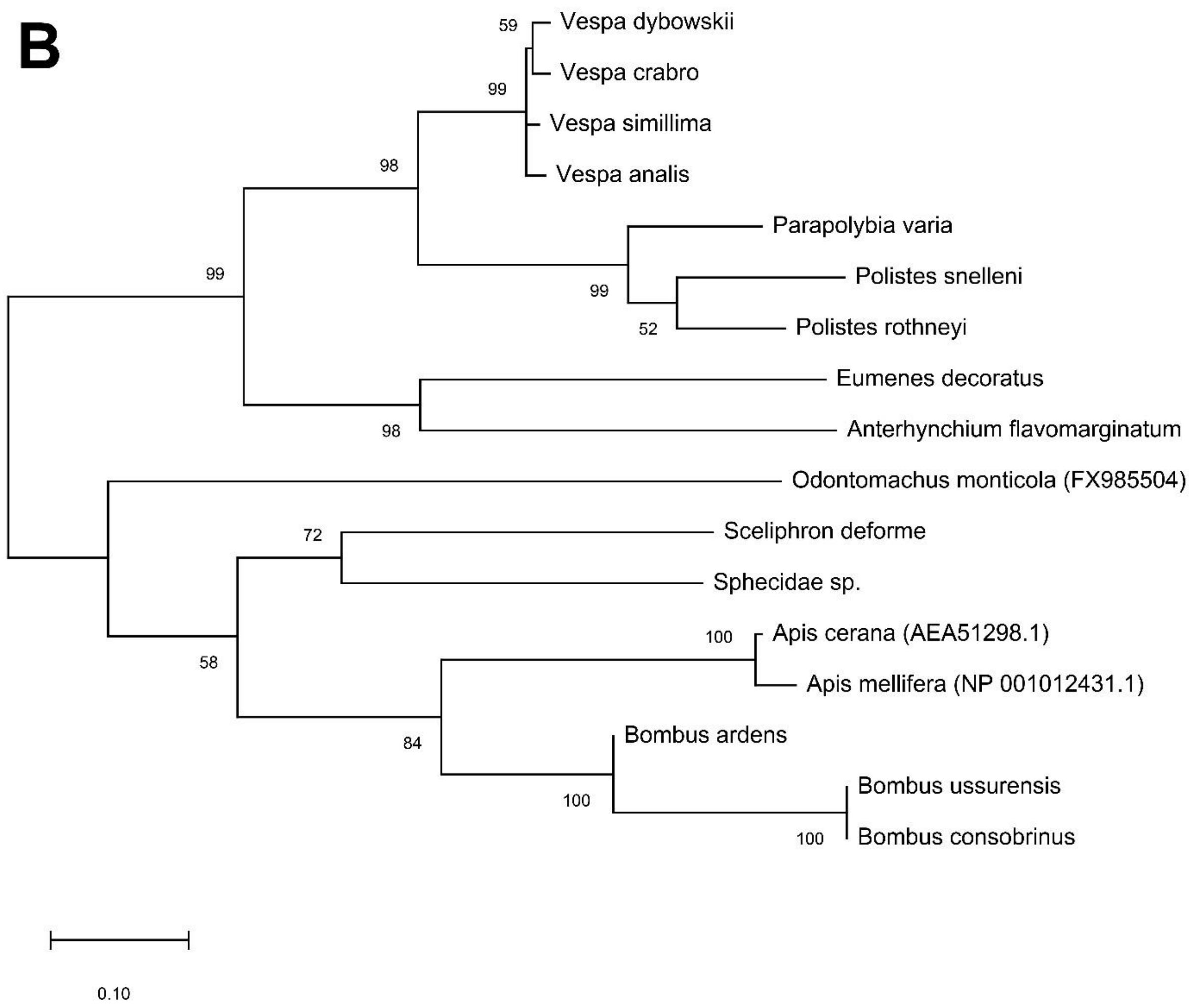

Figure S11. Amino acid alignments of icarapin. A) Alignment of amino acid sequences from *V. crabro*, *V. analis*, V. dybowskii, *V. simillima*, *P. varia*, *P. snelleni*, *P. rothneyi*, *A. flavomarginatum*, *E. decoratus*, *S. deforme*, *Sphecidae* sp., *B. ardens*, *B. consobrinus*, *B. ussurensis* and *O. monticola*. B) Phylogenetic analysis of icarapin.

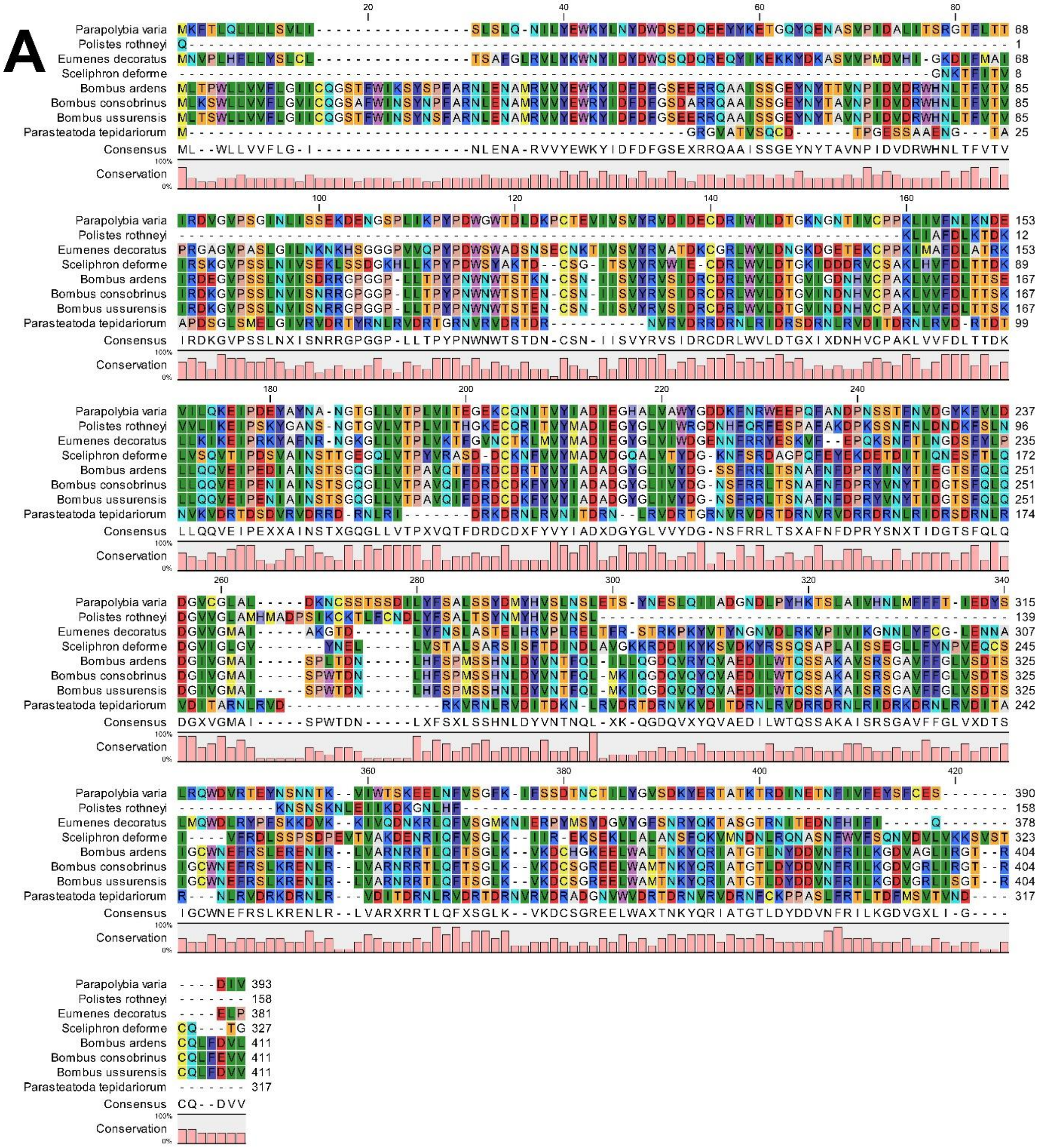

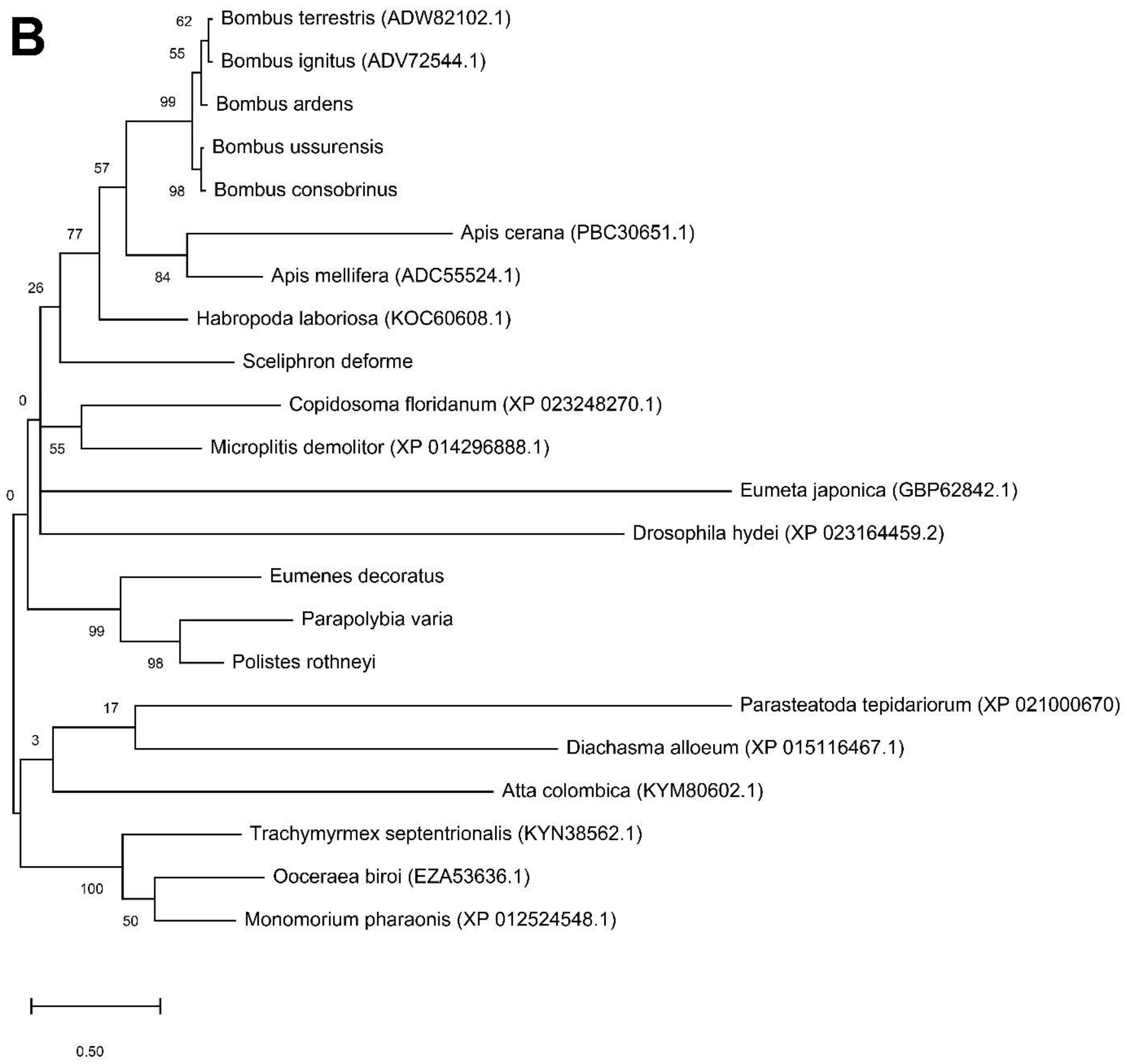

Figure S12. Amino acid alignments of major royal jelly protein. A) Alignment of amino acid sequences from *P. varia, P. rothneyi, E. decoratus, S. deforme, B. ardens, B. consobrinus, B. ussurensis* and *P. tepidariorum*. B) Phylogenetic analysis of major royal jelly protein.

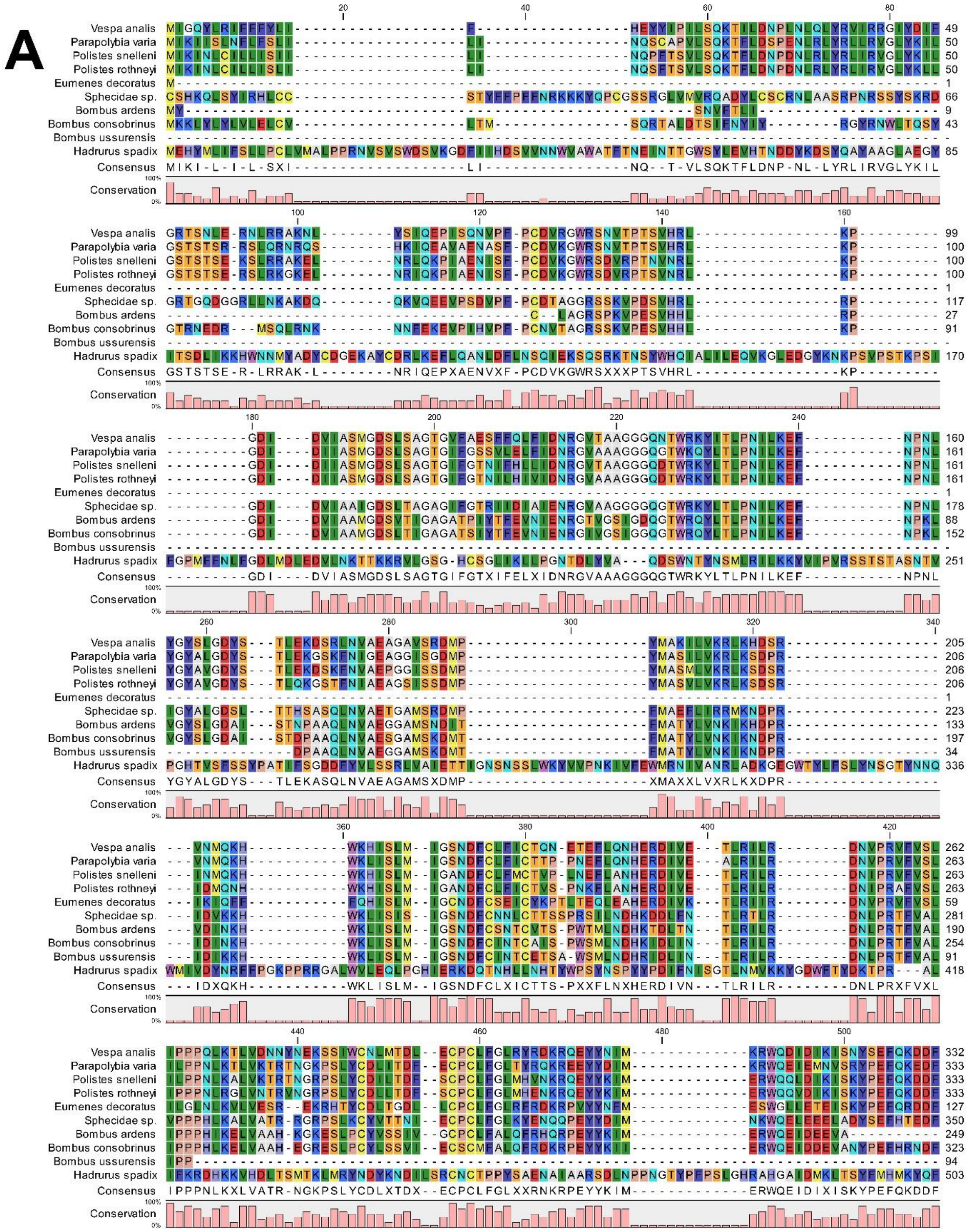
A
Vespa analis
Parapolybia varia
Polistes snelleni
Polistes rothneyi
Eumenes decoratus
Sphecidae sp.
Bombus ardens
Bombus consobrinus
Bombus ussurensis
Hadrurus spadix
Consensus
Conservation

Figure S13A-2

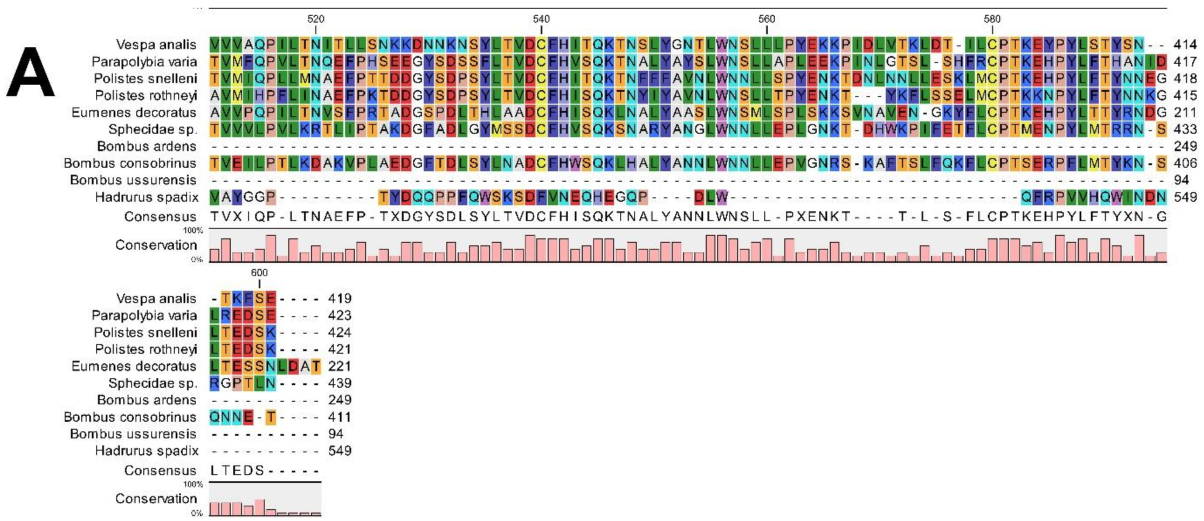

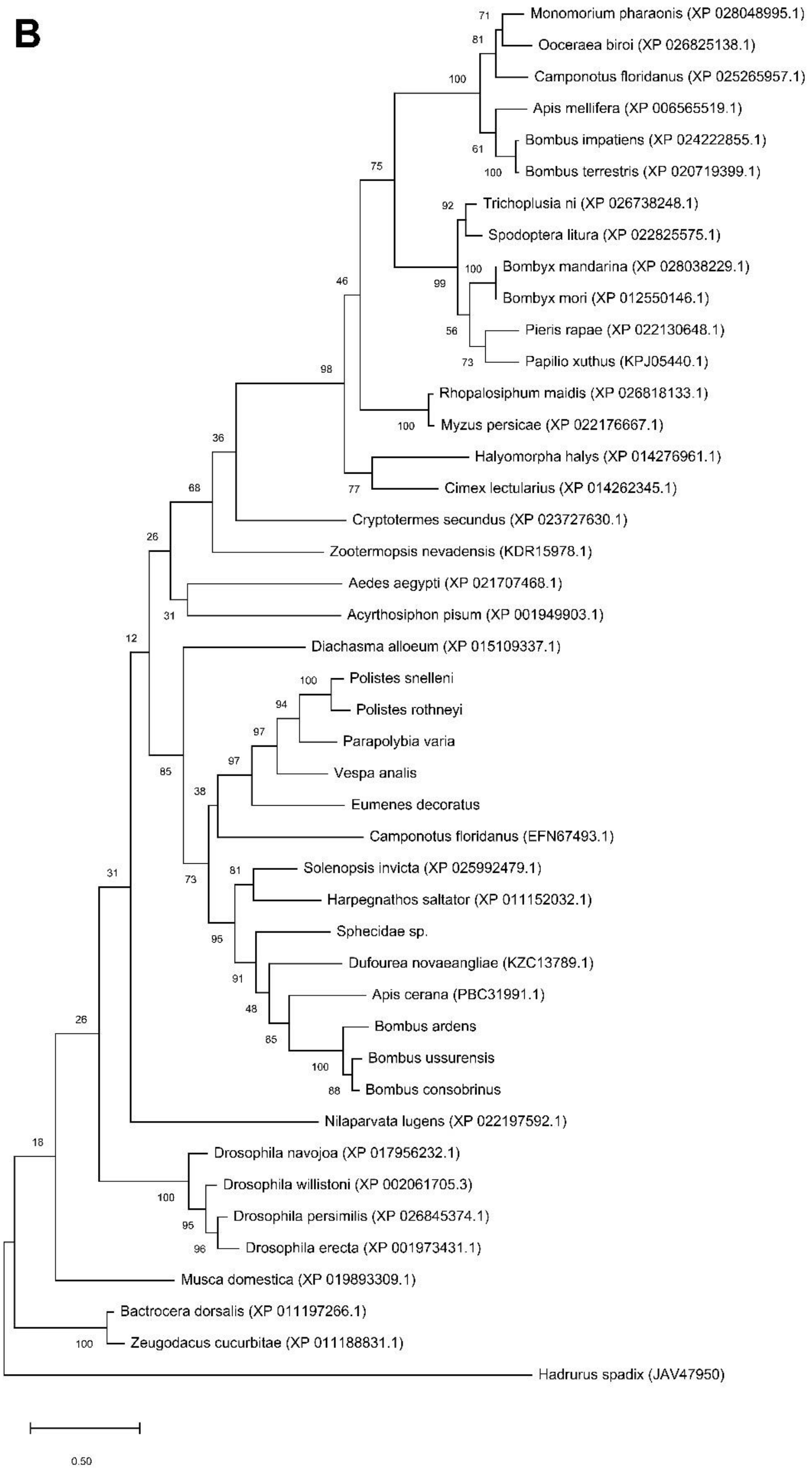

Figure S13. Amino acid alignments of phospholipase B. A) Alignment of amino acid sequences from *V. analis*, *P. varia*, *P. snelleni*, *P. rothneyi*, *E. decoratus*, *Sphecidae* sp., *B. ardens*, *B. consobrinus*, *B. ussurensis* and *H. spadix*. B) Phylogenetic analysis of phospholipase B.

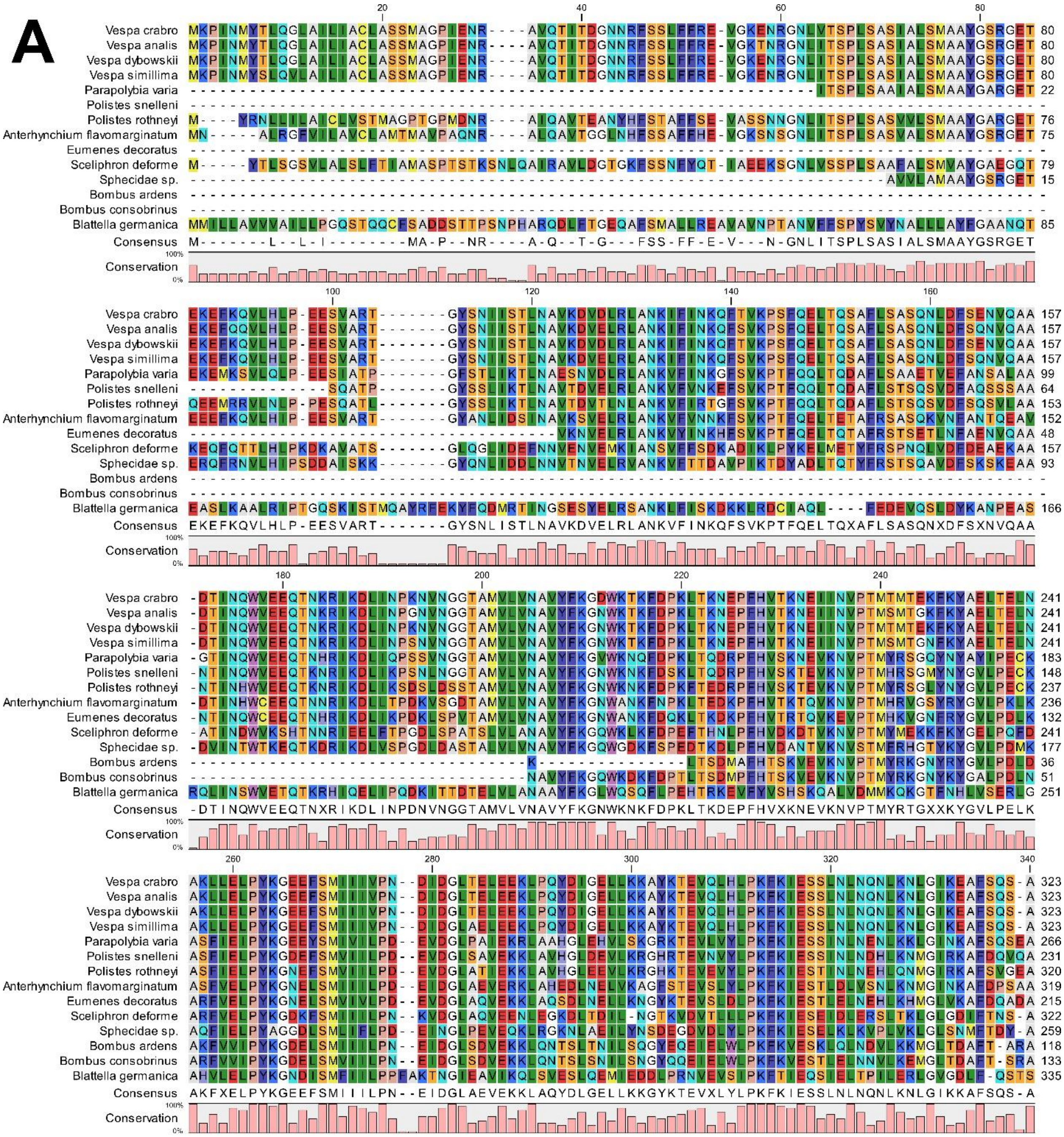
A
Vespa crabro
Vespa analis
Vespa dybowskii
Vespa simillima
Parapolybia varia
Polistes snelleni
Polistes rothneyi
Anterhynchium flavomarginatum
Eumenes decoratus
Sceliphron deforme
Sphecidae sp.
Bombus ardens
Bombus consobrinus
Blattella germanica
Consensus
Conservation

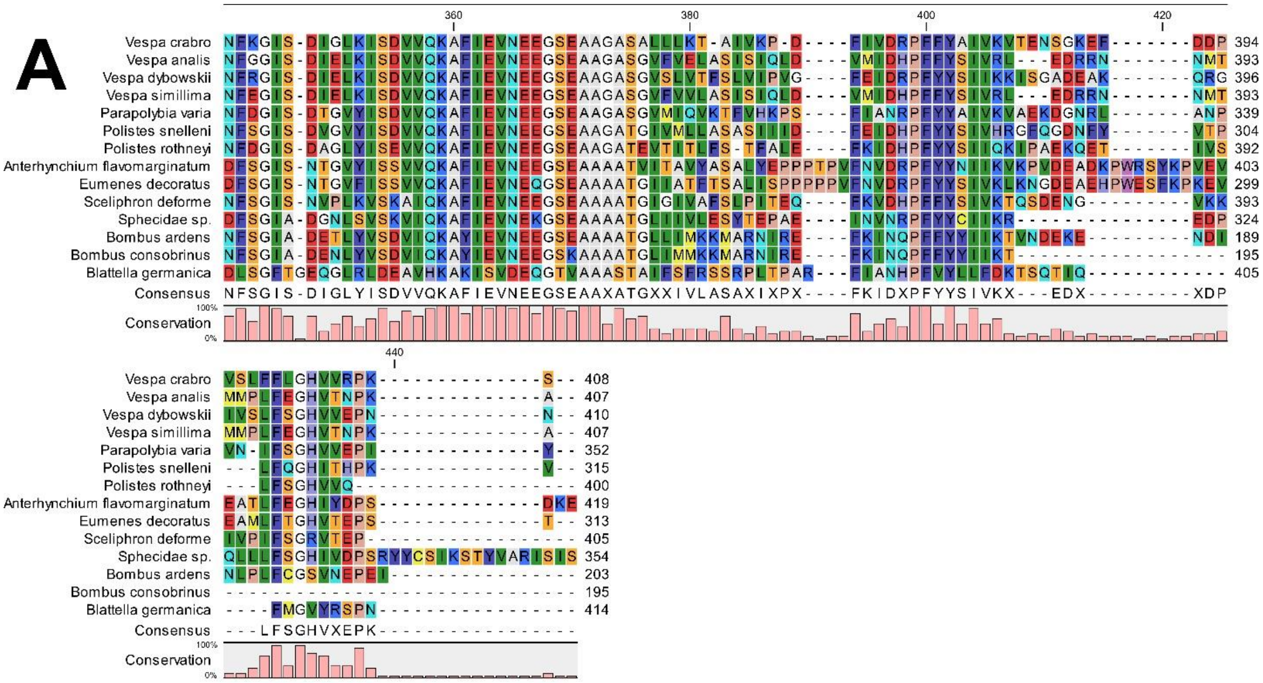

Figure S14B

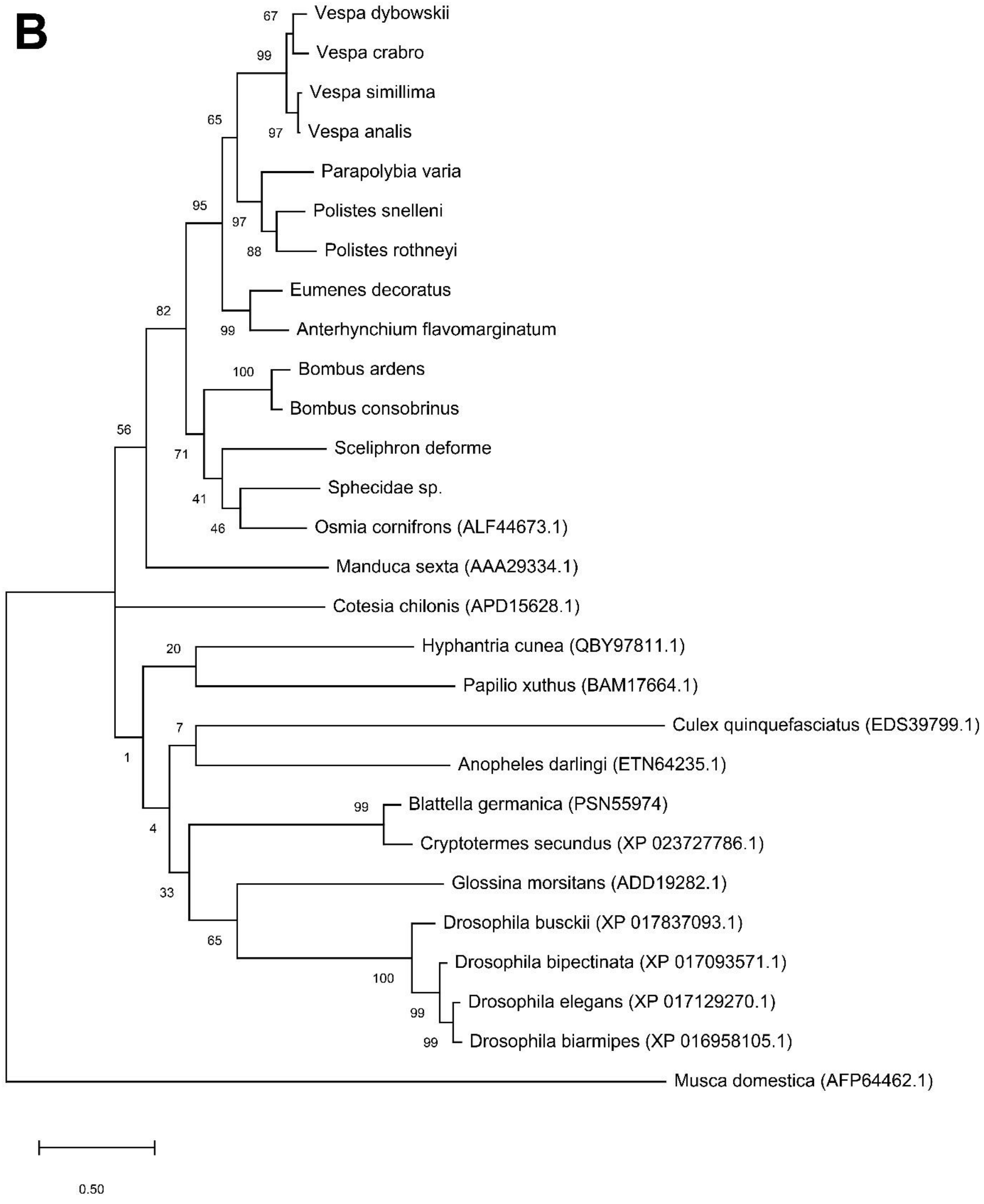

Figure S14. Amino acid alignments of serine protease inhibitor. A) Alignment of amino acid sequences from *V. crabro, V. analis, V. dybowskii, V. simillima, P. varia, P. snelleni, P. rothneyi, A. flavomarginatum, E. decoratus, S. deforme, Sphecidae* sp., *B. ardens, B. consobrinus* and *B. germanica*. B) Phylogenetic analysis of serine protease inhibitor.

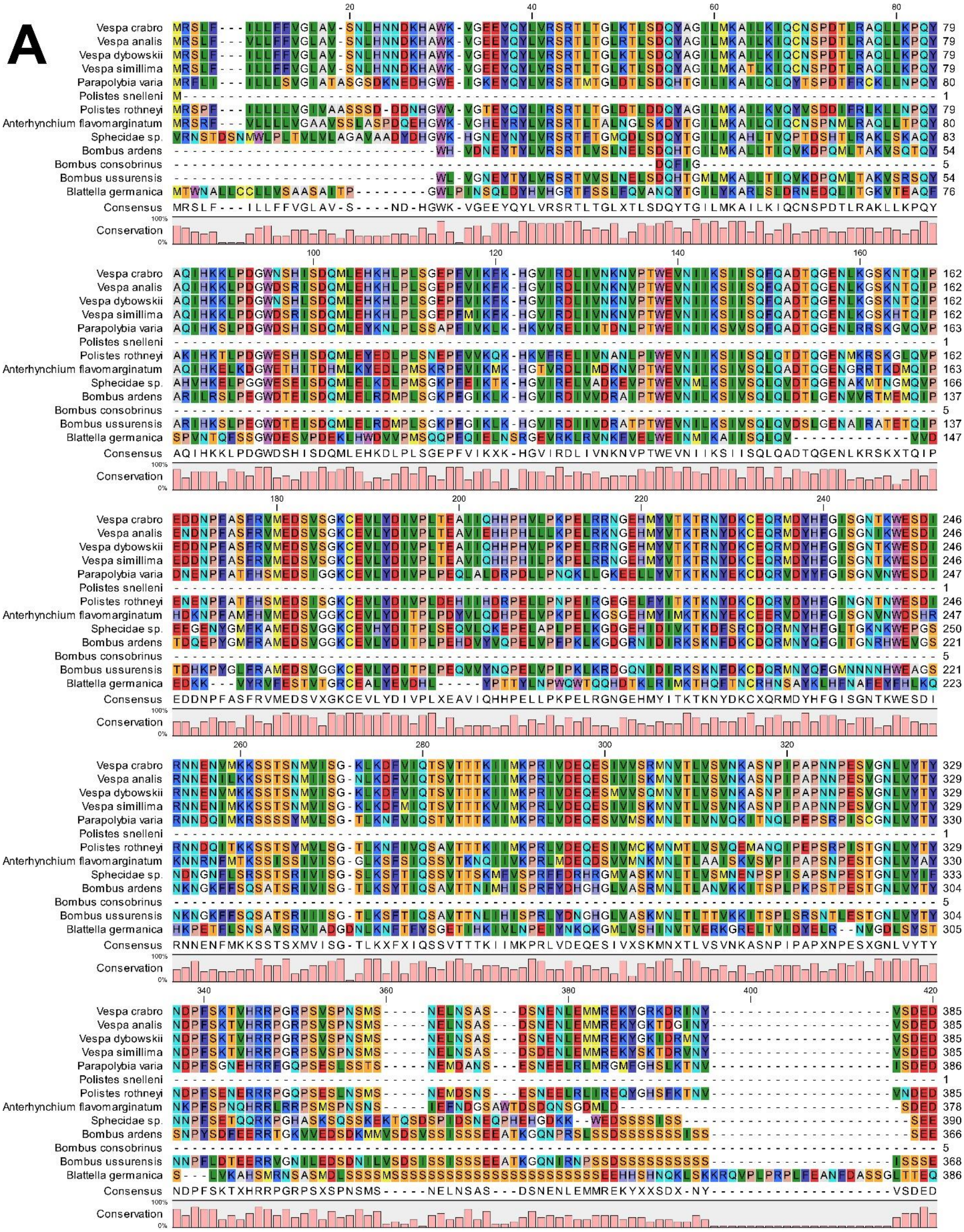
A
Vespa crabro
Vespa analis
Vespa dybowskii
Vespa simillima
Parapolybia varia
Polistes snelleni
Polistes rothneyi
Anterhynchium flavomarginatum
Sphecidae sp.
Bombus ardens
Bombus consobrinus
Bombus ussurensis
Blattella germanica
Consensus
Conservation

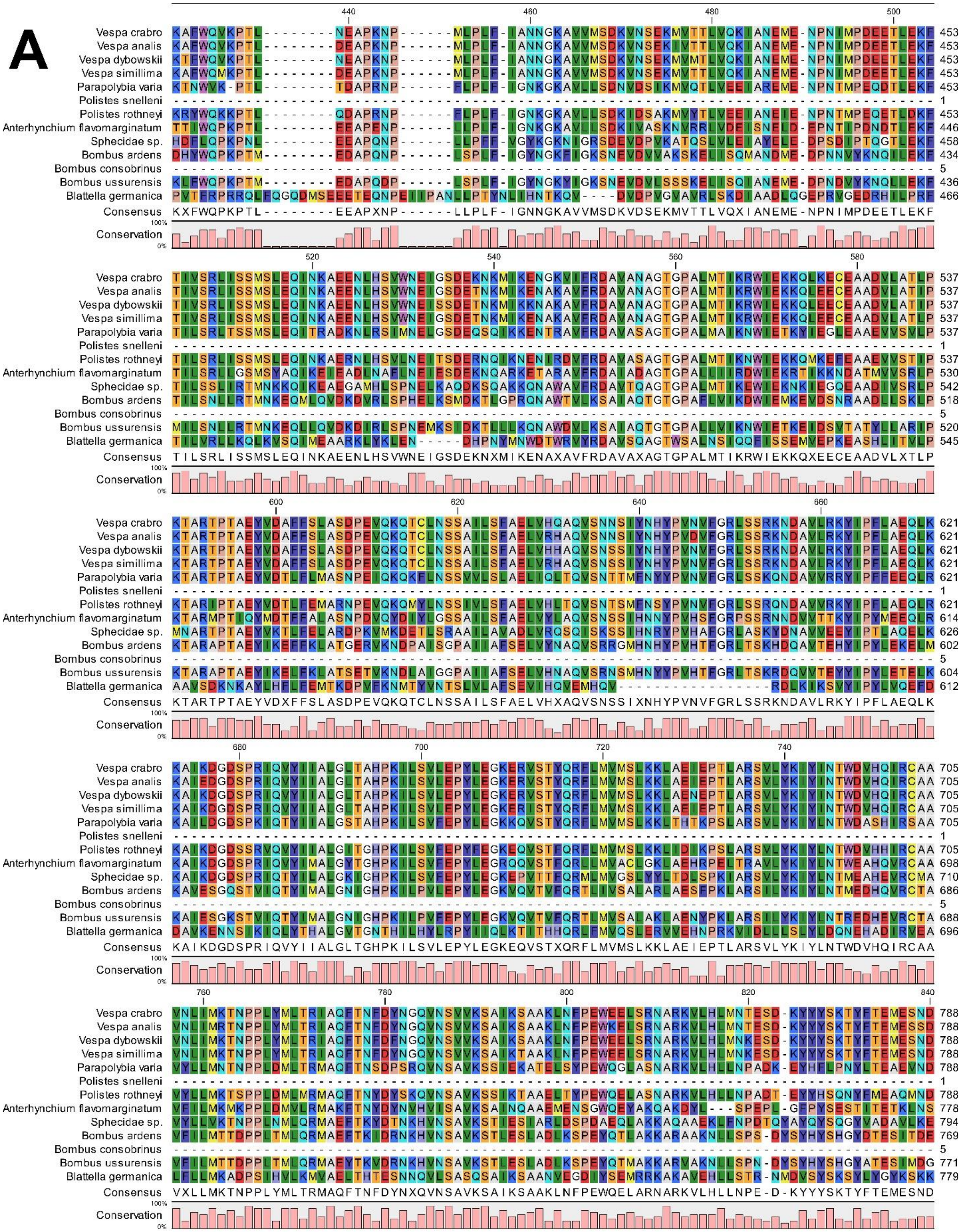

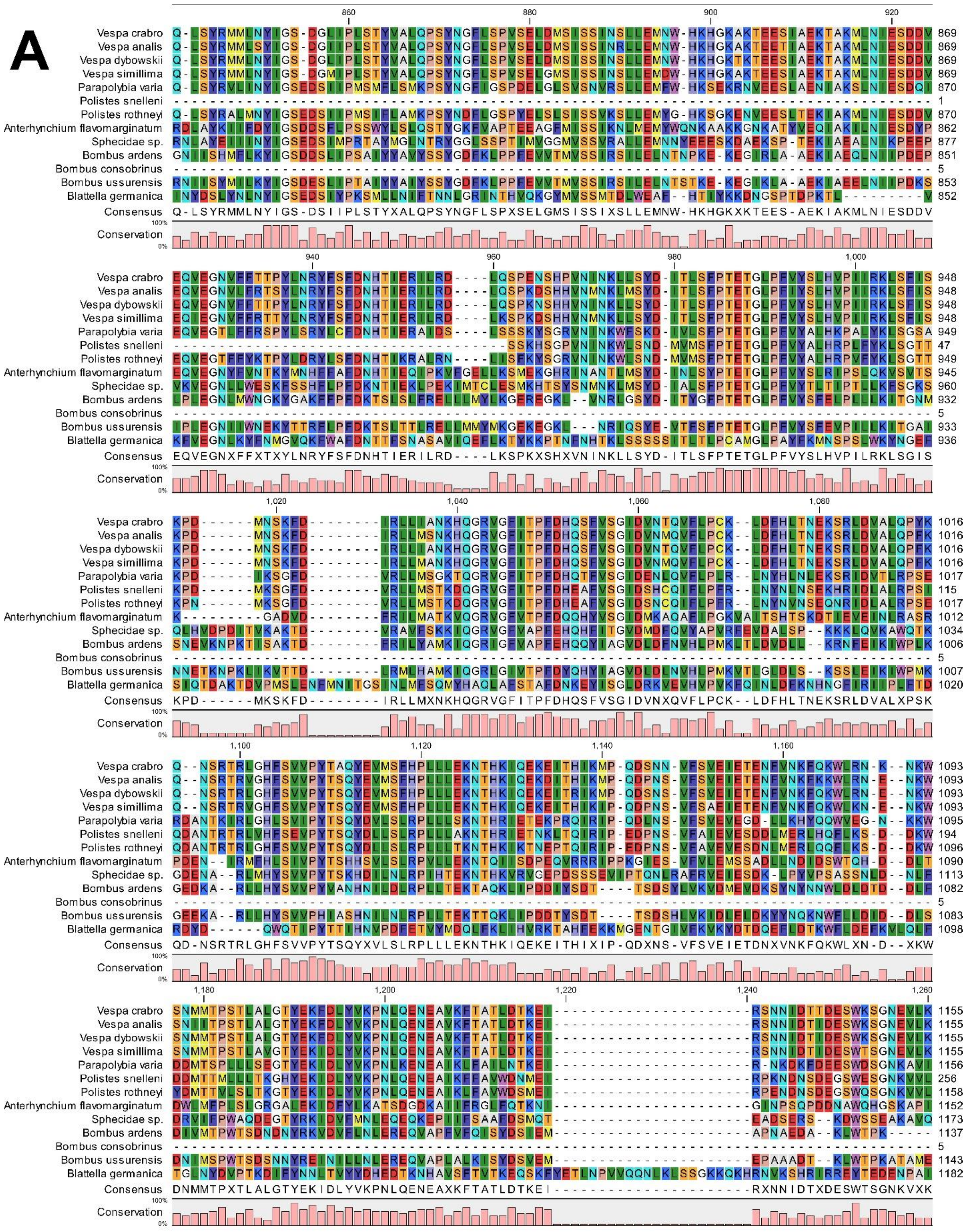
A
Vespa crabro
Vespa analis
Vespa dybowskii
Vespa simillima
Parapolybia varia
Polistes snelleni
Polistes rothneyi
Anterhynchium flavomarginatum
Sphecidae sp.
Bombus ardens
Bombus consobrinus
Bombus ussurensis
Blattella germanica
Consensus
Conservation

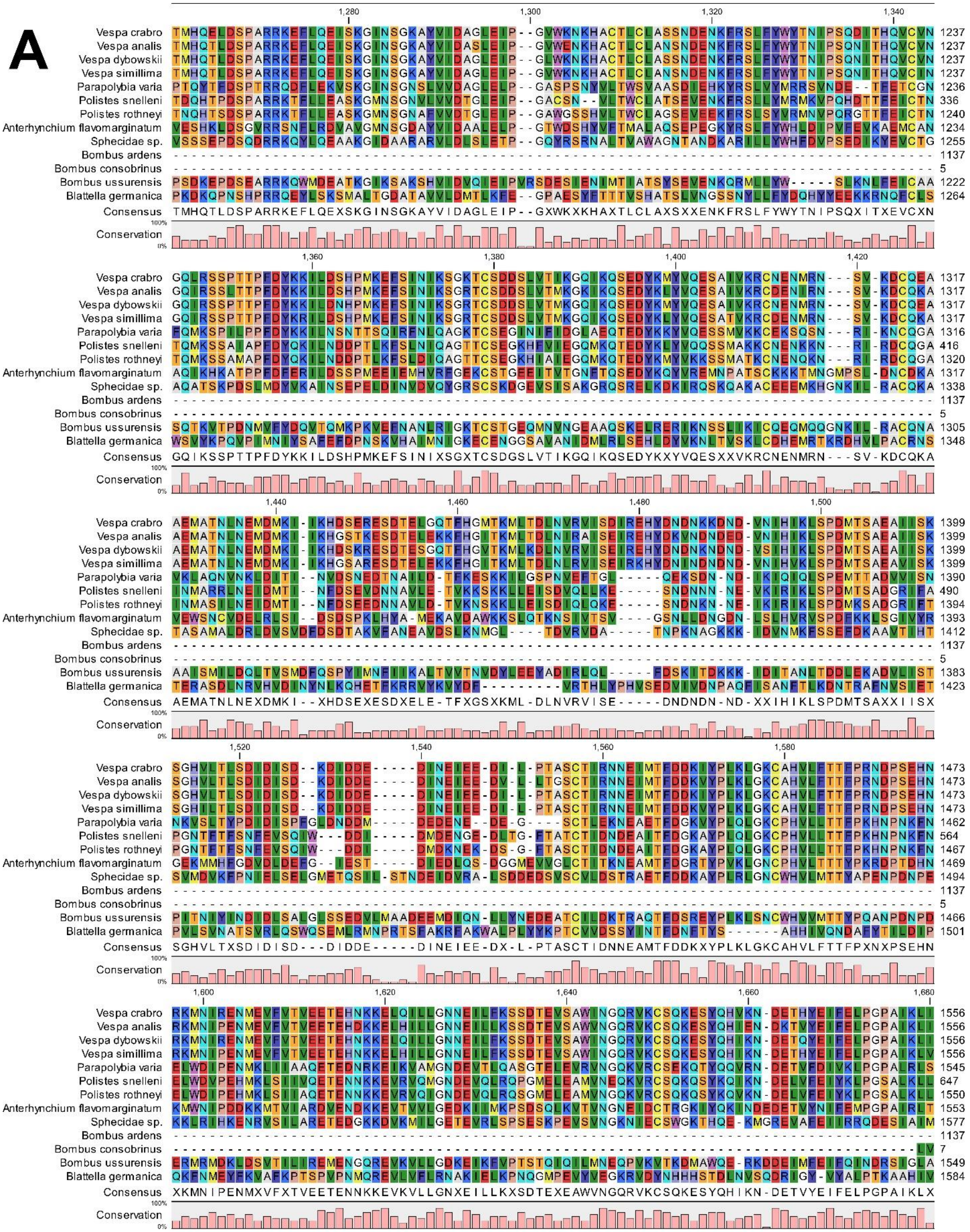
A
Vespa crabro
Vespa analis
Vespa dybowskii
Vespa simillima
Parapolybia varia
Polistes snelleni
Polistes rothneyi
Anterhynchium flavomarginatum
Sphecidae sp.
Bombus ardens
Bombus consobrinus
Bombus ussurensis
Blattella germanica
Consensus
Conservation

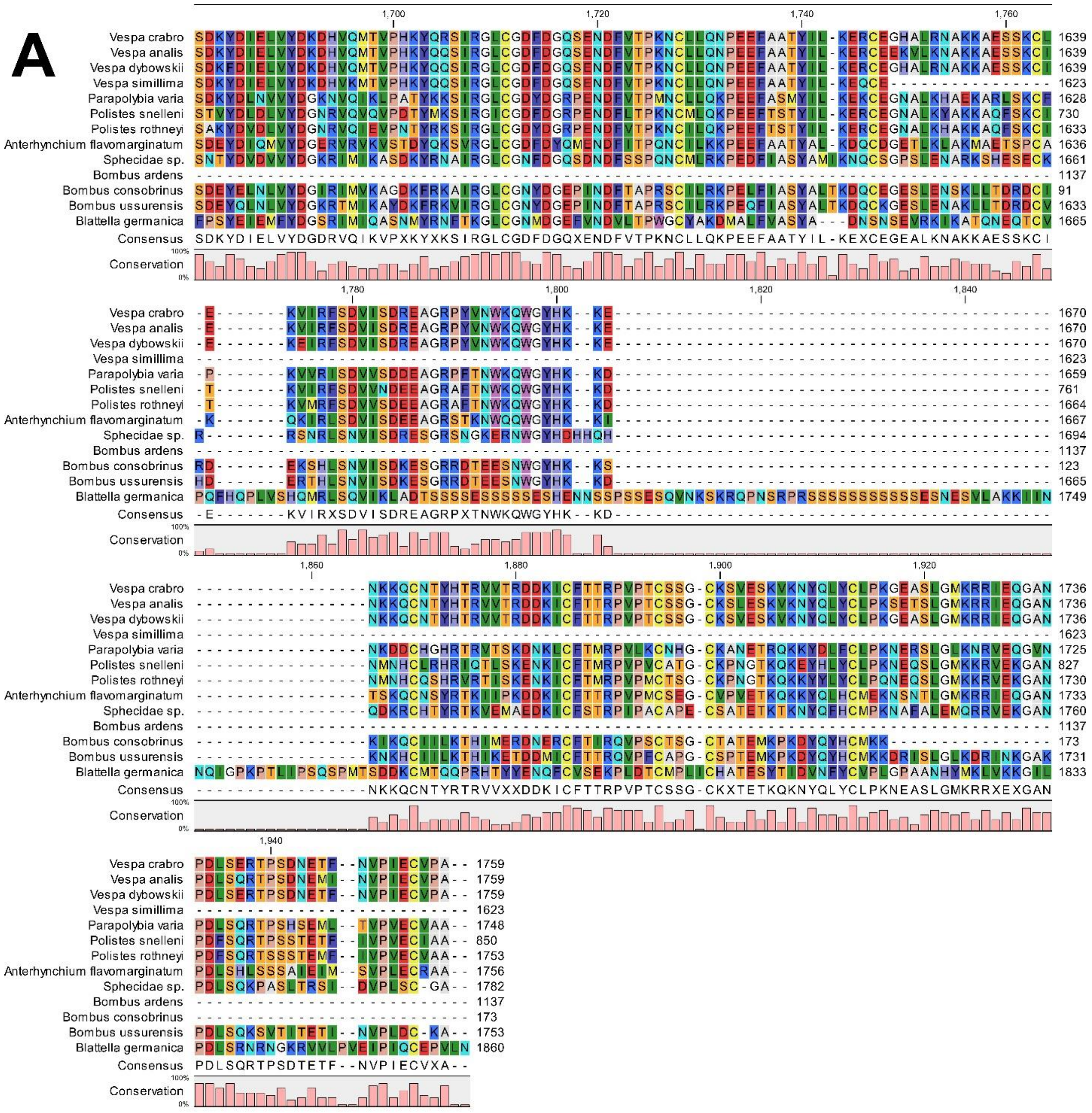

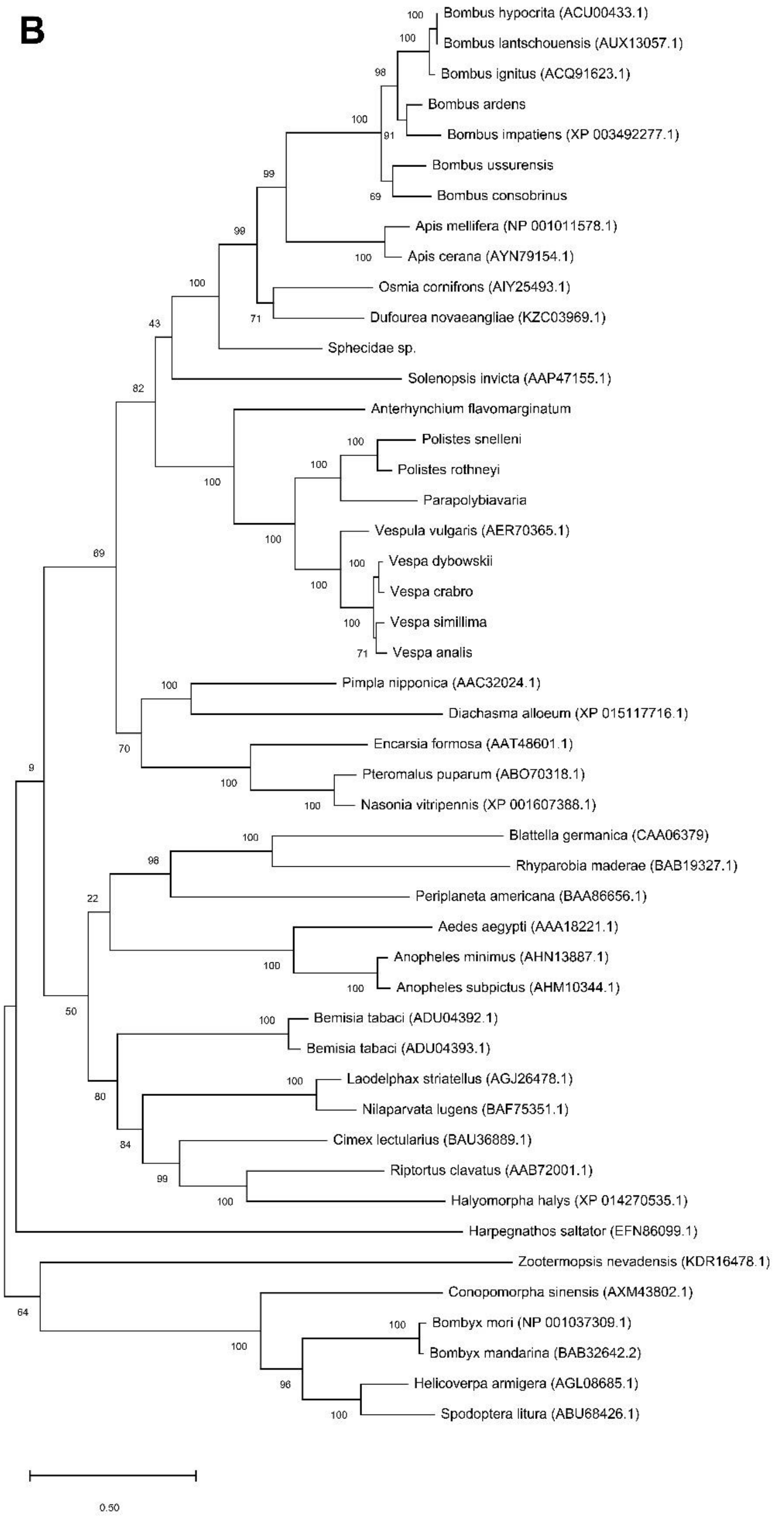

Figure S15. Amino acid alignments of vitellogenin. A) Alignment of amino acid sequences from V. crabro, V. analis, V. dybowskii, V. simillima, P. varia, P. snelleni, P. rothneyi, A. flavomarginatum, Sphecidae sp., B. ardens, B. consobrinus, B. ussurensis and B. germanica. B) Phylogenetic analysis of vitellogenin.

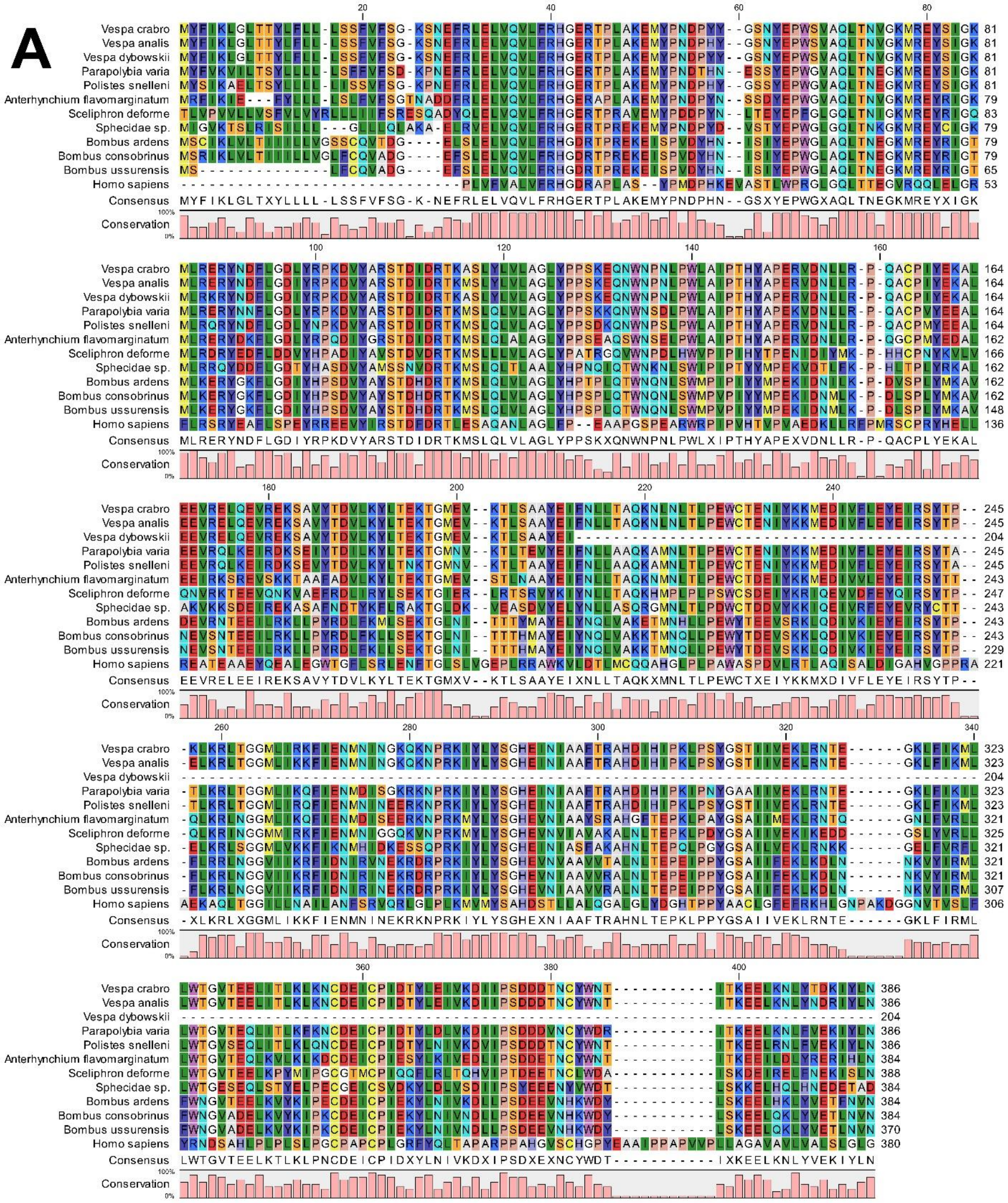
A
Vespa crabro
Vespa analis
Vespa dybowskii
Parapolybia varia
Polistes snelleni
Anterhynchium flavomarginatum
Sceliphron deforme
Sphecidae sp.
Bombus ardens
Bombus consobrinus
Bombus ussurensis
Homo sapiens
Consensus
Conservation

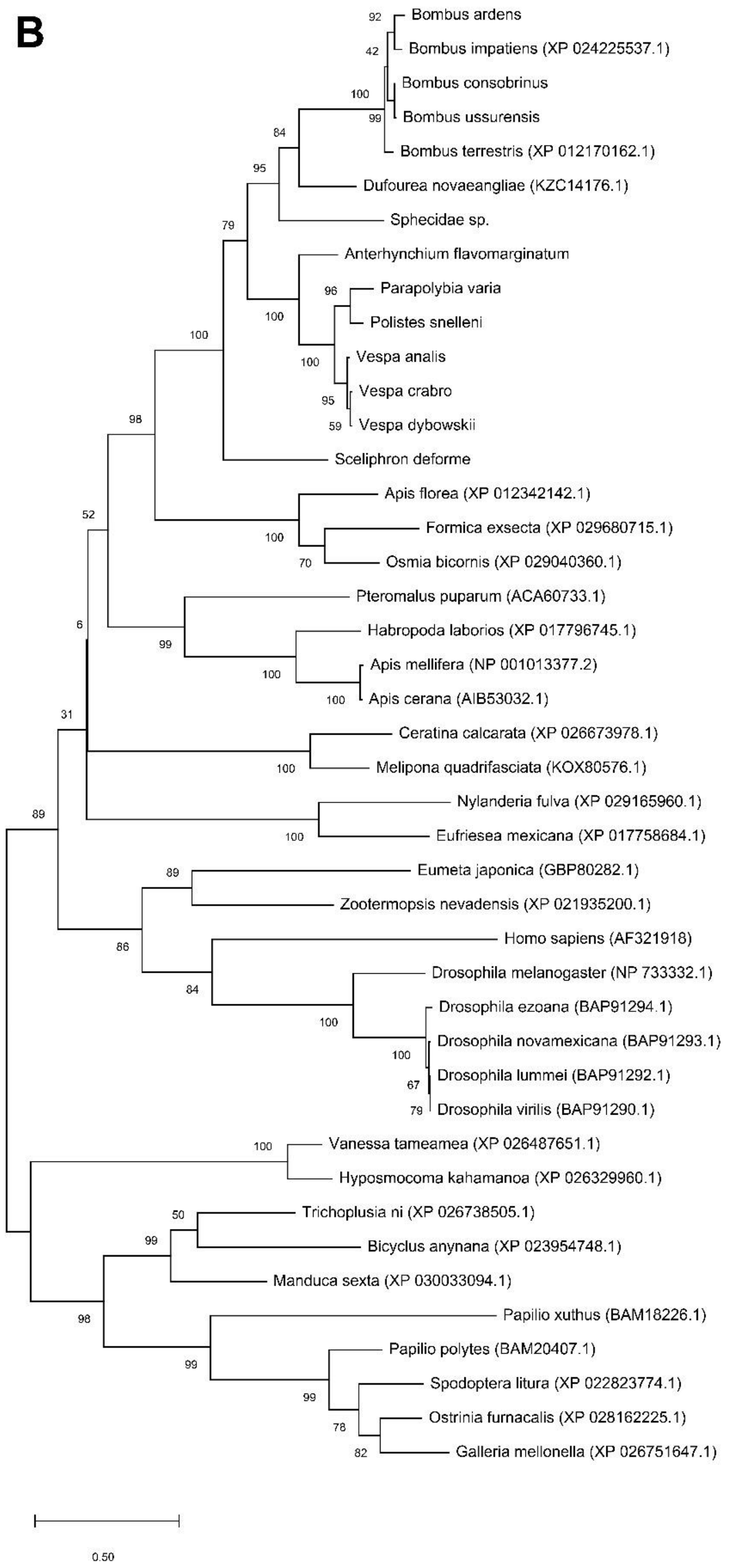

Figure S16. Amino acid alignments of acid phosphatase. A) Alignment of amino acid sequences from V. crabro, V. analis, V. dybowskii, P. varia, P. snelleni, A. flavomarginatum, S. deforme, Sphecidae sp., B. ardens, B. consobrinus, B. ussurensis and H. sapiens. B) Phylogenetic analysis of acid phosphatase.

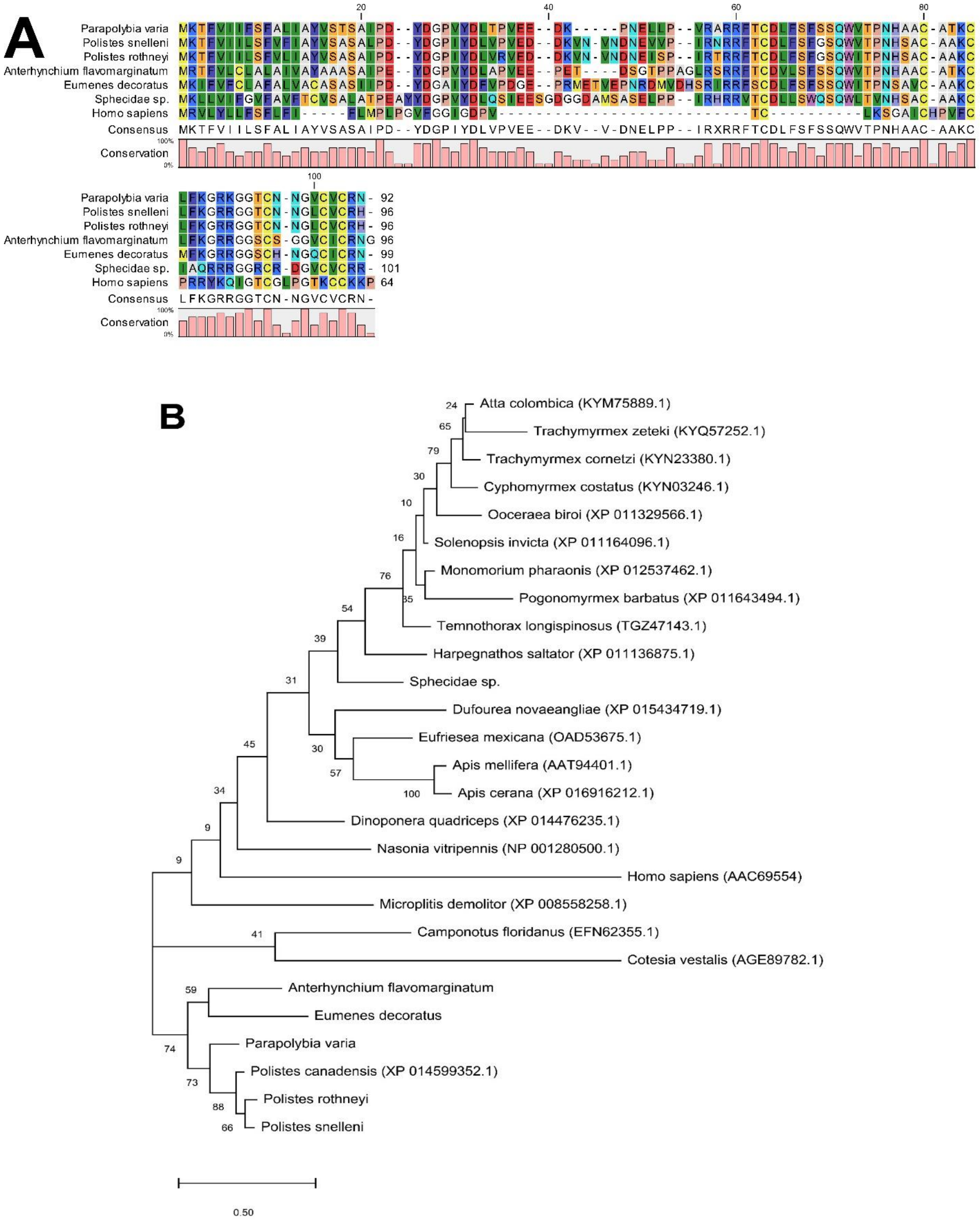

Figure S17. Amino acid alignments of defensin 2. A) Alignment of amino acid sequences from *P. varia*, *P. snelleni*, *P. rothneyi*, *A. flavomarginatum*, *E. decoratus*, *Sphecidae* sp. and *H. sapiens*. B) Phylogenetic analysis of defensin 2.

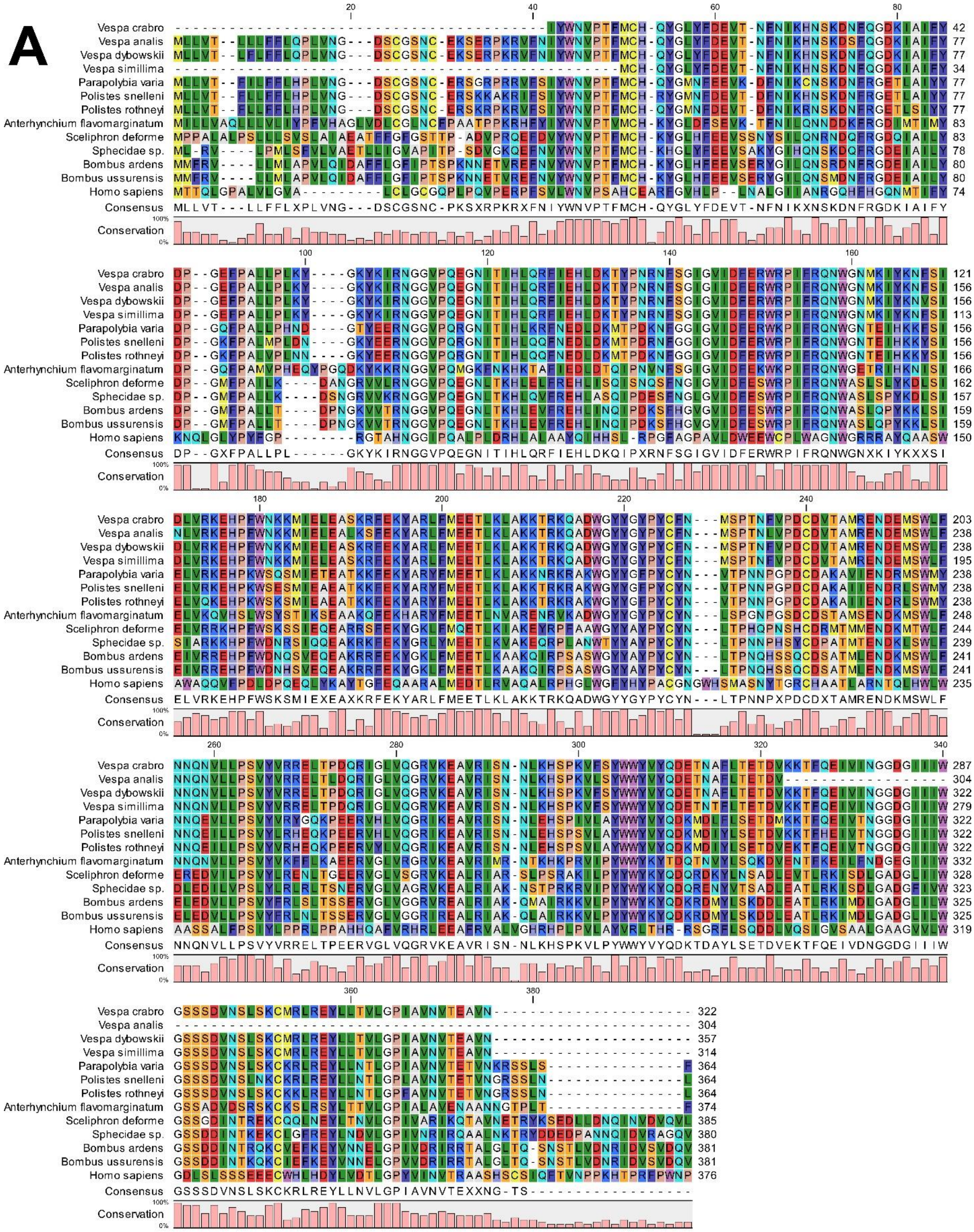
A
Vespa crabro
Vespa analis
Vespa dybowskii
Vespa simillima
Parapolybia varia
Polistes snelleni
Polistes rothneyi
Anterhynchium flavomarginatum
Sceliphron deforme
Sphecidae sp.
Bombus ardens
Bombus ussurensis
Homo sapiens
Consensus
Conservation

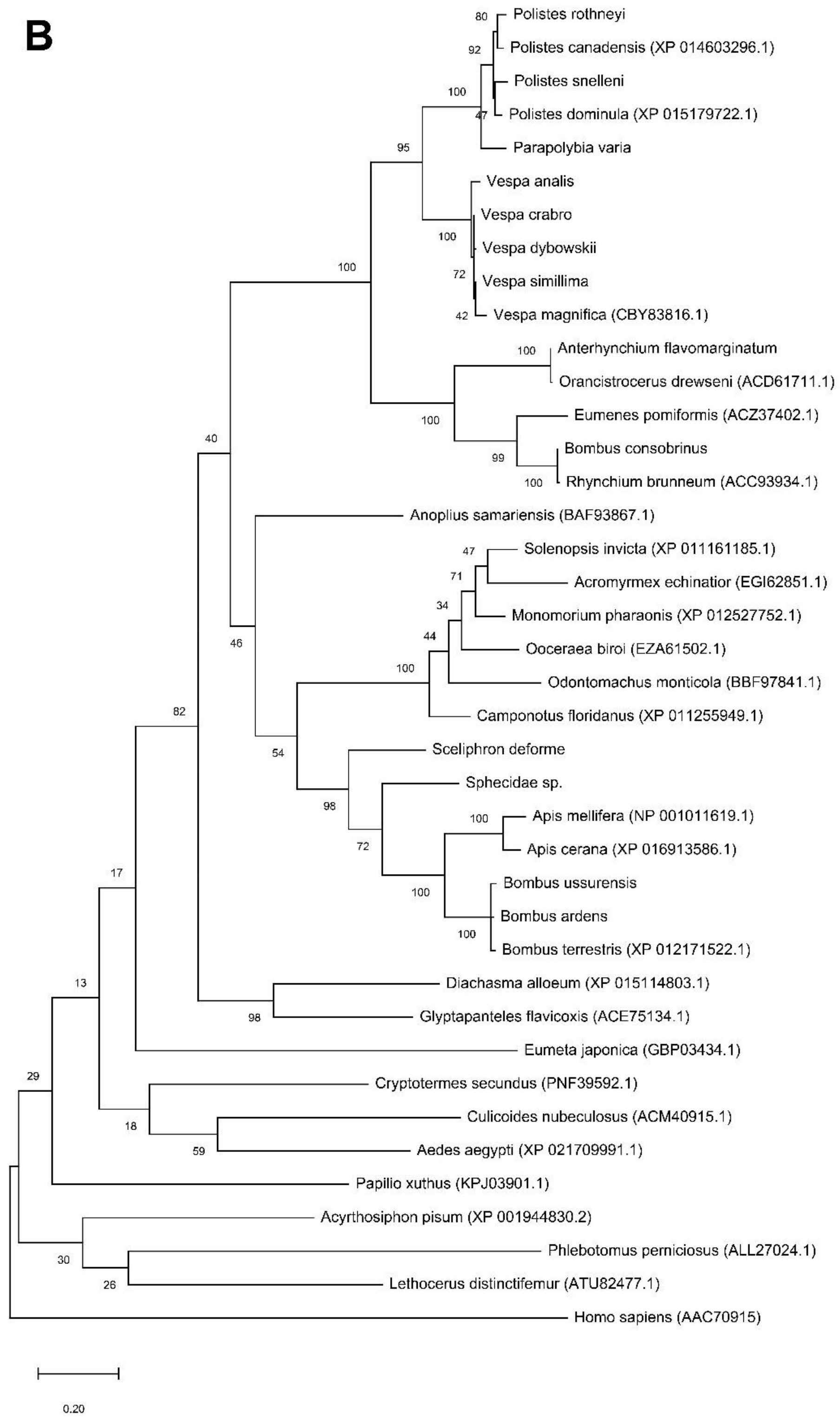

Figure S18. Amino acid alignments of hyaluronidase. A) Alignment of amino acid sequences from V. crabro, V. analis, V. dybowskii, V. simillima, P. varia, P. snelleni, P. rothneyi, A. flavomarginatum, S. deforme, Sphecidae sp., B. ardens, B. ussurensis and H. sapiens. B) Phylogenetic analysis of hyaluronidase.

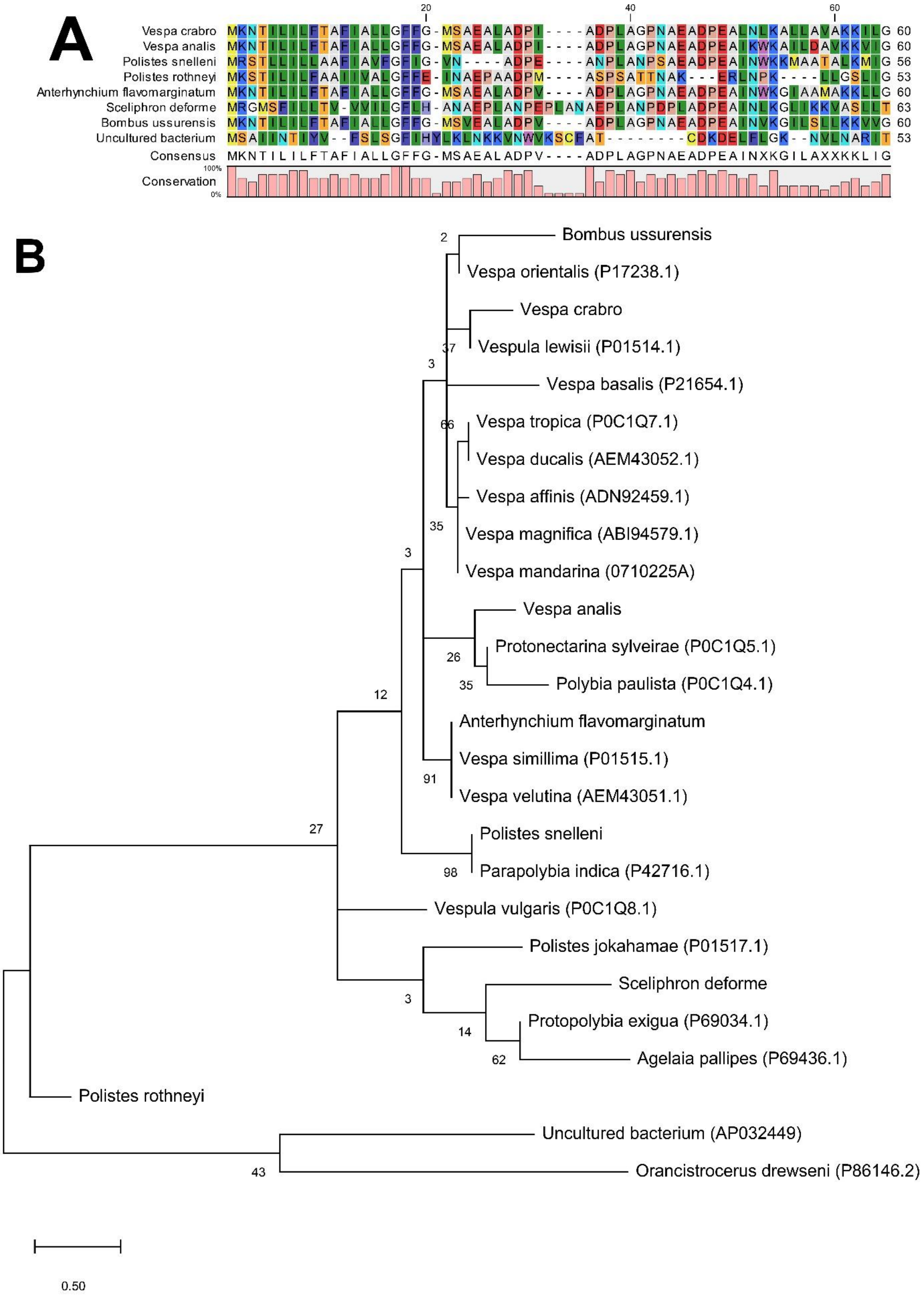
A
Vespa crabro
Vespa analis
Polistes snelleni
Polistes rothneyi
Anterhynchium flavomarginatum
Sceliphron deforme
Bombus ussurensis
Uncultured bacterium
Consensus
Conservation
B
Bombus ussurensis
Vespa orientalis (P17238.1)
Vespa crabro
Vespula lewisii (P01514.1)
Vespa basalis (P21654.1)
Vespa tropica (P0C1Q7.1)
Vespa ducalis (AEM43052.1)
Vespa affinis (ADN92459.1)
Vespa magnifica (ABI94579.1)
Vespa mandarina (0710225A)
Vespa analis
Protonectarina sylveirae (P0C1Q5.1)
Polybia paulista (P0C1Q4.1)
Anterhynchium flavomarginatum
Vespa simillima (P01515.1)
Vespa velutina (AEM43051.1)
Polistes snelleni
Parapolybia indica (P42716.1)
Vespula vulgaris (P0C1Q8.1)
Polistes jokahamae (P01517.1)
Sceliphron deforme
Protopolybia exigua (P69034.1)
Agelaia pallipes (P69436.1)
Polistes rothneyi
Uncultured bacterium (AP032449)
Orancistrocerus drewseni (P86146.2)
0.50

Figure S19. Amino acid alignments of mastoparan. A) Alignment of amino acid sequences from *V. crabro*, *V. analis*, *P. snelleni*, *P. rothneyi*, *A. flavomarginatum*, *S. deforme*, *B. ussurensis* and uncultured bacterium. B) Phylogenetic analysis of mastoparan.

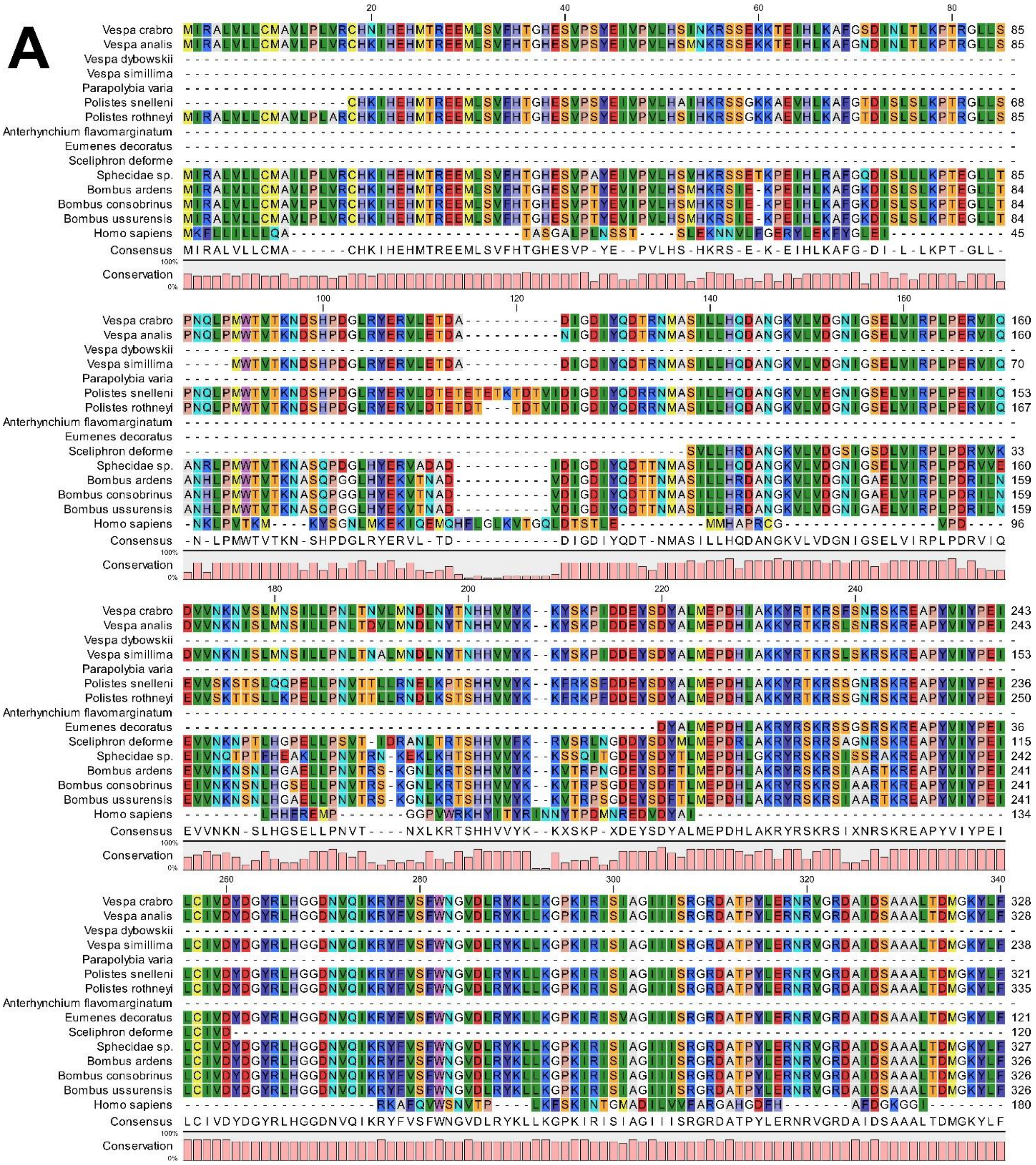

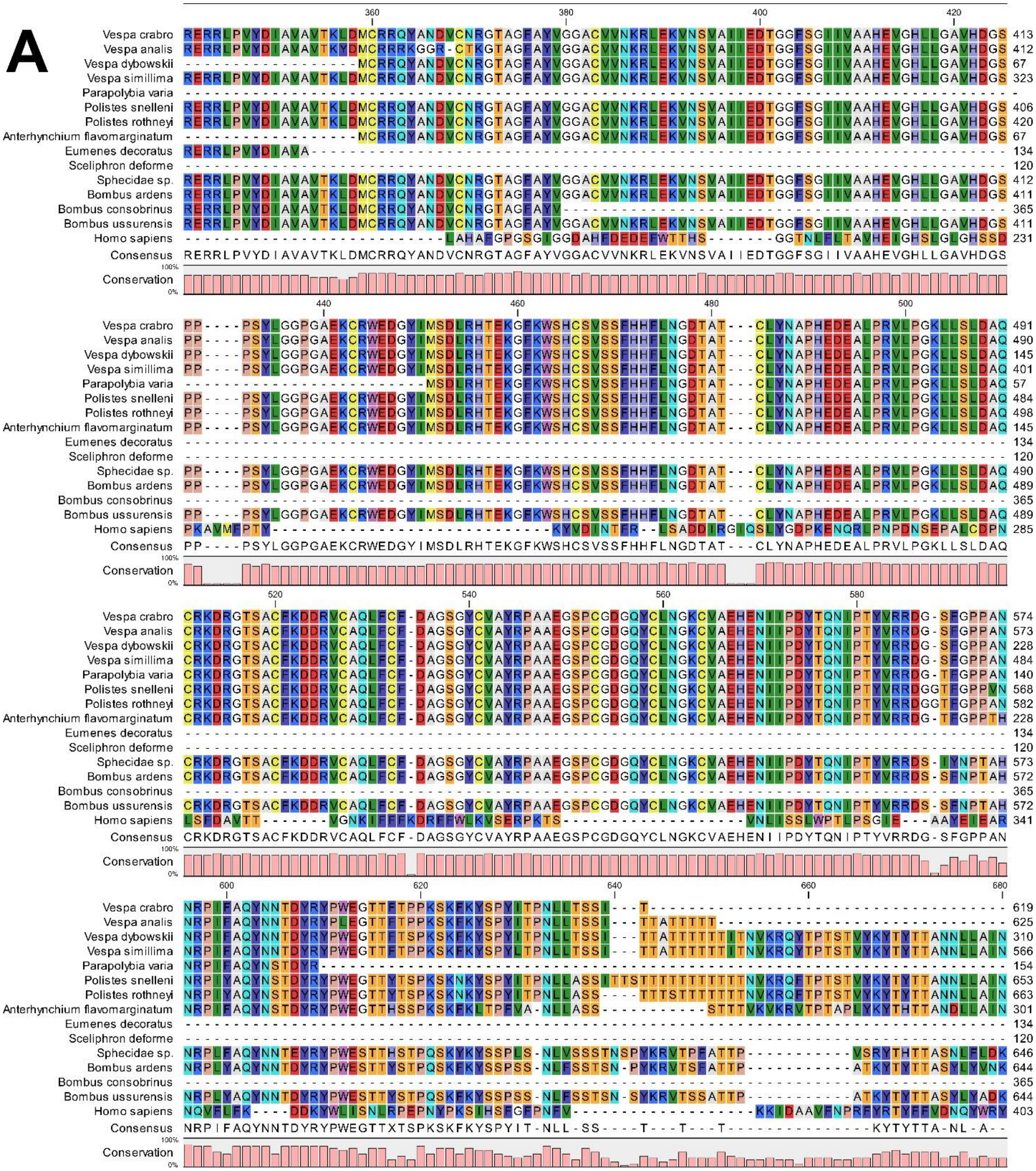
A
Vespa crabro
Vespa analis
Vespa dybowskii
Vespa simillima
Parapolybia varia
Polistes snelleni
Polistes rothneyi
Anterhynchium flavomarginatum
Eumenes decoratus
Sceliphron deforme
Sphecidae sp.
Bombus ardens
Bombus consobrinus
Bombus ussurensis
Homo sapiens
Consensus
Conservation
100%
0%

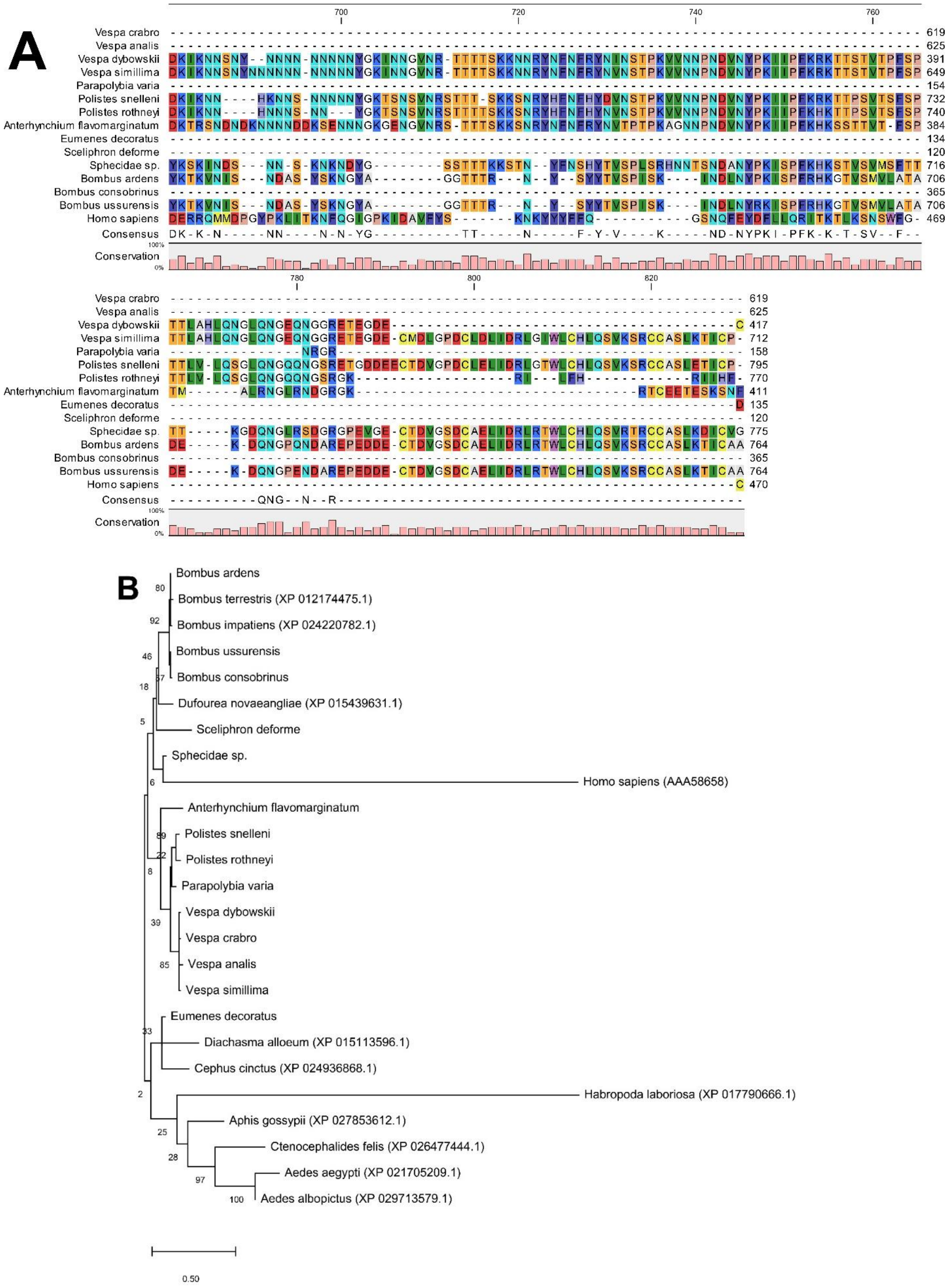
A
Vespa crabro
Vespa analis
Vespa dybowskii
Vespa simillima
Parapolybia varia
Polistes snelleni
Polistes rothneyi
Anterhynchium flavomarginatum
Eumenes decoratus
Sceliphron deforme
Sphecidae sp.
Bombus ardens
Bombus consobrinus
Bombus ussurensis
Homo sapiens
Consensus
Conservation
B
Bombus ardens
Bombus terrestris (XP 012174475.1)
Bombus impatiens (XP 024220782.1)
Bombus ussurensis
Bombus consobrinus
Dufourea novaeangliae (XP 015439631.1)
Sceliphron deforme
Sphecidae sp.
Homo sapiens (AAA58658)
Anterhynchium flavomarginatum
Polistes snelleni
Polistes rothneyi
Parapolybia varia
Vespa dybowskii
Vespa crabro
Vespa analis
Vespa simillima
Eumenes decoratus
Diachasma alloeum (XP 015113596.1)
Cephus cinctus (XP 024936868.1)
Habropoda laboriosa (XP 017790666.1)
Aphis gossypii (XP 027853612.1)
Ctenocephalides felis (XP 026477444.1)
Aedes aegypti (XP 021705209.1)
Aedes albopictus (XP 029713579.1)
0.50

Figure S20. Amino acid alignments of metalloproteinase. A) Alignment of amino acid sequences from V. crabro, V. analis, V. dybowskii, V. simillima, P. varia, P. snelleni, P. rothneyi, A. flavomarginatum, E. decoratus, S. deforme, Sphecidae sp., B. ardens, B. ussurensis and H. sapiens. B) Phylogenetic analysis of metalloproteinase.

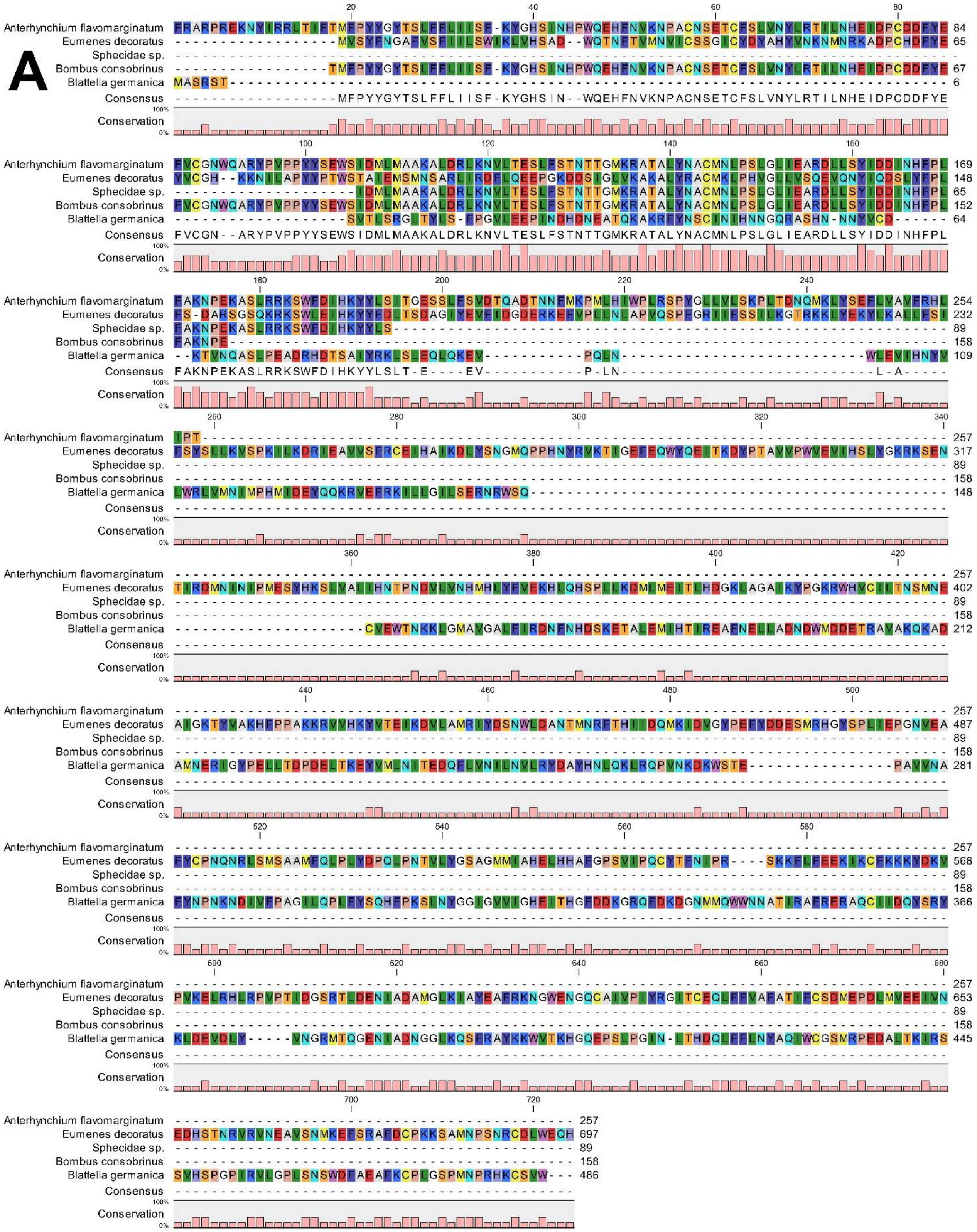
A
Anterhynchium flavomarginatum
Eumenes decoratus
Sphecidae sp.
Bombus consobrinus
Blattella germanica
Consensus
Conservation
100%
0%

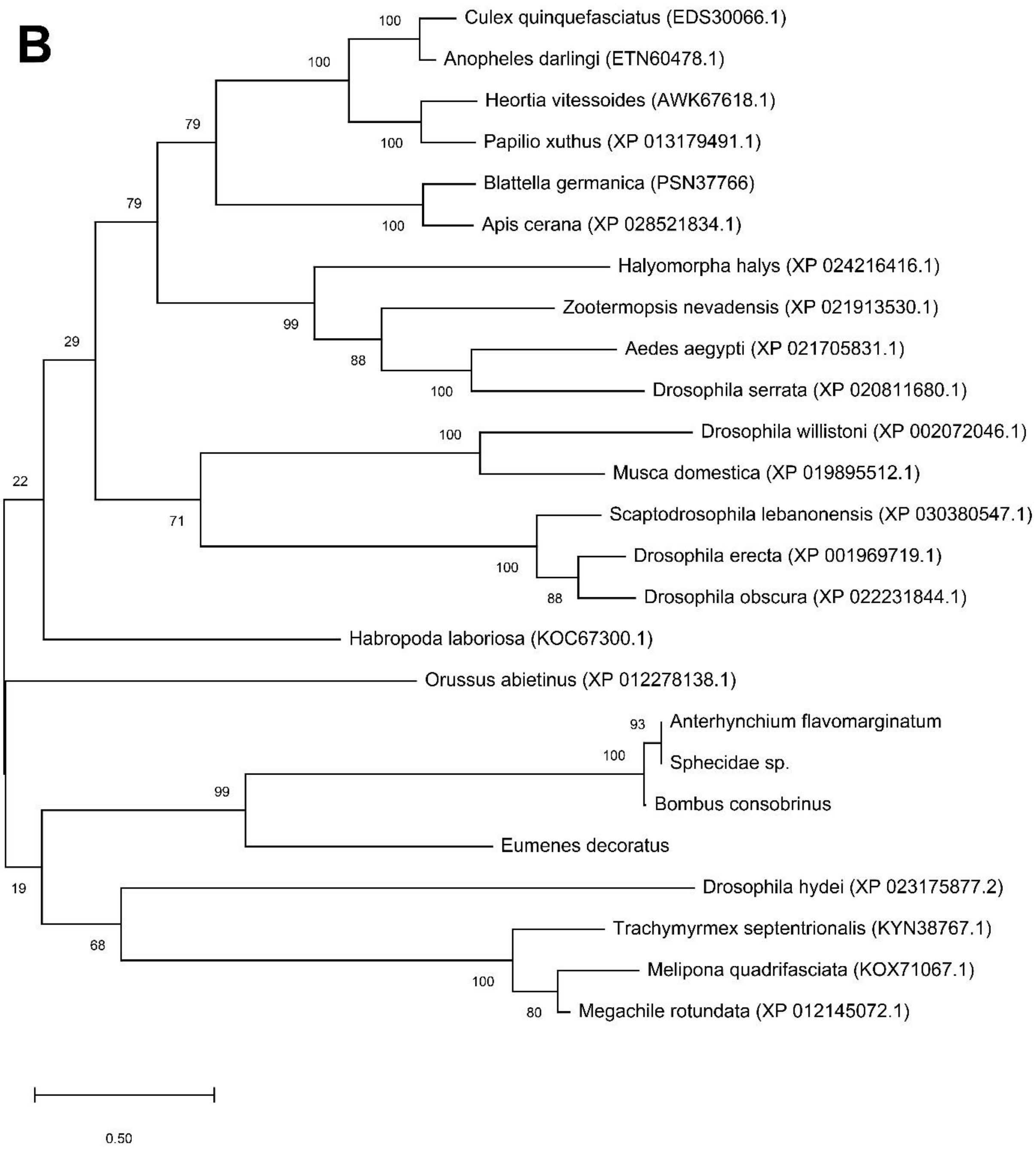

Figure S21. Amino acid alignments of neprilysin. A) Alignment of amino acid sequences from *A. flavomarginatum*, *E. decoratus*, *B. consobrinus* and *B. germanica*. B) Phylogenetic analysis of neprilysin.

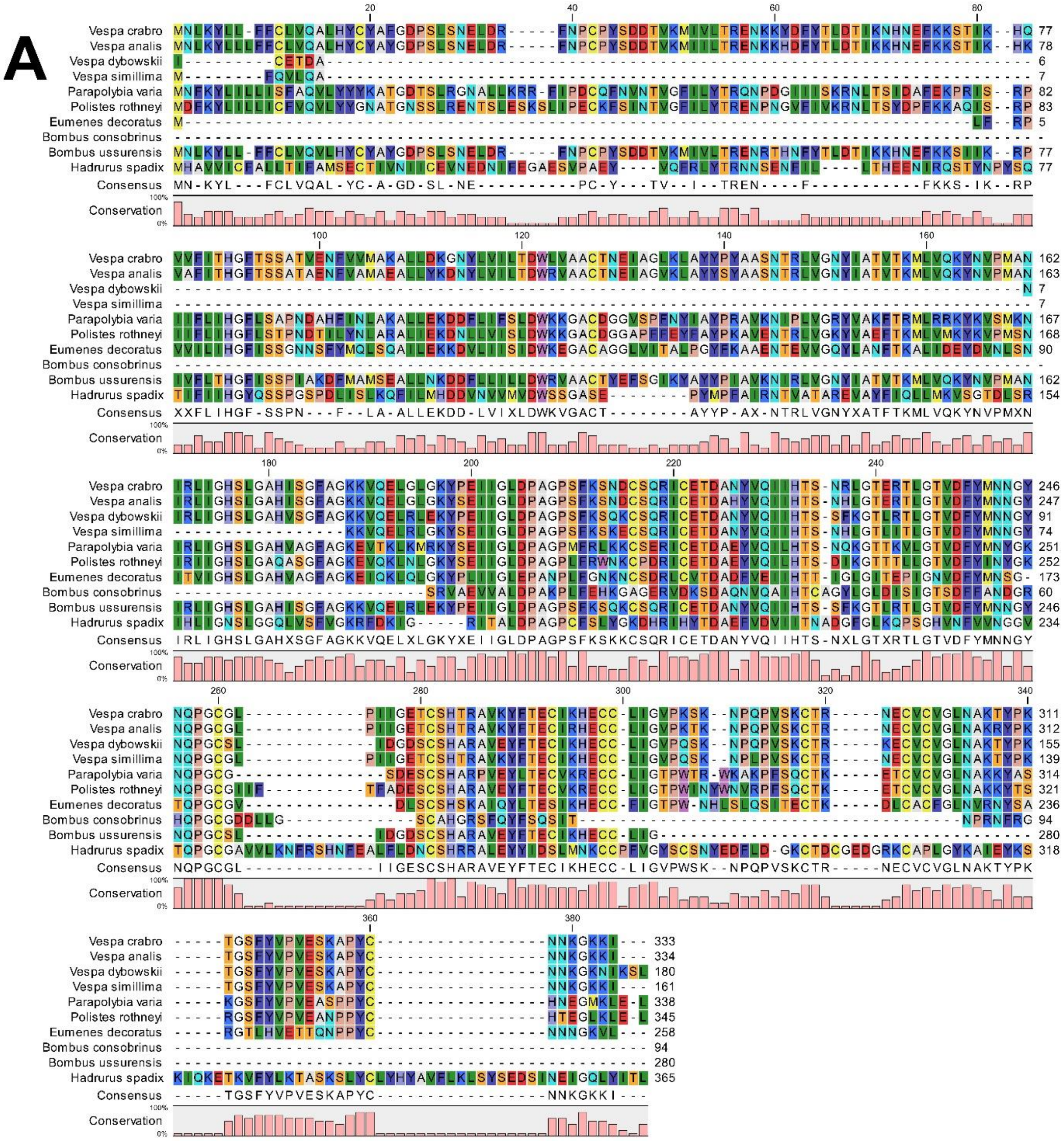

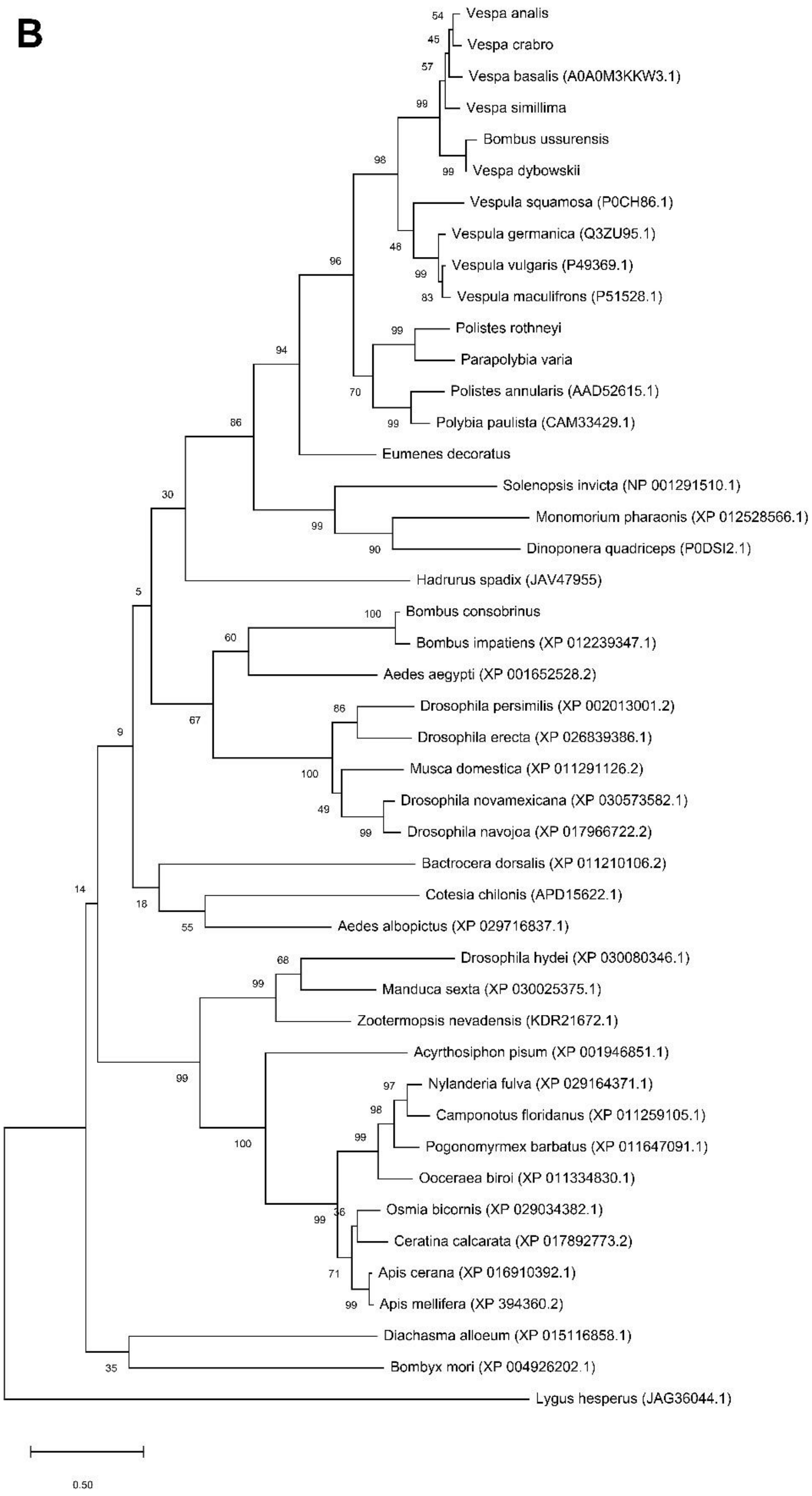

Figure S22. Amino acid alignments of phospholipase A1. A) Alignment of amino acid sequences from *V. crabro, V. analis, V. dybowskii, V. simillima, P. varia, P. rothneyi, E. decoratus, B. consobrinus, B. ussurensis* and *H. spadix*. B) Phylogenetic analysis of phospholipase A1.

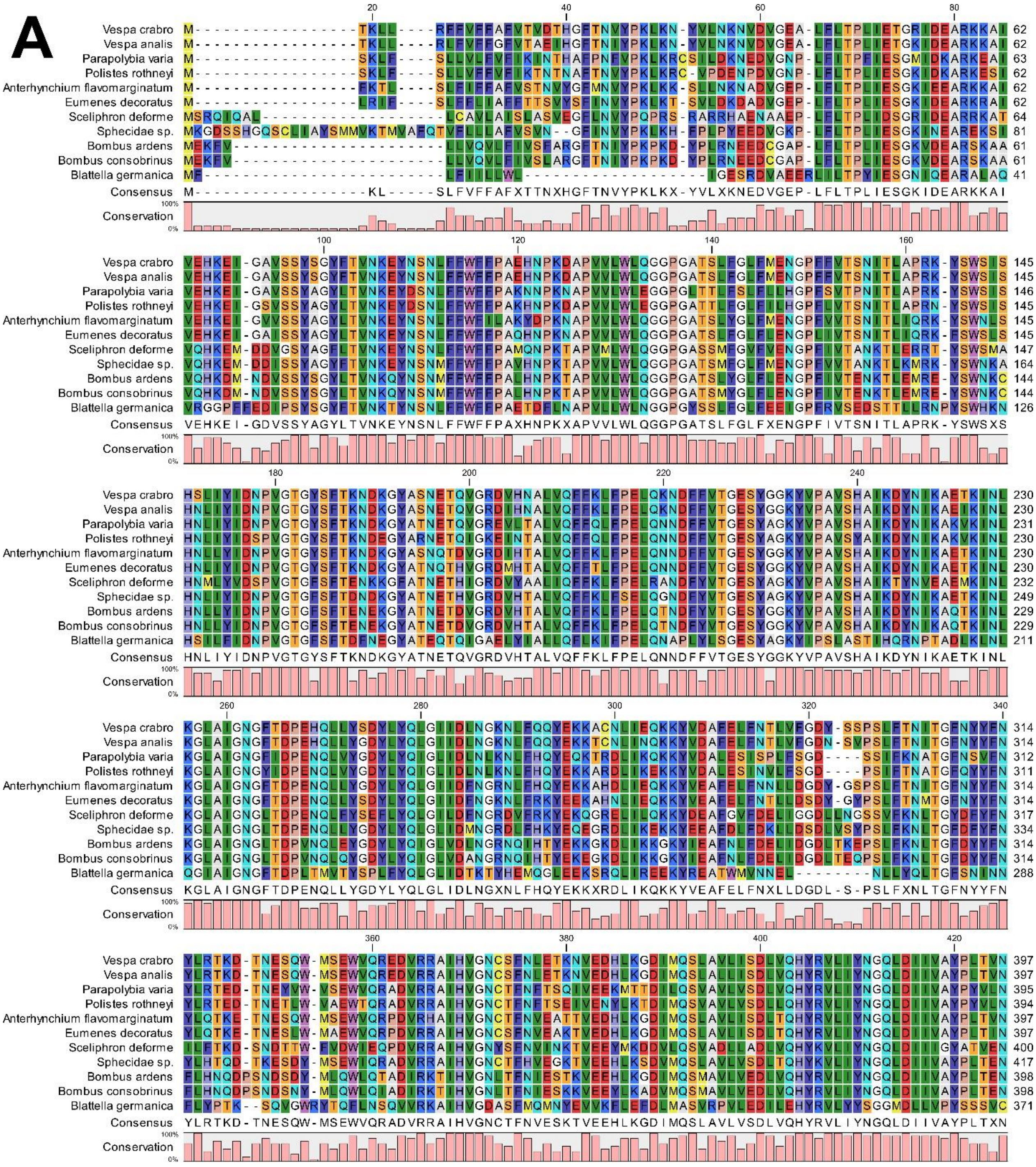

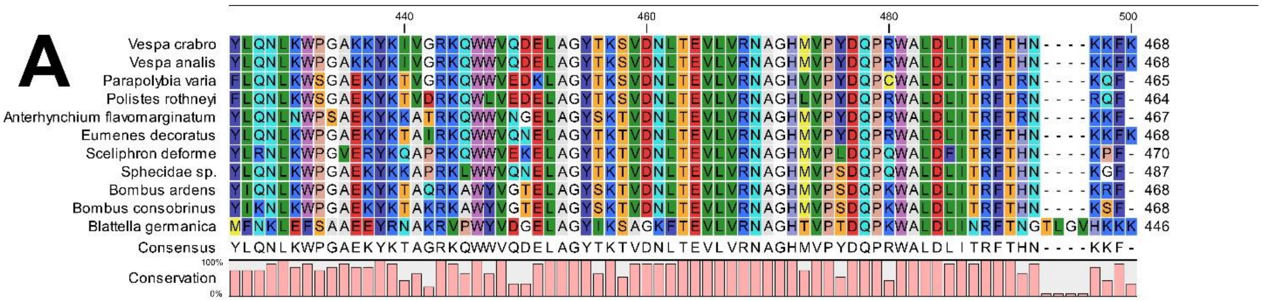

A
Vespa crabro
Vespa analis
Parapolybia varia
Polistes rothneyi
Anterhynchium flavomarginatum
Eumenes decoratus
Sceliphron deforme
Sphecidae sp.
Bombus ardens
Bombus consobrinus
Blattella germanica
Consensus
Conservation

Figure S23B

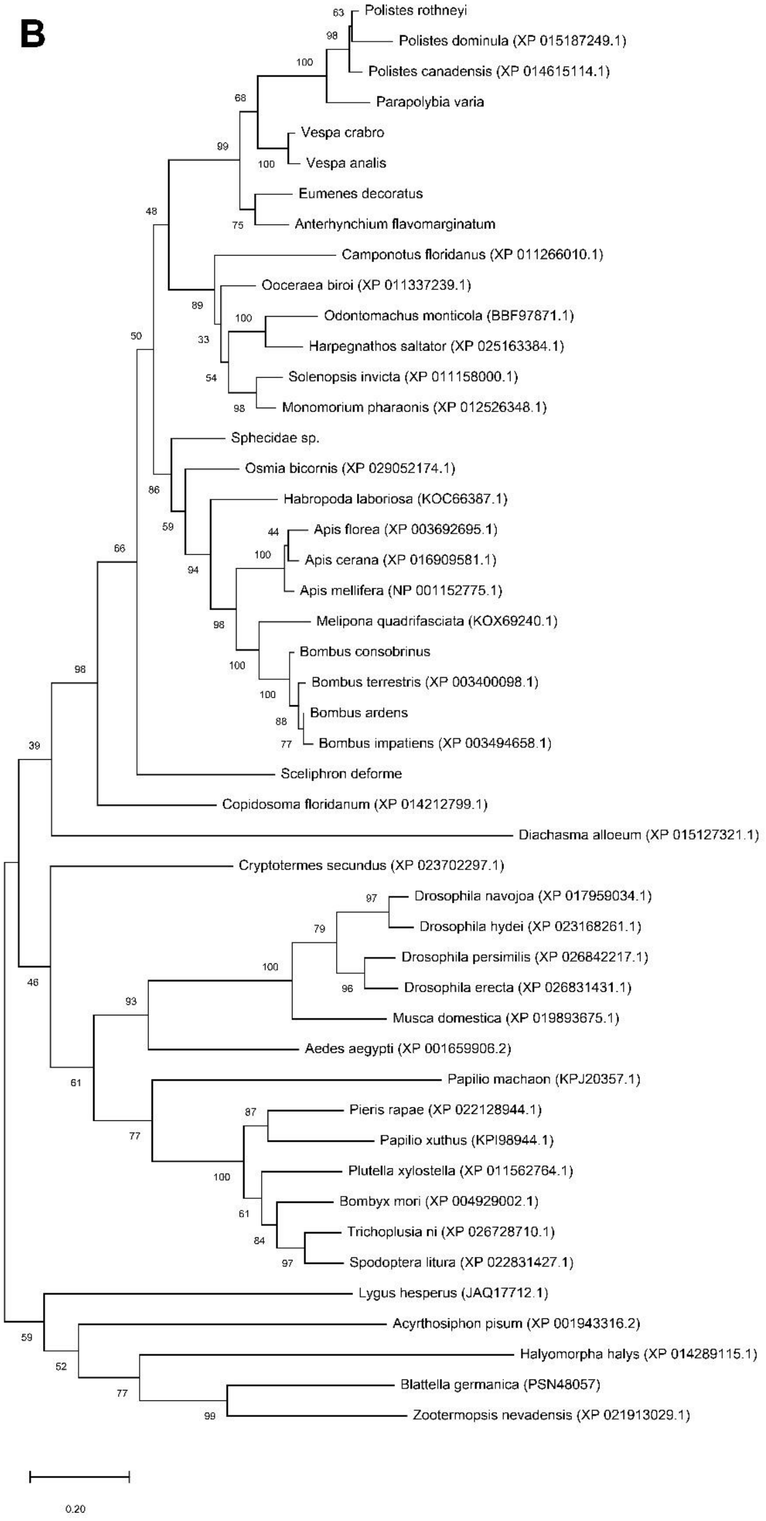

Figure S23. Amino acid alignments of serine carboxypeptidase. A) Alignment of amino acid sequences from *V. crabro*, *V. analis*, *P. varia*, *P. rothneyi*, *A. flavomarginatum*, *E. decoratus*, *S. deforme*, *Sphecidae* sp., *B. ardens*, *B. consobrinus* and *B. germanica*. B) Phylogenetic analysis of serine carboxypeptidase.

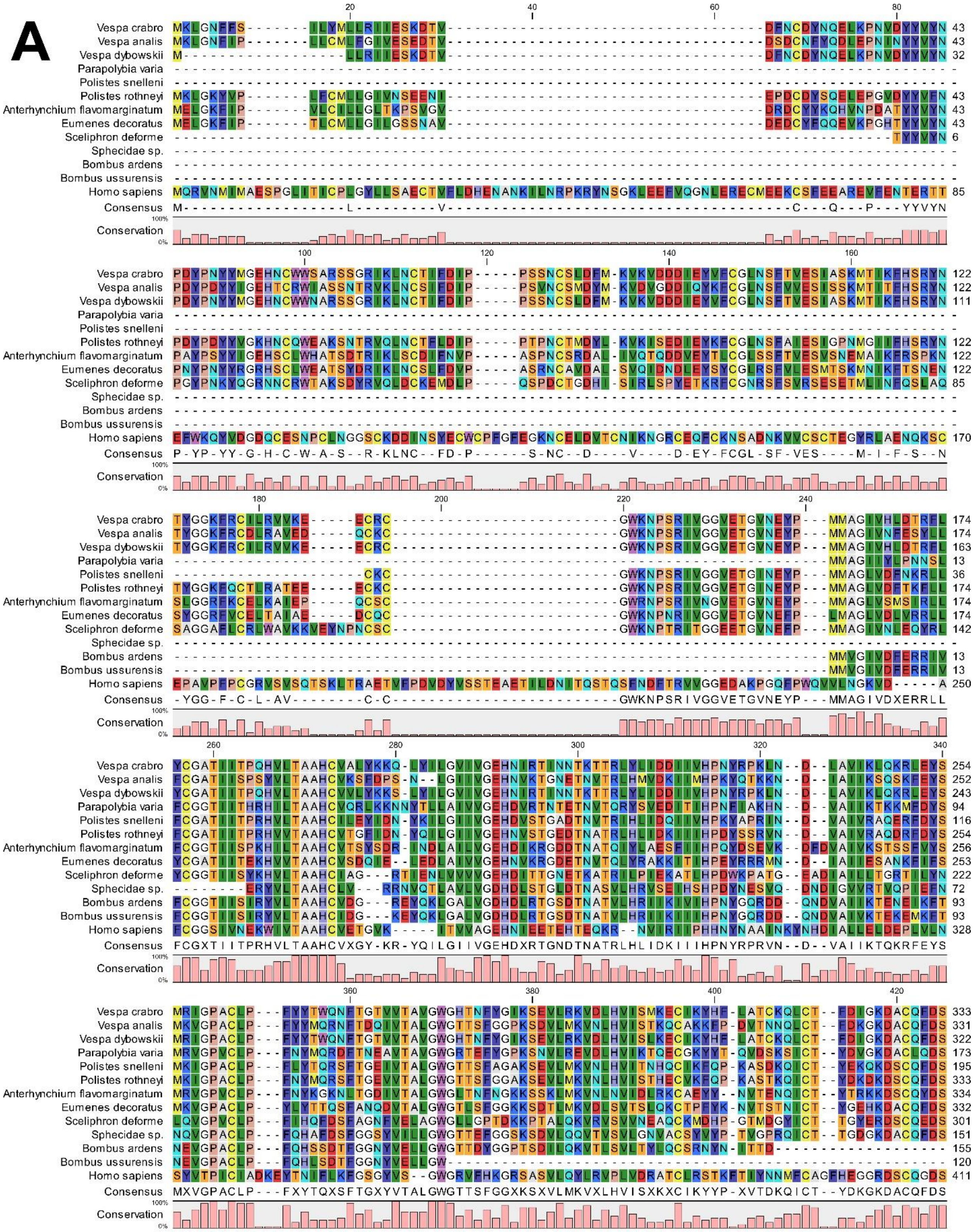
A
Vespa crabro
Vespa analis
Vespa dybowskii
Parapolybia varia
Polistes snelleni
Polistes rothneyi
Anterhynchium flavomarginatum
Eumenes decoratus
Sceliphron deforme
Sphecidae sp.
Bombus ardens
Bombus ussurensis
Homo sapiens
Consensus
Conservation

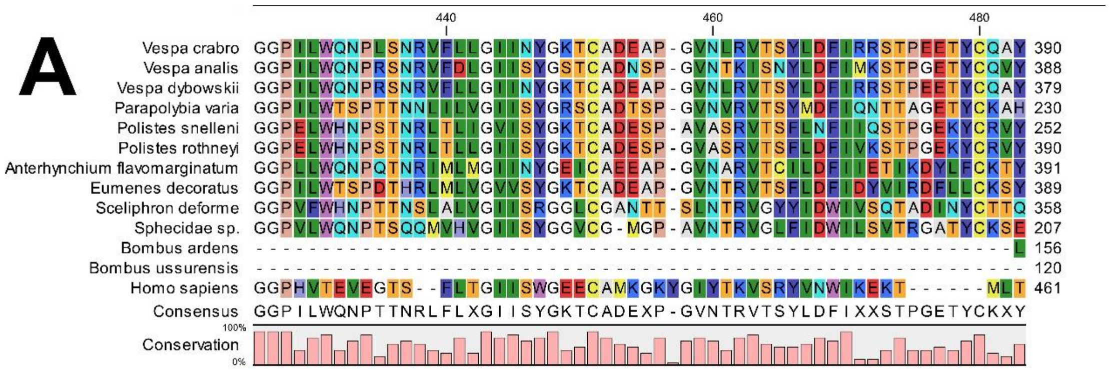
A
440
460
480
Vespa crabro GGPILWQNPLSNRVFLLGIINYGKTCADEAP-GVNLRVTSYLDFIRRSTPEETYCQAY 390
Vespa analis GGPILWQNPRSNRVFDLGIISYGSTCADNSP-GVNTKISNYLDFIMKSTPGETYCQVY 388
Vespa dybowskii GGPILWQNPRSNRVFLLGIINYGKTCADEAP-GVNLRVTSYLDFIRRSTPEETYCQAY 379
Parapolybia varia GGPILWTSPTTNNLILVGIISYGRSCADTSP-GVNVRVTSYMDFIQNTTAGETYCKAH 230
Polistes snelleni GGPELWHNPSTNRLTLIGVISYGKTCADESP-AVASRVTSFLNFIIQSTPGEKYCRVY 252
Polistes rothneyi GGPELWHNPSTNRLTLLGIISYGKTCADESP-GVASRVTSFLDFIVKSTPGEKYCRVY 390
Anterhynchium flavomarginatum GGPLLWQNPQTNRIMLMGIINYGEICAEEAP-GVNARVTCILDFIIETIKDYLFCKTY 391
Eumenes decoratus GGPILWTSPDTHRLMLVGVVSYGKTCADEAP-GVNTRVTSFLDFIDYVIRDFLLCKSY 389
Sceliphron deforme GGPVFWHNPTTNSLALVGIISRGGLCGANTT-SLNTRVGYYIDWIVSQTADINYCTTQ 358
Sphecidae sp. GGPVLWQNPTSQQMVHVGIISYGGVCG-MGP-AVNTRVGLFIDWILSVTRGATYCKSE 207
Bombus ardens --L 156
Bombus ussurensis --- 120
Homo sapiens GGPHVTEVEGTS--FLTGIISWGEECAMKGKYGIYTKVSRYVNWIKEKT------MLT 461
Consensus GGPILWQNPTTNRLFLXGIISYGKTCADEXP-GVNTRVTSYLDFIXXSTPGETYCKXY
100%
Conservation
0%

Figure S24B

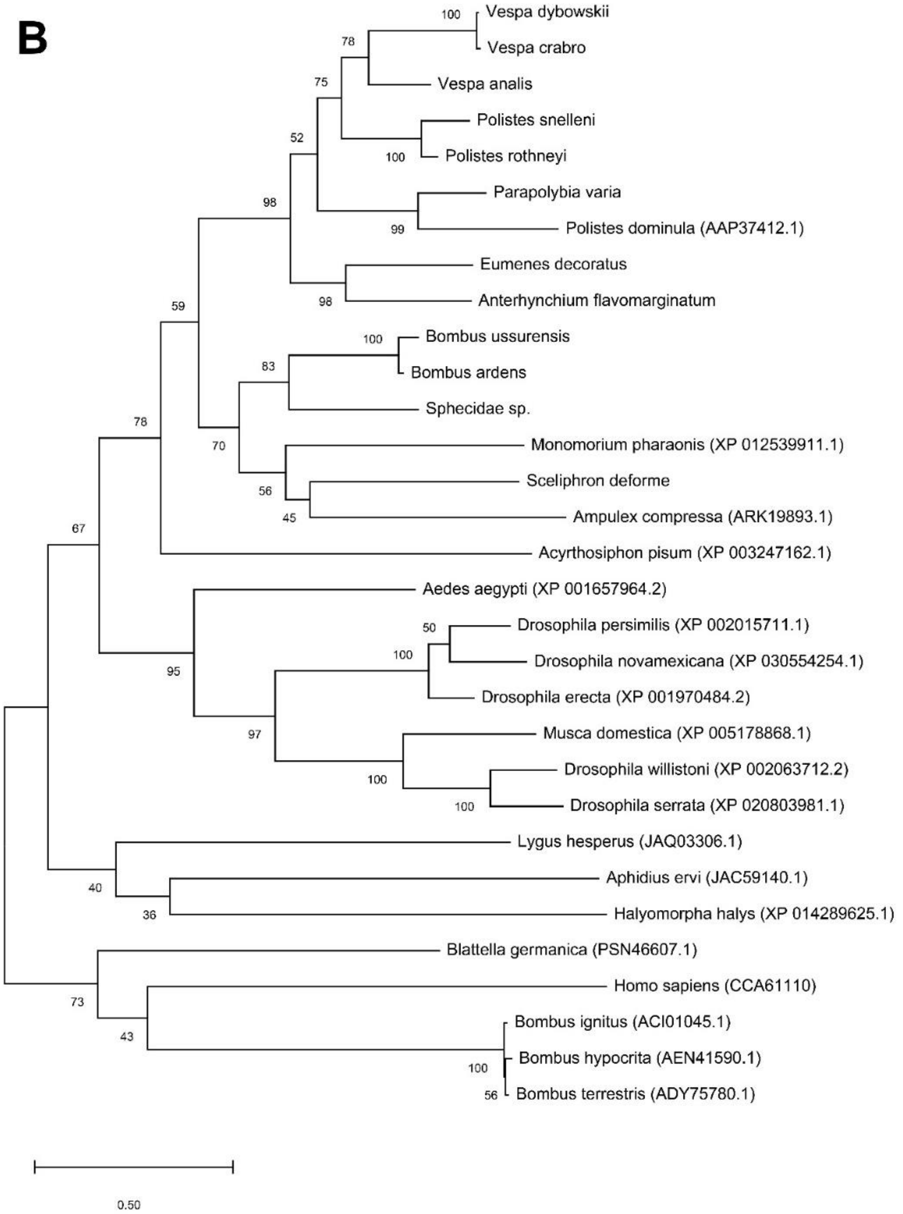
B
Vespa dybowskii
Vespa crabro
Vespa analis
Polistes snelleni
Polistes rothneyi
Parapolybia varia
Polistes dominula (AAP37412.1)
Eumenes decoratus
Anterhynchium flavomarginatum
Bombus ussurensis
Bombus ardens
Sphecidae sp.
Monomorium pharaonis (XP 012539911.1)
Sceliphron deforme
Ampulex compressa (ARK19893.1)
Acyrthosiphon pisum (XP 003247162.1)
Aedes aegypti (XP 001657964.2)
Drosophila persimilis (XP 002015711.1)
Drosophila novamexicana (XP 030554254.1)
Drosophila erecta (XP 001970484.2)
Musca domestica (XP 005178868.1)
Drosophila willistoni (XP 002063712.2)
Drosophila serrata (XP 020803981.1)
Lygus hesperus (JAQ03306.1)
Aphidius ervi (JAC59140.1)
Halyomorpha halys (XP 014289625.1)
Blattella germanica (PSN46607.1)
Homo sapiens (CCA61110)
Bombus ignitus (ACI01045.1)
Bombus hypocrita (AEN41590.1)
Bombus terrestris (ADY75780.1)
0.50

Figure S24. Amino acid alignments of serine protease. A) Alignment of amino acid sequences from V. crabro, V. analis, V. dybowskii, P. varia, P. snelleni, P. rothneyi, A. flavomarginatum, E. decorates, S. deforme, Sphecidae sp., B. ardens, B. ussurensis and H. sapiens. B) Phylogenetic analysis of serine protease.

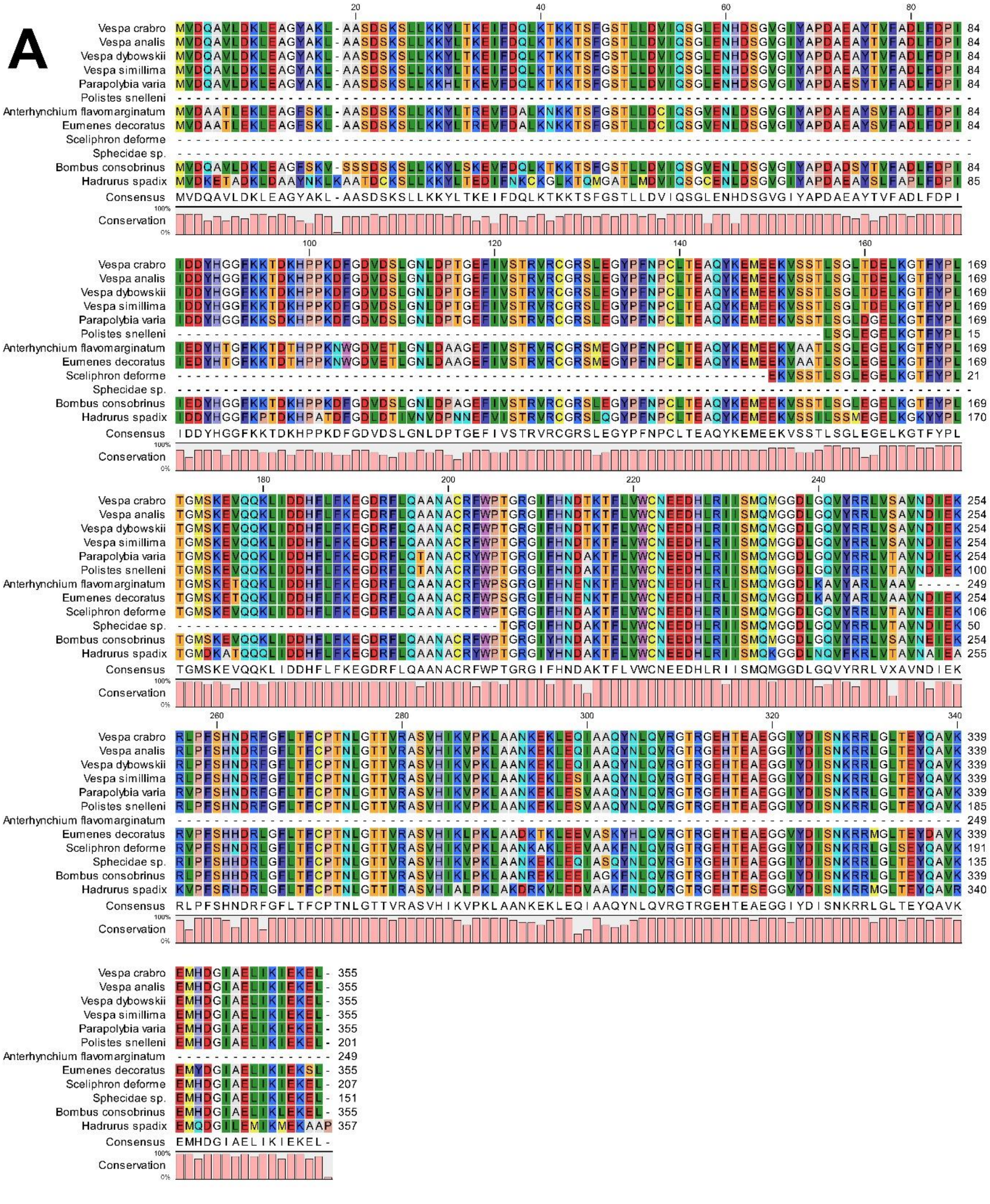

Figure S25B

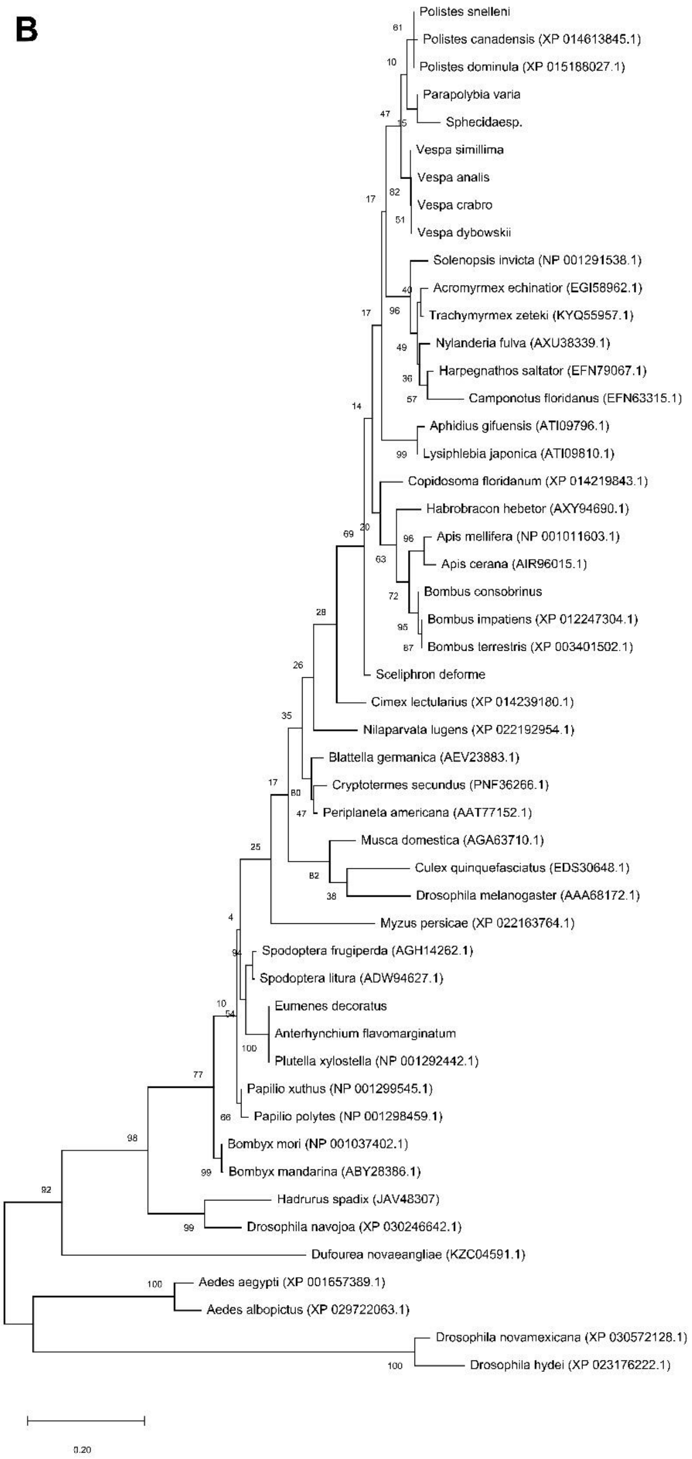

Figure S25. Amino acid alignments of arginine kinase. A) Alignment of amino acid sequences from V. crabro, V. analis, V. dybowskii, V. simillima, P. varia, P. snelleni, A. flavomarginatum, E. decoratus, S. deforme, Sphecidae sp., B. consobrinus and Hadrurus spadix. B) Phylogenetic analysis of arginine kinase.

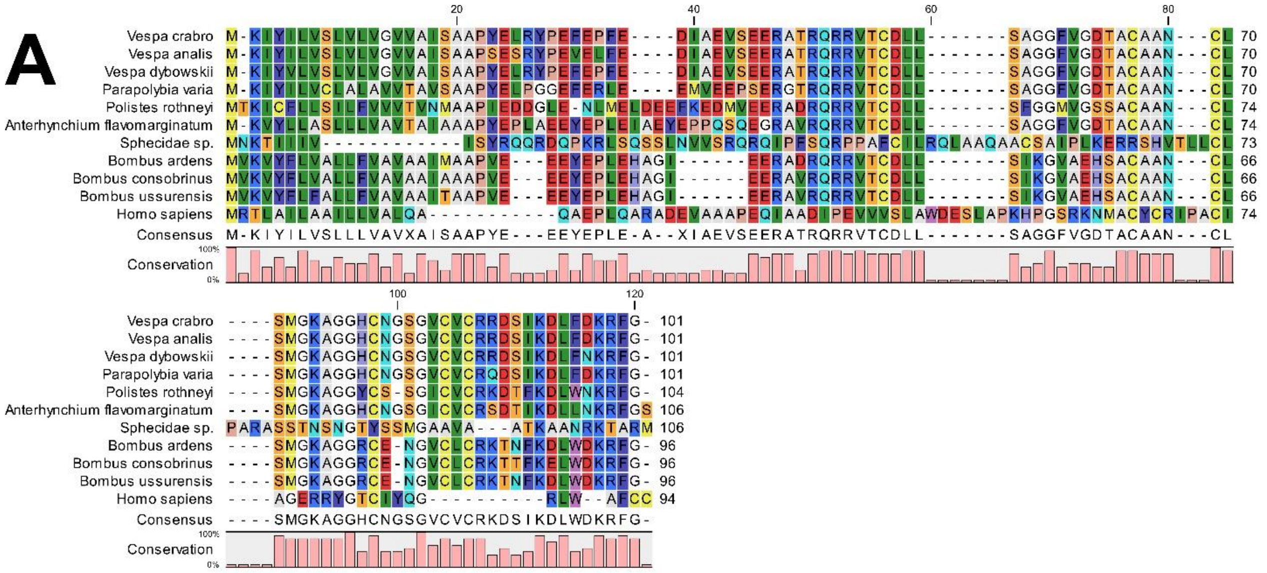

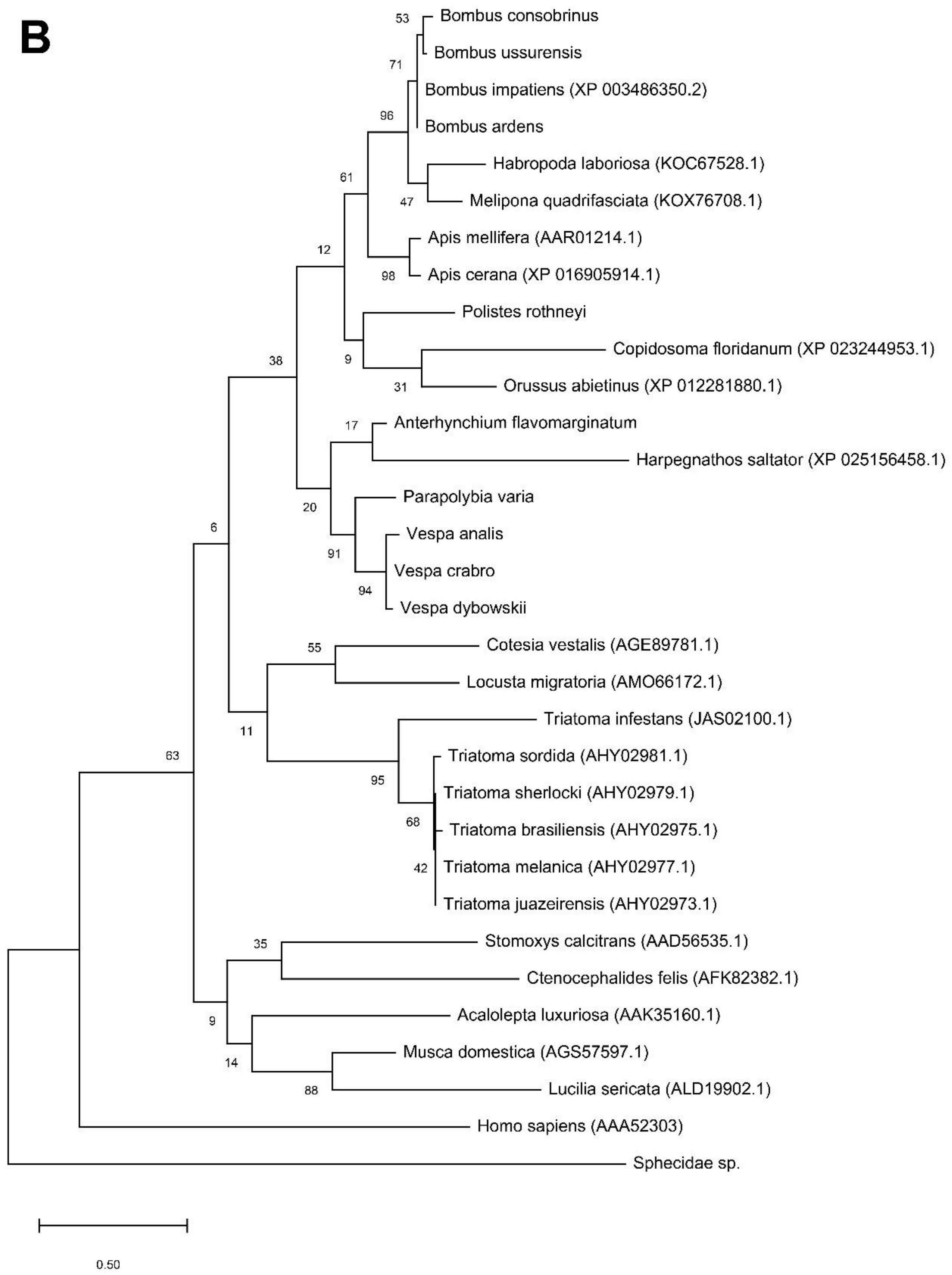

Figure S26. Amino acid alignments of defensin 1. A) Alignment of amino acid sequences from *V. crabro*, *V. analis*, *V. dybowskii*, *P. varia*, *P. rothneyi*, *A. flavomarginatum*, *Sphecidae* sp., *B. ardens*, *B. consobrinus*, *B. ussurensis* and *H. sapiens*. B) Phylogenetic analysis of defensin 1.

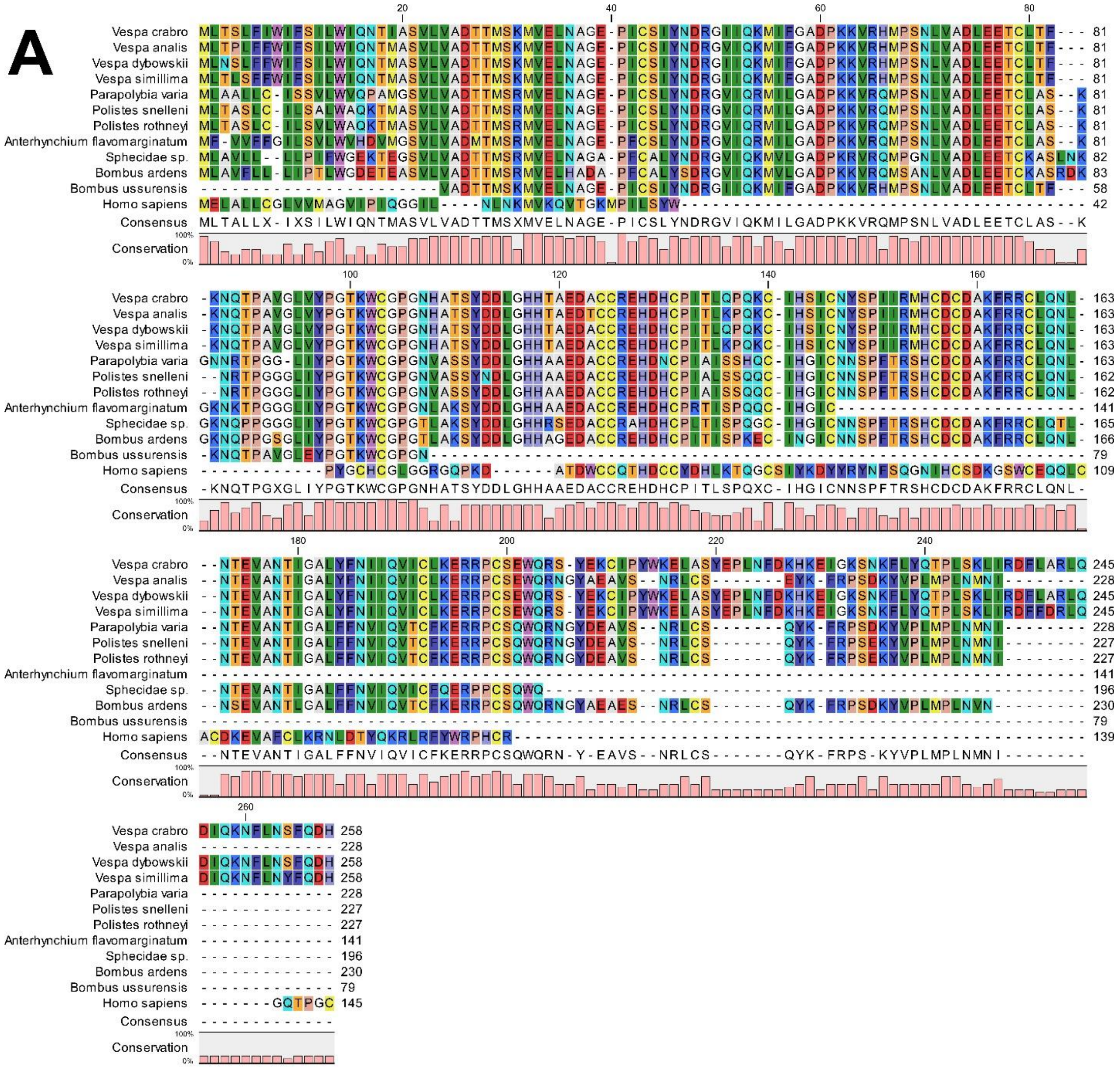

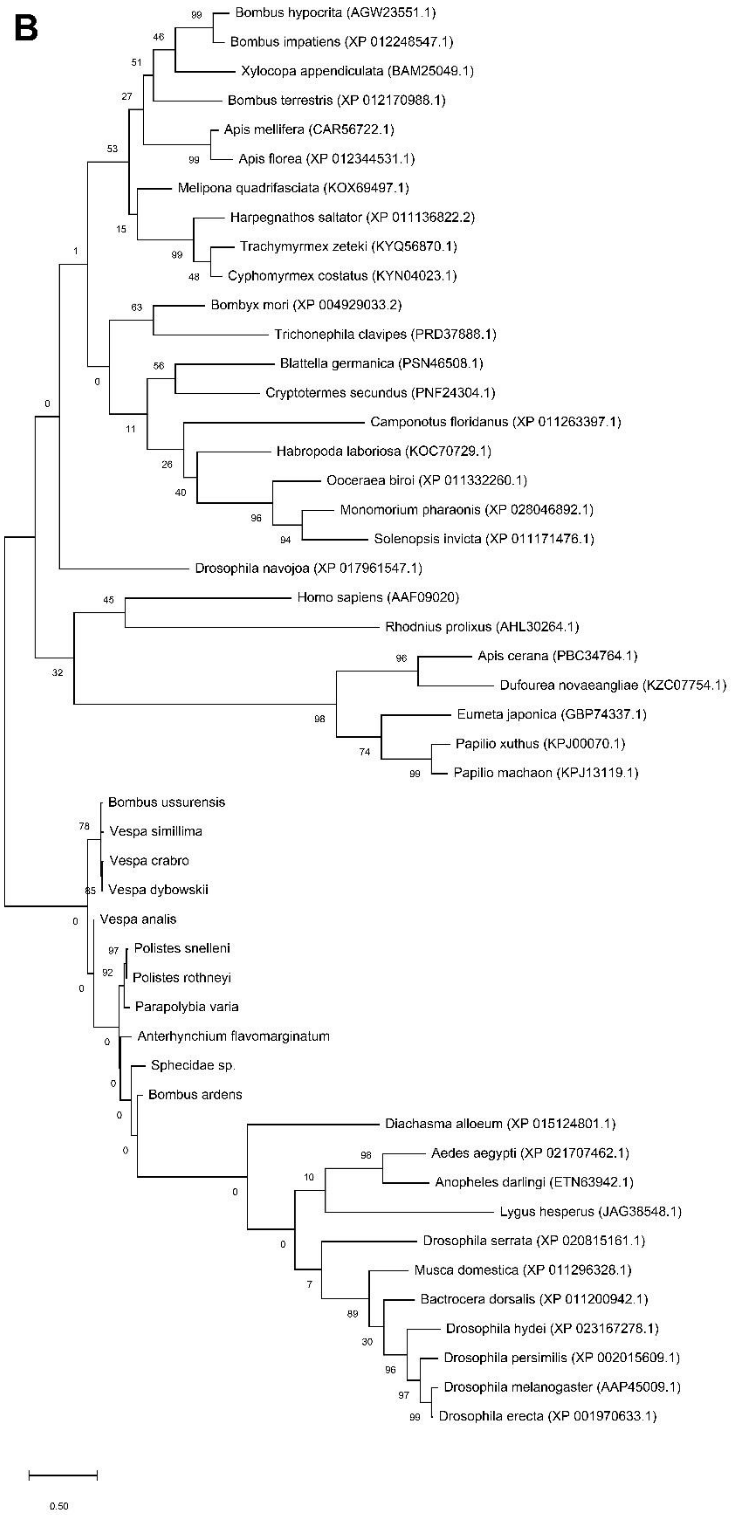

Figure S27. Amino acid alignments of phospholipase A2. A) Alignment of amino acid sequences from *V. crabro*, *V. analis*, *V. dybowskii*, *V. simillima*, *P. varia*, *P. rothneyi*, *A. flavomarginatum*, *Sphecidae* sp., *B. ardens*, *B. ussurensis* and *H. sapiens*. B) Phylogenetic analysis of phospholipase A2.

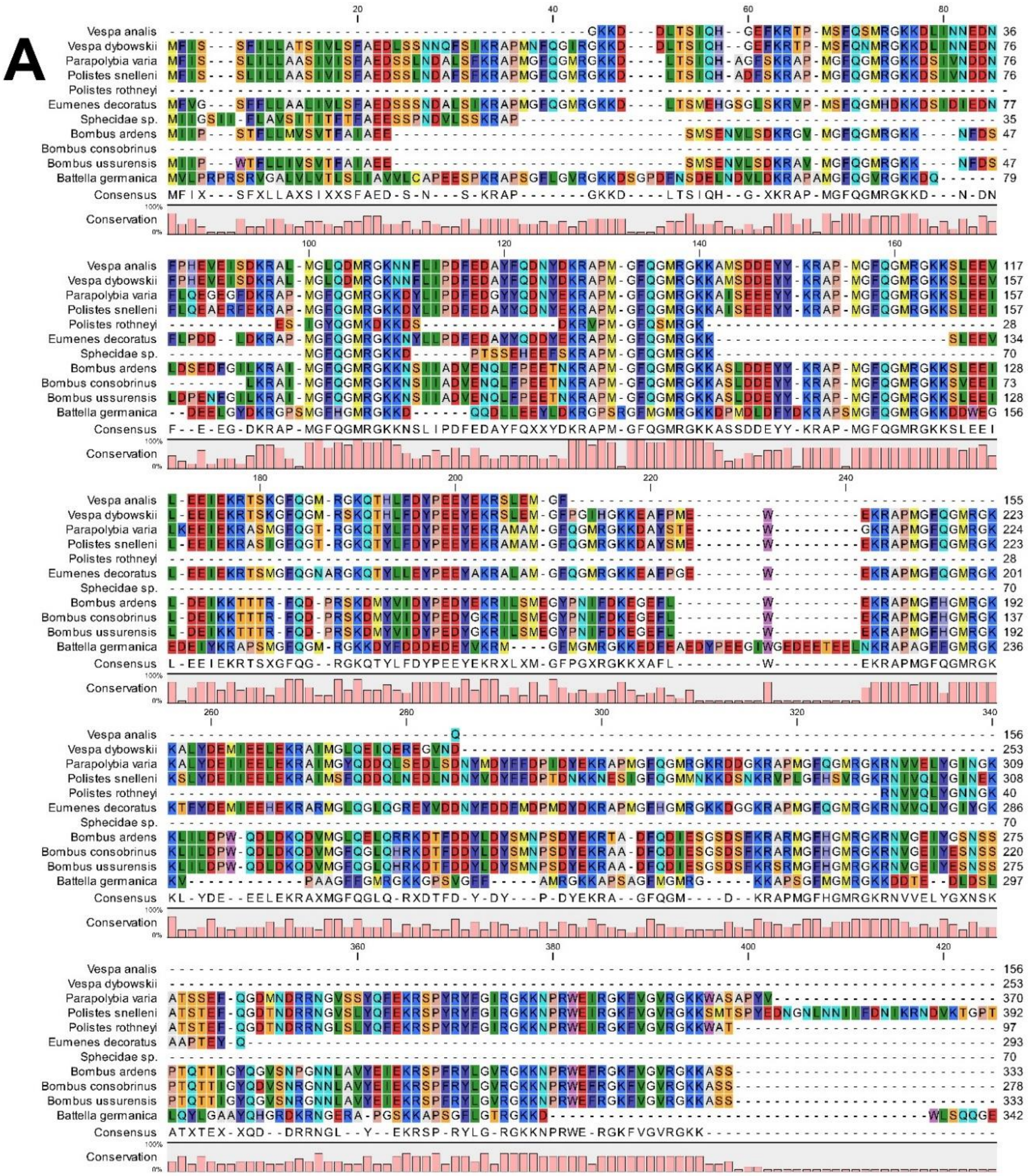
A
Vespa analis
Vespa dybowskii
Parapolybia varia
Polistes snelleni
Polistes rothneyi
Eumenes decoratus
Sphecidae sp.
Bombus ardens
Bombus consobrinus
Bombus ussurensis
Battella germanica
Consensus
Conservation

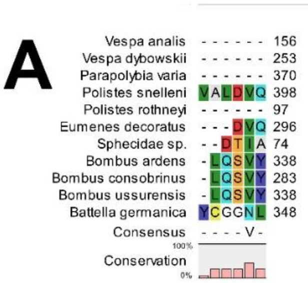
A
Vespa analis
Vespa dybowskii
Parapolybia varia
Polistes snelleni
Polistes rothneyi
Eumenes decoratus
Sphecidae sp.
Bombus ardens
Bombus consobrinus
Bombus ussurensis
Battella germanica
Consensus
Conservation

Figure S28B

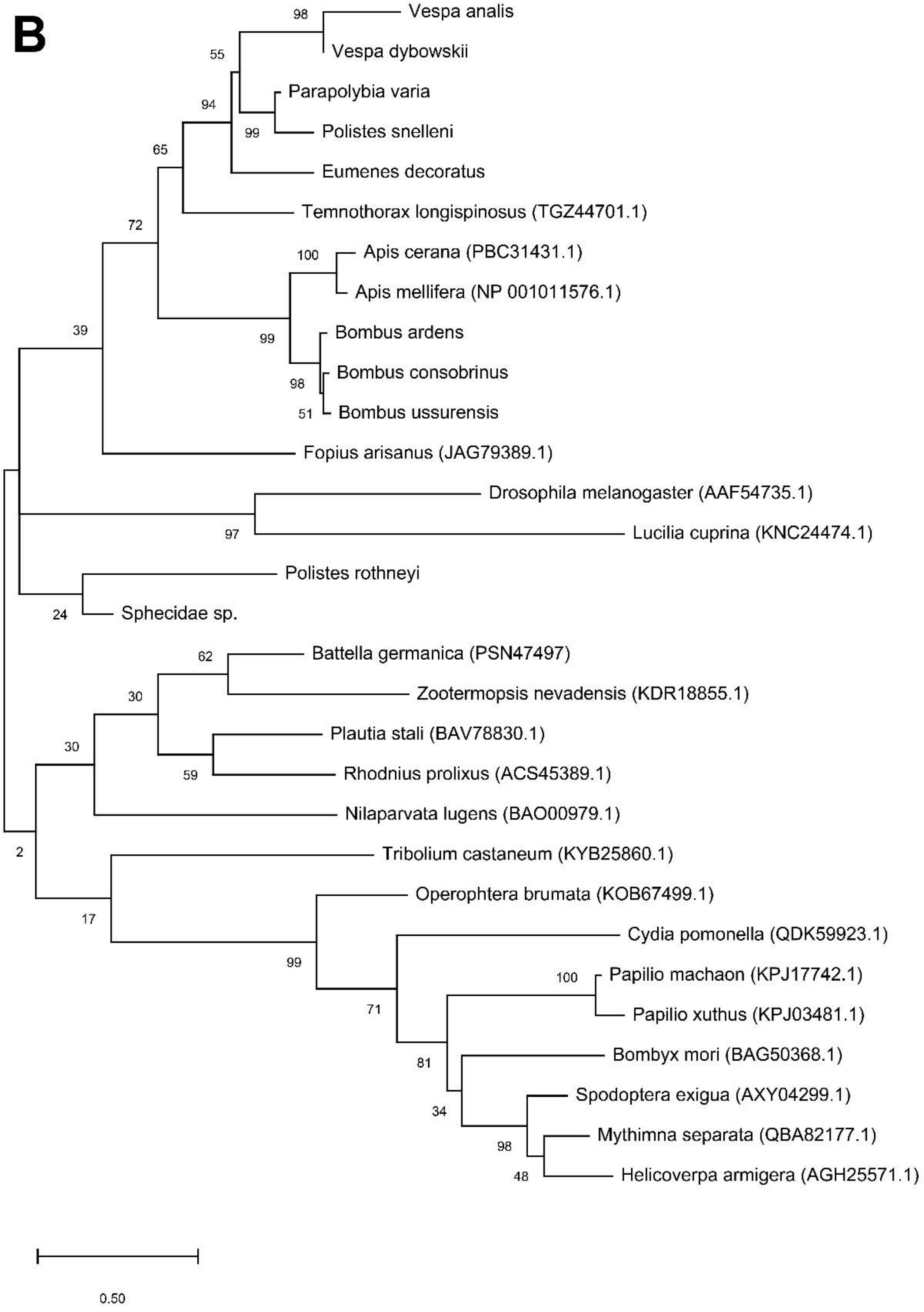

Figure S28. Amino acid alignments of tachykinin. A) Alignment of amino acid sequences from *V. analis*, *V. dybowskii*, *P. varia*, *P. snelleni*, *P. rothneyi*, *E. decorates*, *Sphecidae* sp., *B. ardens*, *B. consobrinus*, *B. ussurensis*, and *B. germanica*. B) Phylogenetic analysis of tachykinin.

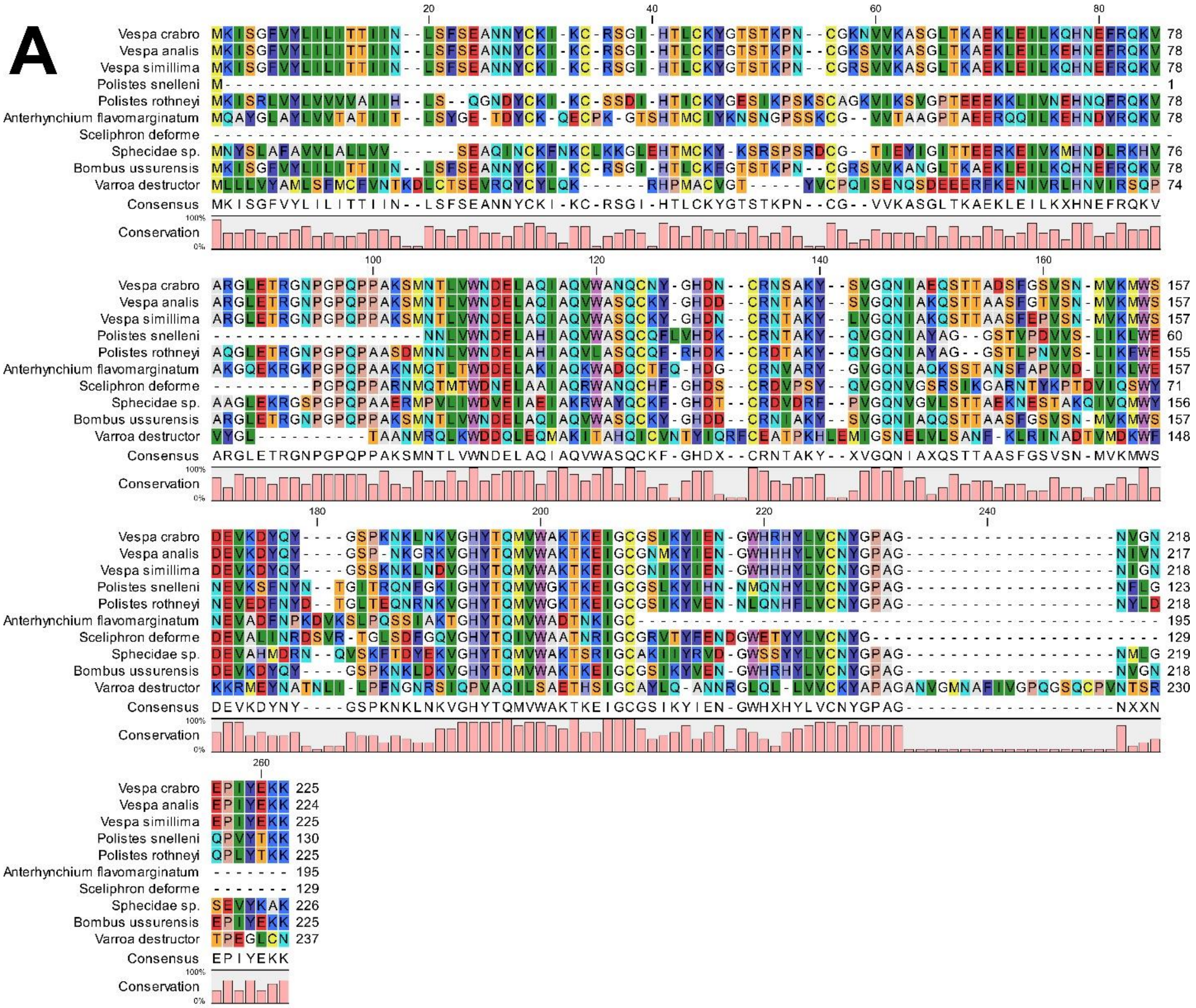
A
Vespa crabro
Vespa analis
Vespa simillima
Polistes snelleni
Polistes rothneyi
Anterhynchium flavomarginatum
Sceliphron deforme
Sphecidae sp.
Bombus ussurensis
Varroa destructor
Consensus
Conservation

Figure S29B

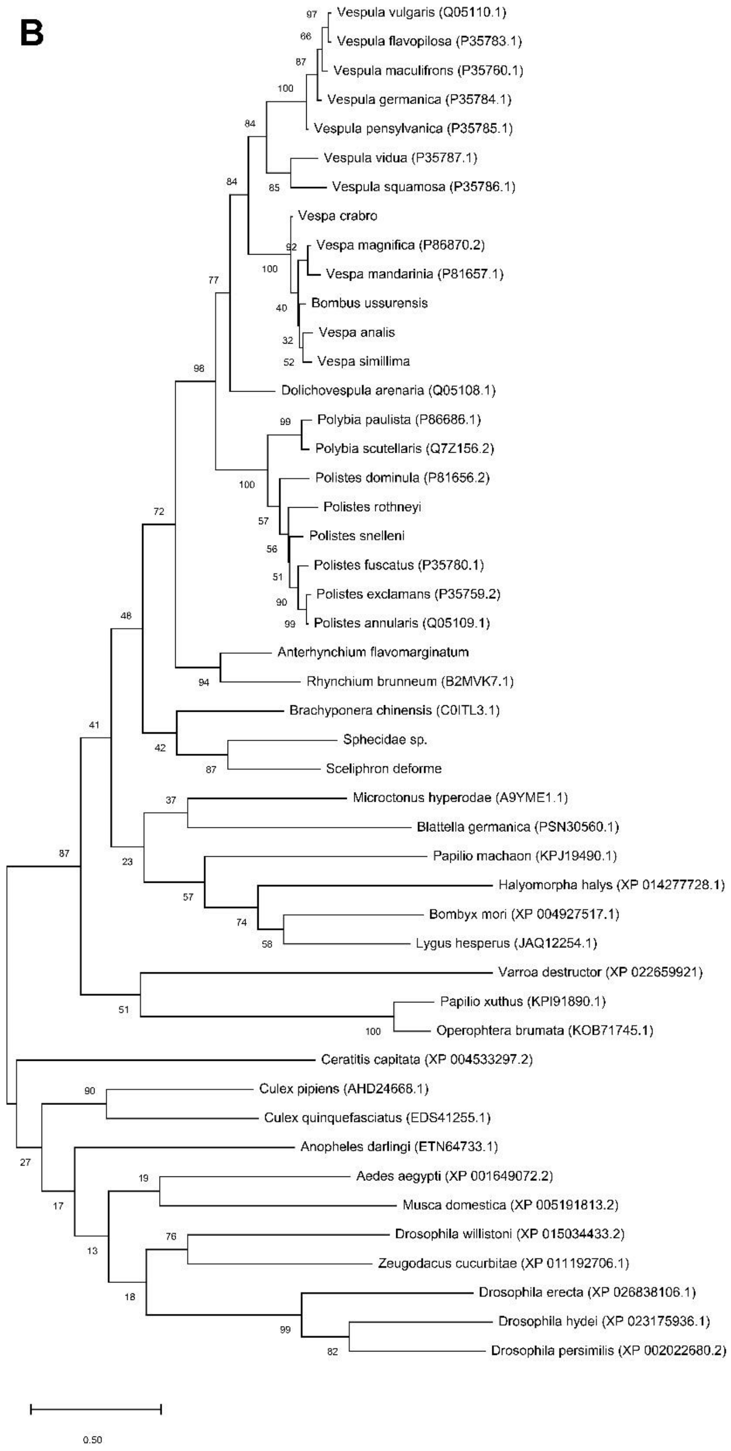

Figure S29. Amino acid alignments of venom allergen 5. A) Alignment of amino acid sequences from *V. crabro, V. analis, V. simillima, P. snelleni, P. rothneyi, A. flavomarginatum, S. deforme, Sphecidae* sp., *B. ussurensis* and *Varroa destructor*. B) Phylogenetic analysis of venom allergen 5.

Table S1. Annotation of top 100 highly expressed genes in the venom gland of *Vespa analis*.

Putative venom protein/peptide	Length (aa)	E-value	TPM
NA	110		87454.8
NA	908		78311
NA	1950		42608.8
apidaecins type 22-like isoform X1	112	3.18167E-14	27389.3
cytochrome P450 4g15	559	0	23351.3
pheromone-binding protein Gp-9-like	152	6.42952E-09	18405.8
PREDICTED: uncharacterized protein LOC107072057	103	1.26354E-51	17769
protein lethal(2)essential for life-like	213	4.24012E-73	9972.79
fatty acid synthase	141	7.73699E-33	6366.62
ejaculatory bulb-specific protein 3-like	129	1.11696E-76	6284.32
endocuticle structural glycoprotein SgAbd-1-like	163	1.2089E-100	6209.02
fatty acid synthase-like	149	1.00418E-59	5957.04
protein lethal(2)essential for life-like	203	3.8496E-106	5808.01
fatty acid synthase	881	0	5141.38
circadian clock-controlled protein-like	248	4.4746E-141	5059.77
fatty acid synthase-like	130	1.48013E-30	4589.06
fatty acid synthase-like	546	0	4232.51
uncharacterized protein LOC107263032	103	3.72714E-08	4173.15
fatty acid synthase-like	1173	0	3780.27
NADH dehydrogenase subunit 1 (mitochondrion)	112	2.03753E-62	3609.25
polyubiquitin isoform X1	609	0	3539.51
superoxide dismutase [Cu-Zn]	155	2.04388E-98	3458.89
PREDICTED: uncharacterized protein LOC106787992	301	0	3060.77
coiled-coil-helix-coiled-coil-helix domain-containing protein 2	154	1.29397E-68	2958.1
heat shock 70 kDa protein cognate 4	648	0	2852.19
PREDICTED: uncharacterized protein LOC106785155	262	1.5009E-126	2684.34
thioredoxin-2	105	1.92448E-63	2664.05
PREDICTED: uncharacterized protein LOC108579915	340	1.72695E-93	2640.18
ferritin subunit	216	4.53228E-95	2625.15
fatty acid synthase	105	9.42896E-26	2621.26
chitinase-like protein Idgf4	434	0	2608.84
40S ribosomal protein S3a	267	0	2466.92
general odorant-binding protein 56d-like	168	8.88272E-75	2430.01
PREDICTED: uncharacterized protein LOC106793109	222	6.4981E-136	2401.66
ubiquitin-60S ribosomal protein L40-like isoform X1	128	5.68352E-91	2384.4
peptidoglycan-recognition protein 2-like	186	2.3595E-99	2353.4
fatty acid synthase	157	1.66649E-69	2237.27
ATP synthase lipid-binding protein, mitochondrial	137	7.9294E-77	2185.08
vitellogenin-like	1759	0	2138.9
ferritin light chain, oocyte isoform	219	3.06322E-84	2015.01
tubulin alpha-1 chain-like	450	0	1937.32
40S ribosomal protein S25	119	1.4342E-80	1861.56
PREDICTED: uncharacterized protein LOC105667370	132	7.83238E-72	1850.5
heat shock protein 70 A2-like	637	0	1832.15
PREDICTED: uncharacterized protein LOC106793189	221	1.99622E-89	1775.94
probable Bax inhibitor 1	236	4.1883E-139	1720.58
peptidoglycan-recognition protein SC2-like	198	4.5899E-100	1712.61
60S ribosomal protein L18	188	7.4137E-129	1704.56
40S ribosomal protein S17	130	3.29733E-92	1700.91
putative fatty acyl-CoA reductase CG5065	540	0	1658.5
cathepsin L	339	0	1653.23
protein lethal(2)essential for life-like	199	4.789E-120	1647.92
multiple epidermal growth factor-like domains protein 10	439	0	1636.78
60S acidic ribosomal protein P2	113	3.91801E-45	1623.52
40S ribosomal protein S26	114	1.2361E-79	1614.36
PREDICTED: uncharacterized protein LOC107067669	154	8.59526E-30	1613.6
sorbitol dehydrogenase-like	339	0	1611.55
cytochrome c oxidase subunit 5B, mitochondrial-like	119	1.0331E-75	1591.03

heat shock protein 83	719	0	1567.01
very-long-chain 3-oxoacyl-CoA reductase-like isoform X2	328	0	1552.89
60S ribosomal protein L44-like	104	9.86798E-70	1547.4
gamma-glutamyl hydrolase-like	322	3.1087E-141	1532.77
icarapin-like	214	1.1551E-102	1494.51
TNF receptor-associated factor family protein DDB_G0272098-like	681	6.91366E-50	1474.55
fatty acid synthase-like	105	7.84848E-36	1460.32
60S acidic ribosomal protein P1	113	4.31168E-50	1422.12
60S ribosomal protein L36	115	1.67197E-76	1406.9
iron-sulfur cluster assembly enzyme ISCU, mitochondrial isoform X2	167	1.7596E-111	1406.46
40S ribosomal protein S8-like	208	2.1546E-150	1378.01
ecdysteroid-regulated 16 kDa protein	155	1.62337E-92	1374.79
elongation of very long chain fatty acids protein AAEL008004 isoform X2	321	0	1357.76
inositol oxygenase	295	0	1356.77
60S ribosomal protein L13	219	1.4312E-141	1346.79
NA	105		1340.61
40S ribosomal protein S24	133	1.8262E-88	1339.09
40S ribosomal protein S16-like	148	5.9546E-103	1301.65
40S ribosomal protein S15Aa	130	3.48336E-92	1269.09
60S ribosomal protein L31	123	1.75486E-87	1256.52
ubiquitin-40S ribosomal protein S27a	156	3.7254E-112	1250.64
40S ribosomal protein S11-like	155	3.0677E-111	1245.13
60S ribosomal protein L27a	147	9.4128E-100	1237.26
60S ribosomal protein L34-like	119	7.26154E-81	1217.25
60S ribosomal protein L23	140	3.81261E-97	1214.9
40S ribosomal protein S3	241	1.6939E-176	1214.37
40S ribosomal protein S23	143	9.2454E-101	1209
putative fatty acyl-CoA reductase CG5065	509	0	1195.7
leucine-rich repeat-containing protein 15-like	632	0	1189.03
elongation of very long chain fatty acids protein AAEL008004-like	260	2.93806E-91	1161.08
cytochrome c-2	108	1.47226E-74	1159.42
NA	117		1148.32
sorbitol dehydrogenase-like isoform X1	349	0	1147.95
60S ribosomal protein L11 isoform X2	197	1.4024E-135	1147.6
peptidyl-prolyl cis-trans isomerase-like	209	1.1551E-141	1137.63
40S ribosomal protein S4-like	262	0	1136.24
40S ribosomal protein S7	193	2.3281E-138	1127.23
40S ribosomal protein S10-like	159	3.3052E-110	1127.02
60S ribosomal protein L10	219	1.0592E-162	1125.34
translationally-controlled tumor protein homolog	172	5.54E-120	1121.22
MD-2-related lipid-recognition protein-like	169	1.9239E-99	1120.33
60S ribosomal protein L5	297	0	1113.35

Table S2. Annotation of top 100 highly expressed genes in the venom gland of *Vespa crabro*.

Putative venom protein/peptide	Length (aa)	E-value	TPM
venom allergen 5 precursor	225	2.2134E-93	150567
phospholipase A1-like	333	2.2333E-122	49149
NA	103		40090.3
phospholipase A1	336	2.0077E-131	17931.7
hyaluronidase	363	1.5998E-158	10228.7
hyaluronidase	357	0	7237.97
phospholipase A1-like	147	1.0798E-29	6969.16
40S ribosomal protein S3a	270	0	5173.18
acidic phospholipase A2 PA4	258	4.2512E-118	4722.07
ubiquitin-60S ribosomal protein L40-like isoform X1	128	5.68352E-91	4353.79
60S ribosomal protein L18	188	2.2918E-129	4104.13
thioredoxin-2	105	6.42154E-64	4067.02
venom dipeptidyl peptidase 4 isoform X1	776	0	3726.38
40S ribosomal protein S25	119	1.4342E-80	3647.54
NADH dehydrogenase subunit 1 (mitochondrion)	111	5.49533E-62	3629.56
polyubiquitin isoform X1	447	0	3579.21
NA	105		3478.14
40S ribosomal protein S16-like	148	5.9546E-103	3467.11
60S ribosomal protein L23	140	3.81261E-97	3454.68
PREDICTED: uncharacterized protein LOC105667370	132	7.83238E-72	3409.23
40S ribosomal protein S17	130	3.29733E-92	3361.41
NA	105		3344.44
translation elongation factor 2	844	0	3341.65
40S ribosomal protein S8-like	208	2.1546E-150	3196.12
NA	104		3156.64
40S ribosomal protein S26	114	1.2361E-79	3138.78
40S ribosomal protein S5	215	7.3059E-157	3104.45
40S ribosomal protein S15Aa	130	3.48336E-92	3065.46
60S acidic ribosomal protein P2	113	1.12343E-45	2913.04
ubiquitin-40S ribosomal protein S27a	156	3.7254E-112	2824.1
40S ribosomal protein S20	121	2.89712E-82	2779.94
40S ribosomal protein S11-like	155	3.0677E-111	2775.21
60S ribosomal protein L9	210	3.9774E-142	2768.23
heat shock 70 kDa protein cognate 4	648	0	2767.16
60S acidic ribosomal protein P0	317	0	2760.96
60S ribosomal protein L28	138	1.31109E-90	2738.18
60S ribosomal protein L10	219	1.0592E-162	2678.74
uncharacterized protein LOC105432314	111	2.25403E-22	2677.41
40S ribosomal protein S4-like	262	0	2661.91
NA	101		2641.18
60S ribosomal protein L5	297	0	2587.42
60S ribosomal protein L26	148	1.2951E-103	2550.47
ATP synthase lipid-binding protein, mitochondrial	137	7.9294E-77	2491.2
translationally-controlled tumor protein homolog	172	5.54E-120	2480.36
40S ribosomal protein S23	143	9.2454E-101	2457.48
polyubiquitin-A isoform X3	291	0	2445.81
60S ribosomal protein L44-like	104	9.86798E-70	2439.31
eukaryotic initiation factor 4A-I	423	0	2412.44
death-associated protein 1	102	1.2317E-67	2366.63
40S ribosomal protein S13	151	4.2192E-108	2288.46
60S ribosomal protein L13a	213	4.5379E-147	2222.75
60S ribosomal protein L22-like	139	3.87926E-95	2201.51
40S ribosomal protein S10-like	159	3.3052E-110	2195.2
60S ribosomal protein L11 isoform X2	195	2.2129E-135	2157.09
60S ribosomal protein L34-like	119	7.26154E-81	2155.72
coiled-coil-helix-coiled-coil-helix domain-containing protein 2, mitochondrial	154	3.00812E-68	2126.6
60S ribosomal protein L31	164	8.14544E-87	2098.98
40S ribosomal protein S3	241	1.6939E-176	2094.33

elongation factor 1-alpha-like	462	0	2050.05
40S ribosomal protein S2	299	0	1980.91
60S acidic ribosomal protein P1	113	7.91728E-71	1964.2
60S ribosomal protein L23a	247	1.0812E-134	1959.15
60S ribosomal protein L17	185	4.8212E-137	1942.5
60S ribosomal protein L4	430	0	1920.49
40S ribosomal protein S9 isoform X1	193	3.1021E-137	1915.35
60S ribosomal protein L13	219	1.4312E-141	1876.92
pheromone-binding protein Gp-9-like	155	1.29655E-11	1798.82
40S ribosomal protein S6-like	253	0	1764.43
60S ribosomal protein L7a	268	0	1756.17
superoxide dismutase [Cu-Zn]	155	2.04388E-98	1746.1
60S ribosomal protein L27a	147	9.4128E-100	1742.13
40S ribosomal protein S7	193	2.3281E-138	1734.61
60S ribosomal protein L24	170	3.4322E-108	1732.99
60S ribosomal protein L21	159	3.539E-114	1722.9
60S ribosomal protein L35a	146	4.3344E-99	1675
60S ribosomal protein L14	152	5.20947E-94	1673.07
elongation factor 1-alpha	462	0	1657.72
60S ribosomal protein L7	251	9.5337E-175	1657.47
neuromodulin-like	123	3.60628E-75	1648.97
40S ribosomal protein S24	182	4.27727E-88	1632.48
60S ribosomal protein L32	134	1.06E-95	1632.36
NA	112		1592.55
small integral membrane protein 14	119	1.2993E-41	1580.27
40S ribosomal protein SA	310	0	1568.71
60S ribosomal protein L36	116	2.00284E-73	1564.55
polyadenylate-binding protein 1	630	0	1564.04
eukaryotic translation initiation factor 5A	160	6.0794E-117	1531.03
60S ribosomal protein L35	123	2.35088E-75	1530.74
60S ribosomal protein L6-like	294	2.0469E-164	1489.7
peptidyl-prolyl cis-trans isomerase FKBP1A	109	6.27042E-73	1488.98
60S ribosomal protein L18a isoform X1	177	2.1819E-129	1482.82
40S ribosomal protein S14	151	4.0919E-107	1466.24
40S ribosomal protein S12	141	3.15101E-98	1453.13
heat shock 70 kDa protein cognate 3 isoform X1	660	0	1420.89
60S ribosomal protein L19	213	1.3511E-142	1411.92
ADP,ATP carrier protein	300	0	1402.05
40S ribosomal protein S19	172	8.4478E-110	1374.99
60S ribosomal protein L30	114	2.38148E-80	1373.81
protein lethal(2)essential for life-like	281	6.51907E-78	1355.37
probable Bax inhibitor 1	236	8.4515E-140	1324.37

Table S3. Annotation of top 100 highly expressed genes in the venom gland of *Vespa dybowskii*.

Putative venom protein/peptide	Length (aa)	E-value	TPM
phospholipase A1-like	269	7.93E-86	106395
phospholipase A1-like	165	1.16E-72	79401.2
venom allergen 5 precursor	225	4.04E-93	65462.1
NA	103		36744.6
phospholipase A1-like	269	2.72E-82	9560.53
small integral membrane protein 14	107	1.88E-32	6491.82
death-associated protein 1	102	1.23E-67	5498.15
hyaluronidase	335	1.31E-180	4566.41
translation elongation factor 2	137	2.23E-78	4476.1
pheromone-binding protein Gp-9-like	153	7.65E-11	4156.36
cytochrome P450 4g15	559	0	4033.09
hyaluronidase	359	7.15E-153	3902.61
60S ribosomal protein L31	104	4.09E-48	3875.06
60S ribosomal protein L5-like	113	1.34E-74	3213.1
protein lethal(2)essential for life-like	275	2.17E-67	3163.41
40S ribosomal protein S26	114	1.24E-79	3126.55
60S acidic ribosomal protein P2	113	1.70E-39	2890.28
phospholipase A1 2-like	170	2.81E-52	2827.42
polyubiquitin-B isoform X1	260	0	2557.65
PREDICTED: uncharacterized protein LOC105667370	132	6.95E-63	2543.59
translation elongation factor 2	844	0	2393.31
NA	106		2262.34
60S ribosomal protein L10	111	9.43E-79	2254.17
ATP synthase lipid-binding protein, mitochondrial	137	7.09E-34	2198.42
venom dipeptidyl peptidase 4 isoform X1	776	0	2175.87
NA	103		2160.17
ADP,ATP carrier protein 2	300	0	2148.53
PREDICTED: uncharacterized protein LOC106784194	173	6.37E-55	2125.47
coiled-coil-helix-coiled-coil-helix domain-containing protein 10, mitochondrial	154	2.10E-45	2114.62
40S ribosomal protein S25	119	4.39E-50	2078.58
apidaecins type 22-like isoform X2	172	6.06E-14	1996.14
NA	102		1985.15
phospholipase A1-like	169	9.75E-74	1944.05
60S acidic ribosomal protein P1 isoform X1	113	2.35E-37	1924.34
BCL2/adenovirus E1B 19 kDa protein-interacting protein 3 isoform X2	107	3.61E-46	1772.15
probable Bax inhibitor 1	236	3.23E-131	1771.97
acyl-CoA Delta(11) desaturase-like	367	0	1723.81
superoxide dismutase [Cu-Zn]	155	2.04E-98	1701.54
NA	106		1675.7
phospholipase A1-like	99	3.96E-15	1666.26
PREDICTED: uncharacterized protein LOC100881835	130	7.14E-41	1583.69
NA	102		1567.1
40S ribosomal protein S18 isoform X1	101	6.94E-67	1562.59
eukaryotic initiation factor 4A-I	218	2.49E-159	1558.58
NA	102		1547.47
acidic phospholipase A2 PA4	258	2.18E-118	1546.9
heat shock 70 kDa protein cognate 4	648	0	1540.24
40S ribosomal protein S24	130	7.01E-78	1524.66
60S ribosomal protein L17	185	4.64E-128	1412.79
PREDICTED: uncharacterized protein LOC107072577 isoform X2	296	1.39E-180	1408.73
PREDICTED: uncharacterized protein LOC107074702	109	4.28E-12	1406.67
NA	144		1385.15
acidic leucine-rich nuclear phosphoprotein 32 family member B-like	115	6.91E-09	1373.07
acidic leucine-rich nuclear phosphoprotein 32 family member B-like	115	6.91E-09	1373.07
NA	103		1369.59
eukaryotic translation initiation factor 4E-binding protein 3	114	2.03E-54	1365.43
translationally-controlled tumor protein homolog	172	4.89E-108	1347.2
translationally-controlled tumor protein homolog	172	4.89E-108	1347.2

acidic phospholipase A2 PA4-like isoform X2	228	4.29E-143	1327.03
peroxiredoxin 1	193	4.00E-133	1304.85
60S acidic ribosomal protein P0	317	0	1304.18
venom serine protease-like	390	1.55E-175	1300.86
NA	111		1299.19
polyubiquitin-B	175	2.73E-116	1266.39
glyceraldehyde-3-phosphate dehydrogenase 1-like	194	1.10E-127	1266.33
NA	110		1260.44
NA	110		1260.44
40S ribosomal protein S19	154	7.07E-100	1252.99
40S ribosomal protein S17	168	8.45E-92	1234.17
40S ribosomal protein S8	208	8.50E-142	1230.63
protein FAM195A-like isoform X2	106	9.86E-68	1229.86
40S ribosomal protein S16-like	148	1.05E-103	1216.15
chitinase-like protein Idgf4	253	2.47E-169	1206.69
PREDICTED: uncharacterized protein LOC106793063	195	8.59E-94	1198.74
40S ribosomal protein S2	281	4.08E-158	1190.81
60S ribosomal protein L13a	204	2.76E-147	1189.41
60S ribosomal protein L30	114	2.38E-80	1171.09
bifunctional glutamate/proline--tRNA ligase	103	1.22E-35	1170.22
icarapin-like	220	8.96E-99	1166.85
NA	100		1160.17
CCHC-type zinc finger protein CG3800	154	2.08E-87	1146.23
40S ribosomal protein S13	151	4.22E-108	1141.12
activating transcription factor of chaperone	371	0	1135.58
NA	100		1109.51
cytochrome c-2	108	1.47E-74	1090.29
polyadenylate-binding protein 1	630	0	1047.46
NA	105		1037.49
serine-arginine protein 55 isoform X15	112	9.96E-54	1035.61
ATPase inhibitor mai-2, mitochondrial-like	107	4.51E-28	1007.3
NA	103		999
NA	99		986.527
profilin	126	1.25E-89	980.569
40S ribosomal protein S5	215	7.31E-157	977.662
elongation factor 1-alpha	462	0	963.045
60S ribosomal protein L5	297	0	937.6
V-type proton ATPase 16 kDa proteolipid subunit	159	4.88E-99	929.883
proton-coupled amino acid transporter 1	470	0	921.511
BCL2/adenovirus E1B 19 kDa protein-interacting protein 3 isoform X2	193	2.64E-110	916.725
NA	99		910.543
60S ribosomal protein L4	430	0	888.187

Table S3

Table S4. Annotation of top 100 highly expressed genes in the venom gland of *Vespa simillima*.

Putative venom protein/peptide	Length (aa)	E-value	TPM
NA	108		297476
NA	112		39206.8
NA	112		39206.8
venom allergen 5 precursor	225	2.76884E-92	32241.1
uncharacterized protein LOC105432314	111	6.36057E-23	29699.6
phospholipase A1-like	333	5.769E-124	29422.8
NA	112		23327.1
NA	106		14561.6
protein lethal(2)essential for life-like	210	3.83287E-73	5349.74
tryptophan 5-hydroxylase 1	528	0	5193.15
phospholipase A1-like	336	3.97E-125	4314.92
phospholipase A1-like	335	1.6524E-135	4042.11
polyubiquitin-B	194	2.0006E-124	3749.93
coiled-coil and C2 domain-containing protein 1-like isoform X2	154	2.29815E-19	3534.86
tryptophan 5-hydroxylase 1	164	3.7697E-87	3463.45
nuclear protein 1	99	4.71075E-47	2945.97
nuclear protein 1	99	4.71075E-47	2945.97
polyubiquitin	147	1.995E-102	2594.43
ATP synthase lipid-binding protein, mitochondrial	137	5.34903E-35	2280.9
PREDICTED: uncharacterized protein LOC108691125	125	5.10977E-59	2245.91
PREDICTED: uncharacterized protein LOC108691125	125	5.10977E-59	2245.91
PREDICTED: uncharacterized protein LOC106787088	144	6.96387E-51	2160.93
eukaryotic translation initiation factor 4E-binding protein 3	114	2.03375E-54	1829.19
heat shock 70 kDa protein cognate 4	648	0	1820.18
heat shock 70 kDa protein cognate 4	648	0	1820.18
WD repeat domain phosphoinositide-interacting protein 4 isoform X2	344	0	1617.63
fatty acyl-CoA reductase 1	517	0	1616.34
translationally-controlled tumor protein homolog	172	4.8892E-108	1582.16
40S ribosomal protein S26	114	1.2361E-79	1503.24
eukaryotic initiation factor 4A-I	423	0	1486.79
peptidyl-prolyl cis-trans isomerase FKBP1A	109	6.27042E-73	1477.8
hyaluronidase	358	1.0264E-163	1409.24
60S ribosomal protein L34-like	90	2.95828E-58	1398.12
40S ribosomal protein S25	119	4.39327E-50	1385.76
farnesol dehydrogenase-like isoform X2	251	6.9847E-123	1374.34
PREDICTED: uncharacterized protein LOC106791229 isoform X1	564	0	1374.34
death-associated protein 1	102	1.2317E-67	1373.78
ADP,ATP carrier protein	300	0	1341.74
uncharacterized protein LOC113563422	230	2.39125E-18	1291.49
60S ribosomal protein L31	123	1.13224E-78	1218.27
PREDICTED: uncharacterized protein LOC106790403 isoform X1	439	0	1212.27
60S ribosomal protein L26	105	2.88869E-56	1172.2
endocuticle structural glycoprotein SgAbd-1-like	143	3.2667E-100	1131.83
probable Bax inhibitor 1	236	3.2338E-131	1105.94
cytochrome c oxidase subunit 5B, mitochondrial-like	119	8.11477E-76	1105.13
NA	105		1066.8
elongation factor 1-alpha	462	0	1066.8
60S ribosomal protein L10	223	7.3648E-163	1056.64
28S ribosomal protein S14, mitochondrial	159	2.05558E-70	1029.53
60S acidic ribosomal protein P0	317	0	1010.46
60S ribosomal protein L5	297	0	980.012
40S ribosomal protein S20	121	2.89712E-82	960.289
acidic phospholipase A2 PA4	258	3.6582E-119	951.932
acidic phospholipase A2 PA4	258	3.6582E-119	951.932
40S ribosomal protein S17	164	9.06632E-92	949.156
cytochrome c oxidase subunit 6A1, mitochondrial-like	106	1.73943E-21	945.854
gamma-aminobutyric acid receptor-associated protein	117	3.16001E-82	899.93
NADH dehydrogenase subunit 5 (mitochondrion)	212	9.45627E-96	873.711

splicing factor 3A subunit 2	273	2.2795E-150	854.677
small integral membrane protein 14	107	1.8802E-32	840.191
zinc finger CCHC domain-containing protein 8 homolog	613	0	840.191
ecdysteroid-regulated 16 kDa protein	155	8.72462E-85	828.321
superoxide dismutase [Cu-Zn]	155	9.5867E-99	827.882
quinone oxidoreductase isoform X2	402	0	826.635
PREDICTED: uncharacterized protein LOC106783854	124	5.29466E-64	820.009
neuromodulin-like	123	2.23086E-39	811.801
peptidyl-prolyl cis-trans isomerase-like	209	1.1551E-141	800.578
60S ribosomal protein L36	115	1.67197E-76	790.984
NADPH--cytochrome P450 reductase isoform X1	679	0	789.954
choline/ethanolamine kinase isoform X2	113	3.90141E-49	778.563
NA	109		764.437
translation elongation factor 2	844	0	731.363
translation elongation factor 2	844	0	731.363
PREDICTED: uncharacterized protein LOC106790187	115	3.30736E-50	726.037
icarapin-like	220	8.59648E-90	724.779
eukaryotic translation initiation factor 5A	160	6.0794E-117	719.532
aldose reductase-like	317	0	718.15
40S ribosomal protein S14	151	6.07936E-79	713.523
40S ribosomal protein S5	237	3.7218E-156	712.872
BCL2/adenovirus E1B 19 kDa protein-interacting protein 3 isoform X2	193	1.3647E-109	702.966
V-type proton ATPase 16 kDa proteolipid subunit	159	4.8795E-99	698.898
PREDICTED: uncharacterized protein LOC106789874	108	1.22085E-55	694.133
60S ribosomal protein L11	195	1.572E-126	690.449
ATPase inhibitor mai-2, mitochondrial-like	107	3.52276E-27	688.704
coiled-coil-helix-coiled-coil-helix domain-containing protein 10	154	2.09651E-45	682.595
coiled-coil-helix-coiled-coil-helix domain-containing protein 10	154	2.09651E-45	682.595
coiled-coil-helix-coiled-coil-helix domain-containing protein 10	154	2.09651E-45	682.595
40S ribosomal protein S2	281	4.0831E-158	675.056
40S ribosomal protein S16-like	148	1.0513E-103	673.385
60S ribosomal protein L18	188	5.7713E-118	653.329
PREDICTED: uncharacterized protein LOC107064284 isoform X5	120	3.32612E-42	653.329
activating transcription factor of chaperone	371	0	652.104
60S ribosomal protein L13a	216	9.0683E-147	650.813
NA	102		649
PREDICTED: uncharacterized protein LOC106787992	301	0	639.07
PREDICTED: uncharacterized protein LOC106787992	301	0	639.07
PREDICTED: uncharacterized protein LOC106787992	301	0	639.07
40S ribosomal protein S13	152	3.4913E-108	617.856
40S ribosomal protein S7	193	1.1818E-118	616.557
polyubiquitin-like	362	0	608.42

Table S5. Annotation of top 100 highly expressed genes in the venom gland of *Parapolybia varia*.

Putative venom protein/peptide	Length (aa)	E-value	TPM
NA	101		71487.6
PREDICTED: uncharacterized protein LOC107072057	99	1.27082E-49	27704.7
NA	103		16002.4
myosin light chain alkali isoform X2	152	2.1101E-103	14710.7
venom allergen 5 precursor	227	1.9919E-119	13212.1
myosin heavy chain, muscle-like	133	1.9917E-91	9740.15
myosin regulatory light chain 2	148	2.9942E-105	9409.86
vitellogenin-like	1748	0	8934.66
myosin regulatory light chain 2	213	2.5138E-108	6733.3
flexible cuticle protein 12-like	105	1.98352E-61	6608.22
cytochrome P450 4g15	557	0	6579.14
phospholipase A1 1	335	6.7806E-154	6440.22
phospholipase A1 1	335	6.7806E-154	6440.22
NA	130		6382.83
myosin heavy chain, muscle isoform X9	1427	0	6306.11
actin, muscle	376	0	6000.51
actin, muscle	376	0	6000.51
myosin heavy chain, muscle isoform X10	490	0	5856.66
ejaculatory bulb-specific protein 3	104	4.86151E-57	5684.88
NA	165		5475.5
paramyosin, long form	876	0	4916.34
troponin I isoform X11	125	8.5634E-44	4803.56
PREDICTED: uncharacterized protein LOC108778990	112	3.18281E-20	4755.63
arginine kinase	375	0	4687.24
tropomyosin isoform X22	283	6.29E-146	4446.21
endocuticle structural glycoprotein SgAbd-1-like	143	4.3555E-99	4404.39
cytochrome c-2	108	1.09788E-73	4203.66
ATP synthase lipid-binding protein, mitochondrial	138	1.52976E-34	4027.82
troponin T, skeletal muscle isoform X1	397	5.0798E-179	3829.37
acidic leucine-rich nuclear phosphoprotein 32 family member B-like	115	9.03214E-09	3246.53
troponin I isoform X5	212	3.41711E-89	3219.33
actin, muscle	376	0	3175.84
actin, muscle	376	0	3175.84
actin, muscle	376	0	3175.84
ADP,ATP carrier protein 2	300	0	3062.33
PREDICTED: uncharacterized protein LOC106785313 isoform X2	253	3.8595E-168	2961.32
PREDICTED: uncharacterized protein LOC106793063	195	2.741E-119	2610.52
protein lethal(2)essential for life-like isoform X2	100	2.1468E-61	2493.56
NA	107		2482.63
PREDICTED: uncharacterized protein LOC106787274	144	4.14012E-58	2439.31
NADH dehydrogenase subunit 5 (mitochondrion)	237	7.3597E-129	2211.34
ejaculatory bulb-specific protein 3-like	129	1.90255E-79	2167.36
NA	111		2080.7
PREDICTED: uncharacterized protein LOC107997824	116	5.75911E-22	2023.64
GATA zinc finger domain-containing protein 14-like	126	1.76615E-36	1977.89
NA	105		1947.81
tropomyosin-1	284	0	1924.16
tropomyosin-1	284	0	1924.16
NA	100		1913.88
NA	100		1913.88
death-associated protein 1	102	8.3872E-68	1838.31
endocuticle structural glycoprotein SgAbd-4-like	134	5.59819E-71	1826.12
PREDICTED: uncharacterized protein LOC106783771	295	8.4168E-162	1822.18
ATP synthase subunit g, mitochondrial	100	5.94266E-57	1760.04
coiled-coil-helix-coiled-coil-helix domain-containing protein 10, mitochondrial	151	1.27944E-45	1749.93
venom serine protease precursor	275	2.1005E-124	1737.2
four and a half LIM domains protein 2 isoform X8	344	0	1666.69
defensin-1-like	101	1.252E-42	1652.27

NA	108		1648.46
NA	99		1626.26
V-type proton ATPase 16 kDa proteolipid subunit	159	4.3959E-103	1570.66
NA	121		1497.74
NA	103		1456.95
NA	103		1456.95
troponin C, isoform 2-like	159	4.13169E-89	1404.8
putative ATP synthase subunit f, mitochondrial	107	1.49423E-66	1397.64
ATP-dependent RNA helicase p62-like isoform X2	117	3.71513E-55	1396.43
cytochrome c oxidase subunit 5B, mitochondrial-like	120	2.40232E-79	1368.23
PREDICTED: uncharacterized protein LOC106785155	262	2.246E-125	1356.79
ferritin subunit	219	5.875E-108	1355.36
CCHC-type zinc finger protein CG3800	154	6.71129E-88	1338.05
fructose-bisphosphate aldolase-like	365	0	1326.07
calcium-transporting ATPase sarcoplasmic/endoplasmic reticulum type isoform X2	1002	0	1306.83
calcium-transporting ATPase sarcoplasmic/endoplasmic reticulum type isoform X2	1002	0	1306.83
calcium-transporting ATPase sarcoplasmic/endoplasmic reticulum type isoform X2	1002	0	1306.83
PREDICTED: uncharacterized protein LOC107997824	118	4.77421E-29	1302.54
NA	134		1251.75
NA	110		1212.96
elongation of very long chain fatty acids protein 1-like	105	2.19376E-31	1198.59
probable Bax inhibitor 1	237	8.998E-150	1195.64
neuromodulin-like	123	1.2022E-40	1192.43
NA	100		1174.61
ATPase inhibitor mai-2, mitochondrial-like	104	2.84229E-53	1163.88
peptidyl-prolyl cis-trans isomerase-like	104	1.17655E-67	1121.5
polyadenylate-binding protein 1	630	0	1120.72
PREDICTED: uncharacterized protein LOC106790187	116	3.82666E-61	1117.26
NA	101		1094.29
GATA zinc finger domain-containing protein 14-like	115	3.78527E-32	1089.43
NA	110		1089.24
NA	110		1089.24
NA	110		1089.24
NA	99		1075.52
NA	99		1075.52
leucine-rich repeat-containing protein 15-like	631	0	1048.65
ecdysteroid-regulated 16 kDa protein	156	1.87234E-82	1044.27
chitinase-like protein Idgf4	434	0	1015.76
acyl-CoA Delta(11) desaturase-like	354	0	1015
myosin heavy chain, muscle isoform X9	1177	0	1009.84
NA	113		1002.76
hornerin-like isoform X1	123	2.34556E-31	999.512

Table S6. Annotation of top 100 highly expressed genes in the venom gland of *Polistes rothneyi*.

Putative venom protein/peptide	Length (aa)	E-value	TPM
protein AAR2 homolog	383	0	6436.25
protein lethal(2)essential for life-like	196	2.3236E-117	5446.11
RING finger protein 10	725	0	5120.23
cytochrome P450 4g15	364	0	4499.09
cytochrome P450 4g15	186	2.6801E-130	4283.4
uncharacterized protein LOC111674363	104	1.87695E-26	4110.57
polyubiquitin	111	3.30796E-74	4023.94
CCHC-type zinc finger protein CG3800	154	6.71129E-88	3920.57
endocuticle structural glycoprotein SgAbd-1-like	143	5.8166E-102	3779.37
NA	130		3672.33
protein lethal(2)essential for life-like	259	2.421E-112	3447.35
60S acidic ribosomal protein P2	113	8.41733E-36	3004.64
maternal effect protein oskar	408	0	2908.16
maternal effect protein oskar	408	0	2908.16
ATP synthase lipid-binding protein, mitochondrial	138	6.2839E-42	2889.68
peptidyl-prolyl cis-trans isomerase FKBP1A	109	1.61042E-76	2875.64
60S ribosomal protein L44 isoform X2	104	6.99126E-59	2737.42
neuromodulin-like	124	1.70975E-32	2734.53
acidic leucine-rich nuclear phosphoprotein 32 family member B-like	115	6.91458E-09	2694.33
phospholipase A1 1	149	5.19726E-59	2670.99
histone H4	103	3.0554E-52	2583.47
elongation factor 1-alpha-like	462	0	2521.73
NA	139		2473.92
profilin	126	1.24751E-89	2387.71
actin-5C	376	0	2123.81
serine/threonine-protein kinase 17B-like	339	3.4039E-141	2047.41
60S acidic ribosomal protein P1	113	4.26991E-44	1982.63
thioredoxin-2-like	108	3.86025E-71	1967.86
polyubiquitin-B	180	2.6767E-110	1954.76
heat shock protein 83	719	0	1906.72
histidine protein methyltransferase 1 homolog isoform X1	281	0	1868.44
heat shock protein 70 A2-like	640	0	1800.44
tubulin alpha-1 chain	450	0	1778.78
nuclease-sensitive element-binding protein 1 isoform X3	278	6.8825E-101	1620.37
proliferation-associated protein 2G4	107	1.62911E-61	1606.61
polyadenylate-binding protein 1	630	0	1544.8
NA	125		1525.37
cytochrome c-2	108	1.97421E-75	1520.72
nuclease-sensitive element-binding protein 1 isoform X2	257	8.8298E-101	1499.03
NA	186		1459.73
ubiquitin-conjugating enzyme E2-17 kDa	147	5.2792E-108	1444.65
phospholipase A1-like	247	6.4567E-165	1437.65
calcium-binding mitochondrial carrier protein SCaMC-2 isoform X1	99	2.21536E-44	1429.87
NA	117		1395.9
cytoplasmic polyadenylation element-binding protein 1 isoform X1	724	0	1379.05
NA	113		1376.8
NA	113		1376.8
putative ATP-dependent RNA helicase me31b isoform X1	444	0	1376.12
PREDICTED: uncharacterized protein LOC106793063	110	1.46813E-52	1352.74
phospholipase A1	237	3.3936E-154	1335.53
40S ribosomal protein S24	130	3.37939E-91	1320.79
uncharacterized protein LOC113219351	127	2.06939E-61	1311.63
tubulin beta-1 chain-like	447	0	1308.91
peroxiredoxin 1-like	193	1.7059E-140	1294.58
maternal protein exuperantia	432	0	1294.21
elongation factor 1-alpha	462	0	1293.16
hyaluronidase	321	0	1275.32
40S ribosomal protein S20	121	4.1396E-84	1268.25

PREDICTED: uncharacterized protein LOC107068002	128	3.15289E-79	1264.41
phospholipase A1-like	338	0	1245.99
dnaJ homolog subfamily A member 4	135	2.90697E-92	1245.55
dnaJ homolog subfamily A member 4	135	2.90697E-92	1245.55
high mobility group protein DSP1-like	200	4.1447E-116	1233.87
60S ribosomal protein L8	257	3.5675E-171	1225
60S ribosomal protein L8	176	2.4748E-108	1225
vitellogenin-like	1753	0	1201.48
histone H2A-like	124	3.23327E-73	1192.35
heat shock 70 kDa protein cognate 3 isoform X1	659	0	1168.33
coiled-coil-helix-coiled-coil-helix domain-containing protein 10, mitochondrial	151	3.30855E-45	1166.59
40S ribosomal protein S2	281	1.4491E-162	1154.64
multiple epidermal growth factor-like domains protein 10	156	3.48494E-82	1150.43
60S ribosomal protein L36	115	1.16071E-77	1147.82
death-associated protein 1	102	4.29303E-55	1131.13
death-associated protein 1	102	4.29303E-55	1131.13
4-coumarate--CoA ligase 1-like	540	0	1115.37
polyubiquitin-B	275	0	1108.97
small integral membrane protein 14	107	1.25171E-62	1108.7
NA	102		1067.6
NA	102		1066.52
60S ribosomal protein L22-like	139	2.6337E-75	1065.29
guanine nucleotide-binding protein G(I)/G(S)/G(T) subunit beta-1	340	0	1059.19
phospholipase A1-like	151	2.80069E-94	1058.56
PREDICTED: uncharacterized protein LOC107071063	112	4.51628E-46	1052.12
multiple epidermal growth factor-like domains protein 10	450	0	1041.45
nucleoplasmin-like protein isoform X1	180	7.53694E-86	1027.96
RNA-binding protein squid-like	288	3.1323E-162	1026.92
NA	124		1023.58
NA	101		1020.68
V-type proton ATPase 16 kDa proteolipid subunit	158	9.3708E-105	1015.34
eukaryotic translation initiation factor 5A	160	6.0794E-117	1009.54
40S ribosomal protein S10-like	159	2.3886E-101	1007.51
40S ribosomal protein S11	125	8.46688E-77	996.721
triosephosphate isomerase	247	7.595E-180	991.605
40S ribosomal protein S26	114	1.2361E-79	986.479
60S ribosomal protein L13a	204	3.6209E-148	986.124
KIF1-binding protein	609	0	984.809
aromatic-L-amino-acid decarboxylase isoform X2	480	0	973.187
NA	100		963.428
60S acidic ribosomal protein P0	317	0	948.082
venom serine protease precursor	276	8.819E-148	947.08

Table S7. Annotation of top 100 highly expressed genes in the venom gland of *Polistes snelleni*.

Putative venom protein/peptide	Length (aa)	E-value	TPM
NA	100		27570.9
60S ribosomal protein L44 isoform X2	104	6.99126E-59	4758.78
PREDICTED: uncharacterized protein LOC105556196	130	1.57216E-21	4751.47
protein lethal(2)essential for life-like	196	4.2543E-130	4315.87
NA	99		3838.97
acidic leucine-rich nuclear phosphoprotein 32 family member B-like	115	6.91458E-09	3823.34
histone H4	103	3.0554E-52	3791.88
uncharacterized protein LOC112694992	102	4.5849E-16	3473.16
heat shock 70 kDa protein cognate 4	651	0	3346.65
osteomodulin-like	108	1.98351E-29	3197.85
protein LSM14 homolog B isoform X2	484	0	2965.88
CCHC-type zinc finger protein CG3800	154	6.71129E-88	2746.42
myosin regulatory light chain sqh	174	3.32E-108	2534.73
PREDICTED: uncharacterized protein LOC106750966	105	2.93777E-47	2497.82
60S acidic ribosomal protein P2	113	1.40776E-35	2457.65
NA	103		2448.35
maternal effect protein oskar	405	0	2351.24
maternal effect protein oskar	405	0	2351.24
maternal effect protein oskar	405	0	2351.24
death-associated protein 1	102	4.29303E-55	2131.21
neuromodulin-like	125	8.09347E-31	2019.99
NA	116		1972.77
RNA-binding protein 1-like isoform X1	101	7.82736E-58	1890.25
NA	270		1877.5
histone H2A-like	135	4.05999E-66	1741.64
protein lethal(2)essential for life-like	269	1.1758E-103	1677.86
histone H2B-like	123	6.41587E-61	1654.78
cofilin/actin-depolymerizing factor homolog	148	1.2444E-106	1619.77
histone H2A-like	124	3.23327E-73	1610.83
NA	138		1582.04
60S acidic ribosomal protein P1	115	3.67313E-40	1497.99
NA	150		1492.14
NA	107		1475.97
something about silencing protein 10	464	0	1471.54
calmodulin-like	149	4.5592E-104	1427.86
serine-arginine protein 55 isoform X15	112	9.95766E-54	1398.12
NA	102		1390.67
cytochrome c-2	108	8.59688E-75	1372.89
heat shock protein 83	123	9.41377E-79	1369.18
histone H3.3-like isoform X1	136	6.60532E-95	1342.56
gamma-aminobutyric acid receptor-associated protein	117	3.16001E-82	1336.57
chaoptin-like	121	3.74509E-39	1326.87
nucleoplasmin-like protein isoform X1	180	1.51941E-85	1320.83
histone H4	103	3.0554E-52	1296.89
60S ribosomal protein L14	152	7.69376E-75	1284.48
profilin	126	1.24751E-89	1277.44
heterogeneous nuclear ribonucleoprotein K-like isoform X1	100	3.49929E-62	1273.93
eukaryotic translation initiation factor 5	448	0	1268.73
60S ribosomal protein L37	110	1.04509E-52	1264.17
NA	102		1251.16
NA	104		1239.18
NA	113		1236.89
nuclease-sensitive element-binding protein 1 isoform X4	257	1.20632E-95	1218.56
nuclease-sensitive element-binding protein 1 isoform X4	257	1.20632E-95	1218.56
40S ribosomal protein S9 isoform X2	137	2.53581E-94	1218.11
60S ribosomal protein L34-like	119	2.37791E-70	1217.68
NA	100		1198.83
14-3-3 protein zeta isoform X2	247	1.1652E-170	1196.85

NA	127		1169.05
ATPase inhibitor mai-2, mitochondrial-like	104	2.41622E-58	1148.33
ubiquitin-conjugating enzyme E2-17 kDa	147	5.2792E-108	1146.41
guanine nucleotide-binding protein subunit beta-like protein-like isoform X2	111	6.45701E-73	1142
protein CDV3 homolog	101	1.11836E-69	1137.59
NA	103		1133.44
actin-5C	376	0	1132.88
actin-5C	376	0	1132.88
putative fatty acyl-CoA reductase CG5065	205	1.8918E-111	1130.34
tubulin alpha-1 chain	450	0	1105.36
cytoplasmic polyadenylation element-binding protein 1 isoform X2	506	0	1103.52
NA	114		1103.52
NA	111		1096.02
eukaryotic initiation factor 4A-I	424	0	1089.43
uncharacterized protein LOC113219351	122	1.46801E-61	1087.41
heat shock protein 83	599	0	1070.21
putative ATP synthase subunit f, mitochondrial	107	4.87724E-71	1069.15
40S ribosomal protein S24	130	3.37939E-91	1065.57
tubulin beta-1 chain-like	447	0	1062.4
NA	99		1058.83
40S ribosomal protein S15Aa	130	2.12552E-92	1051.47
putative ATP-dependent RNA helicase me31b isoform X1	444	0	1048.03
60S ribosomal protein L36	115	1.16071E-77	1042.85
60S ribosomal protein L36	115	1.16071E-77	1042.85
PREDICTED: uncharacterized protein LOC106784792 isoform X4	122	1.02265E-53	1041.07
polyadenylate-binding protein-interacting protein 2 isoform X1	154	8.36777E-81	1039.94
NA	133		1030.98
DNA-directed RNA polymerase I subunit RPA1	1701	0	1029.07
DNA-directed RNA polymerase I subunit RPA1	1686	0	1029.07
NA	127		998.34
elongation factor 1-alpha-like	462	0	998.34
PREDICTED: uncharacterized protein LOC107071462 isoform X1	114	9.92706E-24	992.701
polyadenylate-binding protein 1	630	0	983.885
nuclease-sensitive element-binding protein 1 isoform X2	278	5.12495E-97	975.057
zinc finger CCCH domain-containing protein 11A-like	180	5.6233E-118	965.472
V-type proton ATPase 16 kDa proteolipid subunit	158	9.3708E-105	961.406
polyubiquitin-B	193	1.7E-114	953.371
cytoplasmic polyadenylation element-binding protein 1 isoform X3	686	0	925.739
upstream activation factor subunit spp27	261	6.56107E-65	891.394
ATP synthase-coupling factor 6, mitochondrial isoform X2	102	1.1481E-56	891.144
short-chain specific acyl-CoA dehydrogenase, mitochondrial	374	0	886.294
uncharacterized protein LOC105432314	110	1.6379E-15	873.66

Table S8. Annotation of top 100 highly expressed genes in the venom gland of *Eumenes decoratus*.

Putative venom protein/peptide	Length (aa)	E-value	TPM
NA	127		156798
phospholipase A2-like	184	6.17849E-18	55093.1
neprilysin-4	727	4.1271E-127	29479.9
neprilysin-1 isoform X2	161	8.29179E-13	22484.6
neprilysin-4	431	4.97005E-51	22484.6
chymotrypsin-2	257	6.88856E-86	22354.5
hyaluronidase	131	4.10134E-36	13836.2
NA	105		11934.3
NA	117		11257.4
heat shock 70 kDa protein cognate 3 isoform X1	663	0	9684.18
hyaluronidase	372	2.9394E-146	8510.5
bromodomain-containing protein 7 isoform X2	499	0	8022.82
protein disulfide-isomerase A3	492	0	6598.28
calreticulin	439	0	6324.36
zinc finger CCHC domain-containing protein 8 homolog	619	0	5569.94
NA	105		5569.94
small integral membrane protein 14	107	2.71472E-43	5569.94
NA	121		4993.46
tachykinins isoform X2	296	1.043E-127	4758.35
hyaluronidase	248	3.97446E-97	4627.23
NA	116		4404.85
neprilysin-11-like	100	6.60277E-12	4174.16
protein disulfide-isomerase	495	0	4038.13
60S acidic ribosomal protein P2	113	6.87784E-33	3451
uncharacterized protein LOC111694314	126	3.97218E-55	3107.4
uncharacterized protein LOC111694314	126	3.97218E-55	3107.4
NA	108		3021.46
NA	105		2956.33
membrane metallo-endopeptidase-like 1	261	8.56933E-28	2845.82
NA	114		2824.35
NA	116		2824.35
membrane metallo-endopeptidase-like 1	705	2.47594E-78	2769.52
endothelin-converting enzyme 1 isoform X2	266	1.14487E-23	2749.87
neprilysin-4	511	3.3985E-41	2733.02
60S ribosomal protein L9	113	4.84104E-78	2675.6
protein dimmed-like isoform X2	204	2.5615E-92	2551.17
DNA-directed RNA polymerase I subunit RPA1	555	0	2509.63
endothelin-converting enzyme 1-like	145	2.28021E-28	2497.57
neprilysin-4	711	2.19471E-74	2482.39
neuroligin 5 isoform X2	100	1.90663E-07	2473.33
transmembrane protein 14C	110	4.72221E-42	2400.84
NA	101		2342.63
NA	106		2342.63
NA	124		2342.63
NA	124		2342.63
NA	109		2342.63
NA	109		2342.63
NA	117		2244.04
NA	117		2244.04
NA	117		2244.04
NA	117		2244.04
NA	117		2244.04
endothelin-converting enzyme 1	276	7.66949E-42	2205.84
40S ribosomal protein S25	119	1.94623E-43	2126.65
neprilysin-4	735	2.5462E-84	2044.96
NA	136		1950.15
NA	136		1950.15
NA	136		1950.15

NA	136		1950.15
neprilysin-4	450	1.83907E-53	1947.92
NA	110		1846.22
alpha-amylase-like	489	0	1844.15
mesencephalic astrocyte-derived neurotrophic factor homolog	175	4.99E-103	1757.36
mesencephalic astrocyte-derived neurotrophic factor homolog	175	4.99E-103	1757.36
mesencephalic astrocyte-derived neurotrophic factor homolog	175	4.99E-103	1757.36
60S ribosomal protein L31	123	1.13224E-78	1722.53
von Hippel-Lindau-like protein	184	2.76941E-56	1712.92
40S ribosomal protein S24	130	1.78742E-75	1641.4
NA	105		1624.04
probable Bax inhibitor 1	237	2.6442E-124	1597.12
NA	99		1595.14
40S ribosomal protein SA	311	1.7763E-174	1556.49
death-associated protein 1	102	8.15391E-52	1547.97
neprilysin-4	415	4.34837E-58	1542.72
neprilysin-11-like	673	1.24782E-76	1542.7
60S ribosomal protein L36	115	3.02459E-76	1495.46
elongation factor 1-alpha	310	0	1462.86
40S ribosomal protein S17	130	3.29733E-92	1449.19
NA	146		1444.44
NA	119		1431.77
NA	119		1431.77
60S ribosomal protein L28	138	2.76087E-58	1430.19
neuromodulin-like	125	4.78893E-26	1424.61
neprilysin-4	523	8.71117E-37	1422.08
protein disulfide-isomerase A6	435	0	1411.18
NA	124		1403.25
40S ribosomal protein S3a	267	4.5317E-175	1396.8
40S ribosomal protein S10-like	176	2.7434E-103	1381.97
60S ribosomal protein L22-like	139	2.16366E-69	1357.27
40S ribosomal protein S19	154	1.4626E-109	1341.13
neprilysin-4	514	3.08126E-38	1318.2
heat shock 70 kDa protein cognate 4	651	0	1304.39
60S ribosomal protein L23	140	3.81261E-97	1292.79
ras-related protein Rab-2	214	1.2708E-142	1270.27
60S ribosomal protein L27a	147	3.14724E-84	1259.73
NA	103		1232.92
elongation factor 1-alpha-like	158	1.4291E-110	1210.05
elongation factor 1-alpha-like	158	1.4291E-110	1210.05
60S ribosomal protein L30	114	1.68095E-79	1209.45
polyubiquitin-like	106	3.41699E-69	1184.87

Table S9. Annotation of top 100 highly expressed genes in the venom gland of *Sphecidae* sp.

Putative venom protein/peptide	Length (aa)	E-value	TPM
ejaculatory bulb-specific protein 3-like	105	2.43611E-40	38623.6
PREDICTED: uncharacterized protein LOC106744880 isoform X1	103	4.93001E-13	25626.7
PREDICTED: uncharacterized protein LOC108576964	104	2.89939E-06	12293.1
NA	106		9508.08
PREDICTED: uncharacterized protein LOC107224416	115	7.76162E-18	8758.77
transmembrane and TPR repeat-containing protein CG4341-like	432	0	8481.03
eukaryotic translation initiation factor 4 gamma 2 isoform X1	867	0	6591.7
NA	255		6168
NA	190		5352.56
fibrous sheath CABYR-binding protein	462	9.00398E-09	5213.69
NA	111		5172.02
PREDICTED: uncharacterized protein LOC108576964	140	2.74545E-15	5137.12
endocuticle structural glycoprotein SgAbd-1-like	143	3.45001E-89	4846.7
NA	117		4375.28
PREDICTED: uncharacterized protein LOC108573181	138	5.22269E-71	4371.14
rap1 GTPase-activating protein 1 isoform X1	126	3.92449E-12	4250.01
PREDICTED: uncharacterized protein LOC108553831 isoform X1	390	3.06622E-98	3751.49
PREDICTED: uncharacterized protein LOC107186552	131	3.07513E-20	3751.49
PREDICTED: uncharacterized protein LOC100878814	287	3.56032E-72	3751.49
ATP synthase lipid-binding protein, mitochondrial	138	1.32986E-55	3662.05
chitinase-like protein EN03 isoform X2	434	0	3108.74
NA	111		3101.49
icarapin-like	231	3.57886E-71	2857.12
uncharacterized protein LOC105276036	233	1.34838E-49	2750.53
ADP/ATP translocase	300	0	2701.61
V-type proton ATPase subunit G	118	4.7656E-49	2673.97
thioredoxin-2	105	4.83586E-56	2661.08
U1 small nuclear ribonucleoprotein 70 kDa-like isoform X1	264	1.48391E-66	2471.08
neprilysin-11-like	725	0	2409.32
NA	99		2359.79
NA	120		2359.79
PREDICTED: uncharacterized protein LOC107998437	134	2.5225E-79	2359.79
NA	120		2359.79
NA	175		2252.88
NA	120		2252.88
NA	175		2252.88
NA	120		2219.95
NA	99		2219.95
NA	99		2219.95
NA	120		2219.95
PREDICTED: uncharacterized protein LOC107998437	134	2.5225E-79	2209.55
RING-type E3 ubiquitin-protein ligase PPIL2	521	0	2209.55
carbonic anhydrase 2 isoform X2	273	0	2182.06
uncharacterized protein LOC112455139	105	1.20122E-23	2174.22
uncharacterized protein LOC112455139	105	1.20122E-23	2174.22
uncharacterized protein LOC112455139	105	1.20122E-23	2174.22
actin-5, muscle-specific	376	0	2163.23
protein translation factor SUI1 homolog	110	1.11081E-78	2100.58
NA	100		1990.61
uncharacterized protein LOC105259235 isoform X3	104	8.00196E-50	1929.93
death-associated protein 1	102	3.0827E-56	1918.24
sodium/potassium-transporting ATPase subunit alpha-like	122	1.5971E-62	1883.62
NA	129		1868.09
ferritin-1 heavy chain-like	169	9.542E-102	1856.18
troponin C-like isoform X1	158	2.85462E-94	1849.43
60S ribosomal protein L44 isoform X2	104	6.99126E-59	1802.73
adenylosuccinate lyase isoform X1	485	0	1762.78
arginine kinase	374	0	1742.3

arginine kinase	374	0	1742.3
elongation factor 1-alpha	461	0	1710.8
PREDICTED: uncharacterized protein LOC106792090	870	0	1676.08
tropomyosin isoform X7	256	3.2402E-123	1673.86
heat shock 70 kDa protein cognate 4	651	0	1655.18
heat shock 70 kDa protein cognate 4	651	0	1655.18
PREDICTED: uncharacterized protein LOC107186552	131	3.07513E-20	1616.03
PREDICTED: uncharacterized protein LOC108553831 isoform X1	390	3.06622E-98	1616.03
cyclin-dependent kinase 2-like isoform X2	197	9.2424E-136	1607.55
myosin light chain alkali isoform X2	152	1.2952E-100	1607.55
PREDICTED: uncharacterized protein LOC100878241 isoform X2	262	2.5372E-149	1584.25
actin-5C	376	0	1511.69
V-type proton ATPase 16 kDa proteolipid subunit	108	1.69071E-52	1502.57
NA	115		1489.38
translationally-controlled tumor protein homolog	172	2.0329E-116	1488.15
retinoic acid receptor RXR-alpha-B isoform X1	427	0	1488.15
polyubiquitin-A isoform X3	340	0	1485
putative ATP synthase subunit f, mitochondrial	107	2.91427E-56	1447.32
60S acidic ribosomal protein P2 isoform X1	113	1.92376E-34	1400.17
chitinase-like protein Idgf4	127	8.6145E-77	1393.47
chitinase-like protein Idgf4	127	8.6145E-77	1393.47
chitinase-like protein Idgf4	127	8.6145E-77	1393.47
paramyosin, long form	876	0	1380.32
small integral membrane protein 14	104	1.55694E-33	1348.01
NA	110		1338.86
lysosomal aspartic protease	106	2.54104E-46	1324.53
fructose-bisphosphate aldolase isoform X2	365	0	1309.84
cytochrome b-c1 complex subunit 7	109	8.04268E-47	1273.98
NA	101		1235.26
polyubiquitin-B	170	4.6681E-118	1228.37
60S ribosomal protein L18	136	1.95494E-79	1221.69
uncharacterized protein LOC105829392, partial	118	5.56258E-20	1203.05
1-phosphatidylinositol 4,5-bisphosphate phosphodiesterase gamma-1	1021	0	1185.52
PREDICTED: uncharacterized protein LOC108570283	136	3.58796E-46	1179.63
ATPase inhibitor mai-2, mitochondrial isoform X1	103	3.61954E-31	1171.57
40S ribosomal protein S24-like isoform X2	132	1.24409E-79	1127.32
NA	104		1099.75
PREDICTED: uncharacterized protein LOC107997824	133	1.25068E-43	1087.64
alpha-crystallin B chain isoform X1	229	3.1588E-133	1084.07
actin	185	5.3788E-137	1076.72
NA	111		1072.24
otopetrin-3-like	107	4.42075E-36	1063.28

Table S10. Annotation of top 100 highly expressed genes in the venom gland of *Anterhynchium flavomarginatum*.

Putative venom protein/peptide	Length (aa)	E-value	TPM
NA	102		83726
ejaculatory bulb-specific protein 3	125	1.37E-66	71655.8
NA	103		60403
REDICTED: LOW QUALITY PROTEIN: uncharacterized protein LOC108577999	113	5.82E-07	42531.2
REDICTED: uncharacterized protein LOC108691125	112	1.78E-42	29237.4
circadian clock-controlled protein-like	104	1.39E-39	22754.8
circadian clock-controlled protein-like	250	1.75E-121	21481.7
NA	137		15361.2
NA	137		15361.2
ejaculatory bulb-specific protein 3-like	128	8.16E-63	12043.5
troponin C, isoform 1-like isoform X2	153	1.98E-102	9197.34
Arginine kinase	355	0	7674.27
REDICTED: uncharacterized protein LOC108691125	111	5.91E-62	7170.48
NA	137		6795.39
troponin I isoform X5	206	4.69E-95	6155.26
paramyosin, long form	877	0	5390.28
myosin light chain alkali isoform X2	152	1.01E-87	4188.18
DP/ATP translocase	300	0	4146.41
NA	286		3884.58
myosin heavy chain, muscle	1428	0	3870.17
roponin T isoform X8	287	1.81E-122	3383.11
uncharacterized abhydrolase domain-containing protein DDB_G0269086-like isoform X3	503	8.98E-26	3150.27
actin, clone 205-like isoform X1	294	0	2810.13
myosin type-2 heavy chain 2-like	133	1.87E-34	2727.44
ropomyosin-1	284	0	2673.92
myophilin	169	2.17E-118	2610.04
cathepsin L	113	3.66E-36	2482.56
cavenger receptor class B member 1	539	0	2416.73
putative beta-carotene-binding protein	242	8.24E-76	2393.76
NA	158		2161.15
REDICTED: uncharacterized protein LOC106790187	117	1.71E-46	2161
lpha-tocopherol transfer protein-like isoform X2	257	8.58E-155	2142.27
protein NPC2 homolog	154	1.89E-60	2138.37
leucine-rich repeat extensin-like protein 5	207	6.09E-78	2084.03
icarapin-like	227	3.58E-58	2080.75
polyubiquitin-B	232	2.77E-165	2063.9
NA	105		2019
ncharacterized protein LOC112588553	129	3.52E-40	1944.27
heat shock protein beta-1 isoform X2	189	6.35E-117	1918.84
NA	120		1879.48
ADH dehydrogenase subunit 4 (mitochondrion)	112	2.09E-17	1852.74
cytochrome c oxidase subunit 5B, mitochondrial-like	120	1.54E-71	1812.25
fructose-bisphosphate aldolase-like	365	0	1796.07
fructose-bisphosphate aldolase-like	365	0	1796.07
REDICTED: uncharacterized protein LOC106785313 isoform X2	120	1.99E-81	1722.14
REDICTED: uncharacterized protein LOC106785313 isoform X2	120	1.99E-81	1722.14
REDICTED: uncharacterized protein LOC106785313 isoform X2	120	1.99E-81	1722.14
REDICTED: uncharacterized protein LOC106785313 isoform X2	120	1.99E-81	1722.14
actin, clone 205-like isoform X1	294	0	1671.34
ATP synthase lipid-binding protein, mitochondrial	137	1.38E-39	1585.69
tropomyosin isoform X11	283	1.32E-145	1541.22
four and a half LIM domains protein 2 isoform X8	397	0	1501.53
NA	156		1466.24
iron-sulfur cluster assembly enzyme ISCU, mitochondrial isoform X2	166	2.20E-106	1372.9
REDICTED: uncharacterized protein LOC106787607	419	0	1340.55
REDICTED: uncharacterized protein LOC106787274	143	1.74E-18	1322.24
lyceraldehyde-3-phosphate dehydrogenase 1	333	0	1281.02

60S acidic ribosomal protein P1 isoform X1	113	3.38E-38	1250.25
NA	111		1205.95
gamma-aminobutyric acid receptor-associated protein	117	4.44E-82	1192.03
ATPase inhibitor mai-2, mitochondrial-like	104	2.32E-40	1189.36
cAMP-responsive element-binding protein-like 2	119	7.71E-59	1174.9
muscle LIM protein 1-like isoform X3	248	2.19E-108	1157.47
myosin regulatory light chain 2	214	4.88E-103	1157.45
myosin regulatory light chain 2	214	4.88E-103	1157.45
myosin regulatory light chain 2	214	4.88E-103	1157.45
myosin regulatory light chain 2	214	4.88E-103	1157.45
NA	130		1149.21
NA	107		1140.2
troponin I isoform X2	212	4.40E-88	1114.17
heat shock 70 kDa protein cognate 4	650	0	1112.72
ranslation elongation factor 2	857	0	1087.94
4-3-3 protein zeta isoform X1	247	1.36E-169	1066.49
glutamine synthetase 2 cytoplasmic	405	0	1057.76
calcium-transporting ATPase sarcoplasmic/endoplasmic reticulum type isoform X3	1002	0	1023.63
calcium-transporting ATPase sarcoplasmic/endoplasmic reticulum type isoform X3	1002	0	1023.63
REDICTED: uncharacterized protein LOC106791603 isoform X2	150	3.10E-83	1018.4
alpha-tocopherol transfer protein-like isoform X1	128	7.60E-30	1017.26
polyadenylate-binding protein 1	630	0	1001.44
filamin-A-like	364	0	966.368
glycerol-3-phosphate dehydrogenase, mitochondrial isoform X1	719	0	949.545
lpha-tocopherol transfer protein-like isoform X2	300	0	944.777
lpha-tocopherol transfer protein-like isoform X2	300	0	944.777
henoloxidase-activating factor 2 isoform X1	120	3.02E-26	944.135
translationally-controlled tumor protein homolog	172	8.20E-106	928.534
translationally-controlled tumor protein homolog	172	8.20E-106	928.534
elongation factor 1-alpha-like	462	0	922.267
uscle LIM protein Mlp84B isoform X2	493	0	912.828
probable Bax inhibitor 1	237	2.04E-136	908.641
activating transcription factor of chaperone	367	5.99E-159	908.023
NADH dehydrogenase [ubiquinone] 1 alpha subcomplex subunit 6	123	5.99E-66	904.946
troponin T, skeletal muscle isoform X5	356	1.90E-153	904.143
40S ribosomal protein S24	133	2.17E-69	902.085
cytochrome c oxidase subunit NDUFA4	143	2.00E-36	887.371
NADH-ubiquinone oxidoreductase 49 kDa subunit-like	468	0	886.52
muscle-specific protein 20	184	2.14E-131	880.847
ATP-dependent RNA helicase p62-like isoform X1	431	0	877.98
REDICTED: uncharacterized protein LOC106793063	193	5.50E-74	865.288
succinate dehydrogenase [ubiquinone] iron-sulfur subunit, mitochondrial-like	280	0	863.137
peroxisomal hydratase-dehydrogenase-epimerase-like	263	2.08E-121	857.585

Table S11. Annotation of top 100 highly expressed genes in the venom gland of *Sceliphron deforme*.

Putative venom protein/peptide	Length (aa)	E-value	TPM
neural/ectodermal development factor IMP-L2	299	3.0879E-99	52188.9
PREDICTED: uncharacterized protein LOC108729897	127	2.25273E-12	48220
PREDICTED: uncharacterized protein LOC108729897	127	2.25273E-12	48220
NA	170		48220
NA	124		48220
venom acid phosphatase Acph-1-like isoform X1	384	8.5473E-160	39252.3
NA	104		39144.5
NA	234		25249.4
endothelin-converting enzyme 1-like isoform X2	136	2.93187E-16	16846.1
NA	118		15881.2
NA	118		15881.2
NA	118		15881.2
NA	161		13676.1
probable serine/threonine-protein kinase DDB_G0282963	134	3.03299E-20	12757.8
MAGUK p55 subfamily member 6 isoform X1	602	0	9351.47
NA	192		9351.47
MAGUK p55 subfamily member 6 isoform X1	602	0	9351.47
venom acid phosphatase Acph-1-like isoform X1	197	1.74681E-63	8875.85
PREDICTED: uncharacterized protein LOC108555132 isoform X1	108	1.22989E-17	8703.35
C2 domain-containing protein 5-like isoform X5	441	0	8355.46
membrane metallo-endopeptidase-like 1	253	4.54588E-21	6167.2
neprilysin-4	392	2.30117E-45	5713.35
neprilysin-4	708	7.99094E-74	5333.2
neprilysin-4	138	4.69959E-13	5070.86
C2 domain-containing protein 5-like isoform X5	441	0	4930.89
C2 domain-containing protein 5	1053	0	4930.89
C2 domain-containing protein 5	1053	0	4930.89
histidine decarboxylase	206	2.339E-115	4492.63
CAPA peptides-like	362	1.87447E-05	4491.06
NA	105		4233.02
glucose dehydrogenase [FAD, quinone]-like	616	0	3783.38
NA	102		3773.29
neprilysin-like isoform X2	137	2.84624E-13	3349.66
membrane metallo-endopeptidase-like 1	187	7.59906E-29	3029.67
glucose dehydrogenase [FAD, quinone]	237	3.73384E-73	2817.55
NA	188		2630.57
NA	339		2630.57
NA	261		2630.57
NA	186		2630.57
NA	339		2630.57
NA	101		2630.57
death-associated protein 1	102	2.52883E-61	2502.01
PREDICTED: uncharacterized protein LOC108553804	301	3.1033E-113	2298.88
calreticulin	403	0	2068.53
NA	164		2040.35
NA	117		1902.61
neprilysin-4	707	2.40541E-77	1894.93
neprilysin-4	540	1.75637E-57	1738.36
neprilysin-2-like	122	7.6461E-07	1654.21
PREDICTED: uncharacterized protein LOC100881064	132	3.21775E-59	1586.51
neprilysin-like isoform X2	123	7.58499E-10	1550.89
protein disulfide-isomerase	504	0	1538.15
PREDICTED: uncharacterized protein LOC107187631	225	1.87858E-23	1538.15
membrane metallo-endopeptidase-like 1	168	6.95225E-20	1451.48
membrane metallo-endopeptidase-like 1	150	3.34105E-21	1435.2
neprilysin-4	572	6.89348E-50	1435.2
neprilysin-4 isoform X2	140	1.10265E-10	1394.74
PHD finger protein 20 isoform X2	1392	0	1387.61

NA	133		1361.48
endothelin-converting enzyme 1	131	6.45751E-08	1349.09
NA	152		1339.18
60S acidic ribosomal protein P2	113	3.67988E-37	1267.48
neprilysin-4	392	2.30117E-45	1265.5
neprilysin-4	203	1.16279E-32	1265.5
heat shock 70 kDa protein cognate 3	656	0	1247.21
heat shock 70 kDa protein cognate 3	656	0	1247.21
NA	114		1209.18
neprilysin-like isoform X1	132	5.70826E-20	1179.24
NA	130		1166.55
NA	186		1155
NA	186		1155
NA	339		1155
NA	101		1155
PREDICTED: uncharacterized protein LOC107185637	477	0	1155
NA	261		1155
PREDICTED: uncharacterized protein LOC107185637	477	0	1155
NA	261		1155
NA	339		1155
uncharacterized protein LOC105666132	118	1.90938E-28	1140.95
teasome subunit beta type-2	207	1.6514E-136	1136.26
translationally-controlled tumor protein homolog	173	3.0197E-103	1116.7
PREDICTED: uncharacterized protein LOC100865315	102	9.53299E-50	1114.2
endothelin-converting enzyme 1 isoform X2	188	1.25392E-14	1073.77
NA	158		1049.55
NA	196		1049.55
NA	158		1049.55
endothelin-converting enzyme 1 isoform X1	225	1.53327E-18	1025.81
40S ribosomal protein S20	121	1.45085E-82	1008.57
NA	113		1007.37
cAMP-responsive element-binding protein-like 2 isoform X2	119	6.5382E-59	983.368
endothelin-converting enzyme 1 isoform X1	247	2.03091E-26	957.137
endoplasmin	800	0	888.071
endothelin-converting enzyme 1	145	9.82295E-07	880.543
PREDICTED: uncharacterized protein LOC100881997	166	5.582E-113	871.591
neprilysin-like	541	1.57794E-52	871.005
40S ribosomal protein S3a-like	267	7.4299E-166	869.767
endothelin-converting enzyme 1	118	1.57129E-06	853.558
NA	339		844.701
uncharacterized protein LOC725241 isoform X3	863	0	844.701
uncharacterized protein LOC725241 isoform X3	863	0	844.701

Table S12. Annotation of top 100 highly expressed genes in the venom gland of *Bombus ardens*.

Putative venom protein/peptide	Length (aa)	E-value	TPM
ejaculatory bulb-specific protein 3	100	8.59474E-58	112009
60S ribosomal protein L44 isoform X2	104	6.99126E-59	7273.83
omega-conotoxin-like protein 1	100	1.37925E-39	7234.29
uncharacterized protein PF11_0213	295	7.6278E-162	6802.27
ejaculatory bulb-specific protein 3	116	8.2804E-80	6456.8
NA	195		5928.69
60S ribosomal protein L36	115	1.44569E-77	5129.55
60S acidic ribosomal protein P1	111	1.63922E-47	4955.26
protein lethal(2)essential for life	137	3.74392E-57	4587.67
hydroxymethylglutaryl-CoA synthase 1	453	0	4015.31
NA	105		4015.31
hydroxymethylglutaryl-CoA synthase 1	453	0	4015.31
isopentenyl-diphosphate Delta-isomerase 1	241	1.9222E-172	3834.15
uncharacterized protein LOC100745189	99	1.88266E-59	3494.51
uncharacterized protein LOC105681061 isoform X2	96	4.36007E-35	3375.59
protein translation factor SUI1 homolog	110	1.11081E-78	3340.62
acyl-CoA Delta(11) desaturase isoform X4	351	0	3298.59
coiled-coil-helix-coiled-coil-helix domain-containing protein 10	142	2.73484E-48	3156.71
polyubiquitin-B	163	2.2526E-113	3137.15
NA	103		3022.84
NA	120		2943.36
NA	99		2912.03
formin-like protein CG32138 isoform X4	716	0	2890.72
60S acidic ribosomal protein P2	114	9.53553E-39	2730.64
formin-like protein CG32138 isoform X4	716	0	2690.67
40S ribosomal protein S26 isoform X1	114	1.81523E-79	2690.3
ecdysteroid-regulated 16 kDa protein	156	5.66734E-83	2598.96
40S ribosomal protein S23-like	143	1.3693E-101	2550.75
40S ribosomal protein S17-like	131	1.15688E-93	2549.74
protein APCDD1-like	444	0	2515.02
40S ribosomal protein S25	119	2.7424E-50	2415.31
farnesol dehydrogenase	251	1.1068E-153	2360.59
thioredoxin-2	105	3.51002E-73	2326.23
profilin	126	8.04157E-90	2306.51
uncharacterized protein LOC100644161	123	6.4862E-26	2099.92
icarapin-like	135	1.01974E-74	2039.12
14-3-3 protein zeta-like isoform X1	156	2.47619E-97	2027.93
icarapin-like	230	4.2435E-134	2012.05
calmodulin-like	149	4.5592E-104	2007.58
SET and MYND domain-containing protein DDB_G0273589	183	7.10985E-70	1980.12
NA	248		1980.12
NA	248		1980.12
60S ribosomal protein L35	123	6.40636E-64	1959.54
uncharacterized protein LOC100749740	244	0	1872.15
NA	136		1858.79
NA	101		1825.65
uncharacterized protein LOC100644176 isoform X1	140	3.89853E-88	1799.96
uncharacterized protein LOC100644176 isoform X1	140	3.89853E-88	1799.96
embryonic polarity protein dorsal isoform X2	599	0	1796.69
embryonic polarity protein dorsal isoform X2	624	0	1796.69
apoptosis-resistant E3 ubiquitin protein ligase 1 isoform X1	951	0	1796.69
heterogeneous nuclear ribonucleoprotein A1 isoform X2	320	1.2252E-110	1755.6
Na(+)/H(+) exchange regulatory cofactor NHE-RF1	115	1.30675E-37	1743.41
40S ribosomal protein S15Aa-like isoform X1	127	1.22E-89	1738.69
40S ribosomal protein S12	141	2.3573E-100	1735.21
ATP synthase lipid-binding protein, mitochondrial	144	1.40492E-53	1719.03
NA	177		1719.01
uncharacterized protein LOC113219351	130	5.64173E-14	1660.21

60S ribosomal protein L30-like	114	3.03211E-80	1656.65
60S ribosomal protein L27	134	1.05114E-94	1655.22
NA	99		1608.05
NA	123		1575.57
ubiquitin-60S ribosomal protein L40-like isoform X1	128	5.68352E-91	1572.18
40S ribosomal protein S20-like	121	5.81772E-84	1569.79
trithorax group protein osa	116	7.29131E-54	1533.07
elongation factor 1-alpha	461	0	1523
elongation factor 1-alpha	461	0	1523
NA	134		1469.13
RNA-directed DNA polymerase from mobile element jockey-like	99	7.55869E-34	1447.64
60S ribosomal protein L14	149	6.61887E-92	1419.77
small integral membrane protein 14	106	3.15243E-63	1414.75
cGMP-dependent 3',5'-cyclic phosphodiesterase isoform X2	101	9.49078E-55	1393.84
40S ribosomal protein S10	159	3.95312E-83	1385.98
gamma-aminobutyric acid receptor-associated protein-like	117	3.8084E-82	1370.67
four and a half LIM domains protein 2 isoform X8	102	2.29244E-13	1359.97
NA	102		1353.49
death-associated protein 1	102	2.2461E-68	1353.49
40S ribosomal protein S24	132	3.922E-81	1350.84
14-3-3 protein zeta isoform X2	114	1.2771E-73	1323.18
NA	191		1317.33
40S ribosomal protein S9 isoform X2	138	1.20252E-94	1317.28
protein lethal(2)essential for life	173	4.6401E-126	1316.41
NA	100		1292.66
10 kDa heat shock protein, mitochondrial	104	1.10131E-69	1262.92
heat shock 70 kDa protein cognate 4	646	0	1228.82
NA	104		1214.83
40S ribosomal protein S8	208	1.0331E-132	1205.13
40S ribosomal protein S16	148	1.5437E-103	1204.82
60S ribosomal protein L31	148	1.01464E-68	1180.52
uncharacterized protein LOC100642642	169	1.778E-124	1176.12
defensin-1	158	5.09188E-91	1163.46
NA	103		1148.43
60S ribosomal protein L8	257	7.7442E-176	1123.52
serine-arginine protein 55 isoform X13	145	1.23313E-83	1104.5
NA	108		1079.25
NA	105		1000.55
polyubiquitin	119	1.40968E-52	987.363
40S ribosomal protein S15-like	147	1.76236E-92	984.901
NA	124		978.588
60S ribosomal protein L11 isoform X1	191	1.1922E-126	970.792

Table S13. Annotation of top 100 highly expressed genes in the venom gland of *Bombus consobrinus*.

Putative venom protein/peptide	Length (aa)	E-value	TPM
ejaculatory bulb-specific protein 3 isoform X2	181	7.27E-57	44088.4
elongation of very long chain fatty acids protein 6-like	109	1.78E-59	13702.2
60S acidic ribosomal protein P1	111	3.03E-52	11502.7
coiled-coil-helix-coiled-coil-helix domain-containing protein 10	142	3.35E-47	9201.64
ejaculatory bulb-specific protein 3 isoform X2	206	7.45E-85	8887.83
60S acidic ribosomal protein P2	114	9.54E-39	8555.36
40S ribosomal protein S17-like	131	1.16E-93	8086.35
uncharacterized protein LOC113219351	244	7.52E-92	7944.94
ecdysteroid-regulated 16 kDa protein	156	2.26E-83	7869.22
heat shock 70 kDa protein cognate 4	646	0	7683.65
uncharacterized protein LOC105680602	106	4.71E-73	6955.11
60S ribosomal protein L30-like	114	3.03E-80	6534.1
60S ribosomal protein L36	115	2.82E-78	6526.81
40S ribosomal protein S24	132	3.92E-81	5809.66
acyl-CoA Delta(11) desaturase isoform X4	163	3.66E-103	5688.97
acyl-CoA Delta(11) desaturase isoform X4	163	3.66E-103	5688.97
NA	105		5372.74
40S ribosomal protein S15Aa-like isoform X1	130	3.37E-92	4897.34
2-acylglycerol O-acyltransferase 1	348	0	4845.65
icarapin-like	265	5.85E-125	4623.14
40S ribosomal protein S20-like	121	5.82E-84	4535.15
40S ribosomal protein S26 isoform X1	114	1.82E-79	4491.54
protein translation factor SUI1 homolog	110	1.11E-78	4419.41
40S ribosomal protein S12	141	1.15E-99	4374.9
ATP synthase subunit g, mitochondrial	99	3.86E-63	4097.42
40S ribosomal protein S8	208	2.18E-132	3902.96
microsomal glutathione S-transferase 1	150	6.17E-100	3892.81
40S ribosomal protein S18-like	152	5.10E-94	3869.92
farnesol dehydrogenase	264	4.54E-153	3843.13
farnesol dehydrogenase	264	4.54E-153	3843.13
40S ribosomal protein S3-like	242	3.26E-178	3785.39
ATPase inhibitor mai-2, mitochondrial	103	2.01E-46	3766.32
40S ribosomal protein S13-like	151	1.83E-108	3624.08
40S ribosomal protein S11	155	2.83E-88	3616.81
ubiquitin-like	109	4.33E-74	3522.77
ubiquitin-40S ribosomal protein S27a	156	2.36E-83	3450.36
60S ribosomal protein L32	134	3.18E-95	3399.41
cytochrome b-c1 complex subunit 8	109	1.58E-52	3205.92
60S ribosomal protein L31	123	5.22E-71	3150.06
60S ribosomal protein L31	123	5.22E-71	3150.06
60S ribosomal protein L31	123	5.22E-71	3150.06
60S ribosomal protein L9 isoform X2	190	3.77E-139	3130.64
40S ribosomal protein S2	284	5.18E-166	3129.93
40S ribosomal protein S19	154	2.90E-95	3127.25
40S ribosomal protein S4 isoform X1	262	0	3118.61
40S ribosomal protein S3a-like	267	3.93E-172	2855.04
elongation factor 1-alpha	461	0	2852.75
NA	101		2777.73
60S ribosomal protein L17	185	1.74E-122	2733.3
60S ribosomal protein L35	123	5.25E-66	2706.99
60S ribosomal protein L26	121	9.13E-56	2631.13
60S ribosomal protein L35a	146	6.31E-82	2485.6
40S ribosomal protein S5-like isoform X1	216	2.80E-157	2454.9
glutathione S-transferase	201	8.84E-146	2449.13
enolase	107	7.90E-72	2325.18
gamma-aminobutyric acid receptor-associated protein-like	117	3.81E-82	2243.34
60S ribosomal protein L21	183	1.51E-111	2221.87
60S ribosomal protein L24	154	3.37E-62	2211.5

polyubiquitin-B	229	5.76E-164	2208.19
40S ribosomal protein SA	309	0	2174.23
CD63 antigen isoform X2	103	2.32E-53	2165.92
60S ribosomal protein L23a	241	2.78E-96	2153.22
40S ribosomal protein S16	148	1.54E-103	2131.83
apidaecins type 73 isoform X2	104	3.88E-14	2121.97
60S ribosomal protein L8	257	7.74E-176	2102.15
ubiquitin-60S ribosomal protein L40-like isoform X1	128	5.68E-91	2072.61
ubiquitin-60S ribosomal protein L40-like isoform X1	128	5.68E-91	2072.61
superoxide dismutase [Cu-Zn], chloroplastic	176	6.26E-118	2028.76
60S ribosomal protein L27	134	1.05E-94	1973.83
60S ribosomal protein L27	134	1.05E-94	1973.83
40S ribosomal protein S15-like	147	1.76E-92	1926.45
uncharacterized protein LOC100650183	453	0	1923.12
cytochrome b-c1 complex subunit 7	109	4.05E-63	1896.99
acetyl-CoA acetyltransferase, cytosolic	275	0	1894.62
nucleoside diphosphate kinase	154	1.20E-109	1892.4
60S ribosomal protein L10a	217	1.94E-144	1865.18
40S ribosomal protein S9-like	193	3.78E-137	1839.88
60S ribosomal protein L13a-like	204	1.03E-149	1829.83
aquaporin AQPAn.G isoform X2	109	4.46E-74	1813.83
uncharacterized protein LOC100742137 isoform X1	167	1.93E-123	1805.05
40S ribosomal protein S10	159	5.65E-96	1801.3
40S ribosomal protein S10	159	5.65E-96	1801.3
60S ribosomal protein L13	219	2.95E-152	1799.4
elongation of very long chain fatty acids protein 6	275	0	1774.65
ferritin-3, chloroplastic	225	1.73E-165	1750.22
reticulon-1-A isoform X6	117	8.14E-79	1712.28
nose resistant to fluoxetine protein 6	114	1.01E-36	1708.8
cytochrome c oxidase subunit 6A1, mitochondrial-like	112	4.76E-69	1707.98
glyceraldehyde-3-phosphate dehydrogenase 2	128	3.27E-71	1679.48
60S ribosomal protein L19-like	200	2.07E-124	1656.21
uncharacterized protein LOC100745189	132	1.70E-82	1638.94
hydroxymethylglutaryl-CoA synthase 1	453	0	1627.22
hydroxymethylglutaryl-CoA synthase 1	453	0	1627.22
hydroxymethylglutaryl-CoA synthase 1	453	0	1627.22
hydroxymethylglutaryl-CoA synthase 1	453	0	1627.22
60S ribosomal protein L4	434	0	1614.61
myelin P2 protein isoform X2	132	1.68E-92	1555.04
myelin P2 protein isoform X2	132	1.68E-92	1555.04
myelin P2 protein isoform X2	132	1.68E-92	1555.04
very-long-chain enoyl-CoA reductase	300	0	1550.35

Table S14. Annotation of top 100 highly expressed genes in the venom gland of *Bombus ussurensis*.

Putative venom protein/peptide	Length (aa)	E-value	TPM
uncharacterized protein LOC105681061 isoform X2	103	6.71623E-29	18373.6
farnesol dehydrogenase	123	6.12275E-65	11840.2
ejaculatory bulb-specific protein 3 isoform X1	125	1.17987E-82	9576.21
heat shock 70 kDa protein cognate 4	646	0	8631
glutathione S-transferase	111	8.41342E-69	7578.26
icarapin-like	241	3.6009E-126	6943.3
omega-conotoxin-like protein 1	100	2.02743E-38	6744.55
coiled-coil-helix-coiled-coil-helix domain-containing protein 10	142	2.82107E-46	6741.51
NA	104		6196.83
acyl-CoA Delta(11) desaturase isoform X4	163	2.1297E-118	5306.83
60S ribosomal protein L17	101	4.22728E-71	5114.04
farnesol dehydrogenase	123	1.11866E-72	4950.47
NA	101		4653.28
uncharacterized protein LOC105681061 isoform X2	129	5.17499E-34	4634.01
polyubiquitin	103	2.22011E-69	4626.54
elongation factor 1-alpha	461	0	4296.7
ecdysteroid-regulated 16 kDa protein	156	2.25633E-83	4132.73
protein lethal(2)essential for life	173	3.2588E-127	4080.28
NA	103		3606.31
NA	99		3361.47
acyl-CoA Delta(11) desaturase isoform X2	351	0	3236.66
icarapin-like	231	6.4083E-113	3118.95
2-acylglycerol O-acyltransferase 1-like	220	9.2831E-120	3019.46
elongation of very long chain fatty acids protein 6	288	0	2931.62
14-3-3 protein zeta isoform X1	106	7.126E-74	2917.18
40S ribosomal protein S17-like	131	1.15688E-93	2618.88
uncharacterized protein LOC100644161	123	8.86119E-28	2528.83
farnesyl pyrophosphate synthase-like isoform X1	106	1.11918E-66	2494.59
gamma-aminobutyric acid receptor-associated protein-like	117	3.8084E-82	2407.79
heat shock protein beta-1 isoform X2	189	7.1374E-124	2325.31
60S ribosomal protein L30-like	114	3.03211E-80	2313.36
60S ribosomal protein L36	115	2.81645E-78	2216.85
CD63 antigen isoform X1	234	5.986E-124	2196.9
SPARC	307	4.0824E-173	1997.69
transferrin	707	0	1908.42
40S ribosomal protein S26 isoform X1	114	1.81523E-79	1863.12
putative fatty acyl-CoA reductase CG5065	282	3.2473E-164	1862.4
translationally-controlled tumor protein homolog	172	6.9976E-111	1856.09
2-acylglycerol O-acyltransferase 1	348	0	1838.05
fructose-bisphosphate aldolase isoform X2	365	0	1746.69
fructose-bisphosphate aldolase isoform X2	365	0	1746.69
uncharacterized protein LOC100644176 isoform X1	140	3.85767E-73	1741.12
ADP,ATP carrier protein 2	300	0	1739.24
40S ribosomal protein S2	284	1.6719E-165	1690.02
60S ribosomal protein L9 isoform X2	190	3.7683E-139	1675.71
small integral membrane protein 14	106	2.37526E-62	1664.45
LOW QUALITY PROTEIN: uncharacterized protein LOC105667095	310	0	1653.37
superoxide dismutase [Cu-Zn]	151	1.7044E-106	1644.33
NA	120		1634.32
probable phospholipid hydroperoxide glutathione peroxidase	131	1.90071E-92	1564.06
LOW QUALITY PROTEIN: uncharacterized protein LOC110120277	120	6.66008E-49	1550.01
LOW QUALITY PROTEIN: uncharacterized protein LOC110120277	120	6.66008E-49	1550.01
uncharacterized protein LOC100742137 isoform X1	167	1.9265E-123	1536.92
protein translation factor SUI1 homolog	110	1.11081E-78	1524.43
icarapin-like	135	2.30378E-75	1522.5
peroxiredoxin 1	195	1.9306E-144	1522.23
neurofilament heavy polypeptide	474	1.12485E-86	1514.63
ABC transporter G family member 20 isoform X1	767	0	1503.88

actin-5C	376	0	1499.81
actin-5C	376	0	1499.81
cAMP-responsive element-binding protein-like 2 isoform X2	119	1.71162E-62	1494.78
tubulin beta-1 chain-like	447	0	1491.54
death-associated protein 1	102	1.21452E-68	1480.85
14-3-3 protein zeta isoform X1	247	0	1477.16
elongation of very long chain fatty acids protein 6	275	0	1476.24
elongation of very long chain fatty acids protein 6	275	0	1476.24
40S ribosomal protein S15Aa-like isoform X1	130	3.37052E-92	1454.84
NA	99		1454.7
heat shock protein 83	725	0	1433.89
cytochrome b-c1 complex subunit 8	108	6.20885E-40	1419.22
chitinase-like protein Idgf4 isoform X2	434	0	1416.7
40S ribosomal protein S8	208	1.0331E-132	1410.75
40S ribosomal protein S12	141	1.1452E-99	1410.1
V-type proton ATPase 16 kDa proteolipid subunit	159	2.777E-103	1345.93
40S ribosomal protein S23-like	143	1.3693E-101	1345.87
60S acidic ribosomal protein P1	109	7.34118E-50	1341.44
60S acidic ribosomal protein P1	109	7.34118E-50	1341.44
NA	106		1336.03
ATP synthase subunit g, mitochondrial	99	3.00139E-63	1316.38
profilin	126	8.04157E-90	1281.64
profilin	126	8.04157E-90	1281.64
uncharacterized protein LOC100741859 isoform X5	296	0	1273.27
40S ribosomal protein S3a-like	267	3.3733E-172	1272.52
14-3-3 protein zeta	148	2.50849E-97	1253.37
NA	105		1230.69
reticulon-1-A isoform X3	191	2.1813E-140	1219.88
40S ribosomal protein S13-like	151	1.8321E-108	1218.81
hydroxymethylglutaryl-CoA synthase 1	453	0	1211.72
hydroxymethylglutaryl-CoA synthase 1	453	0	1211.72
hydroxymethylglutaryl-CoA synthase 1	453	0	1211.72
NA	106		1191.71
ferritin subunit	156	6.9363E-112	1175.64
2-acylglycerol O-acyltransferase 1	348	0	1161.43
acidic phospholipase A2 PA4 isoform X2	230	7.7919E-165	1157.25
farnesol dehydrogenase	115	1.2892E-68	1154.19
cytochrome c	108	8.20212E-76	1131.15
fatty acyl-CoA reductase 1 isoform X1	511	0	1127.57
polyadenylate-binding protein 1	630	0	1121.62
uncharacterized protein LOC100647597	117	3.95836E-71	1120.78
uncharacterized protein LOC112212744	101	2.56176E-32	1109.41

Table S15. TPM values of reference housekeeping gene dimethyladenosine transferase in 14 Aculeate bee and wasp species.

Species	TPM value
Vespa analis	23.03
Vespa crabro	16.58
Vespa dybowskii	35.43
Vespa simillima	38.66
Parapolybia varia	22.75
Polistes rothneyi	27.29
Polistes snelleni	32.33
Eumenes decoratus	20.39
Sphecidae sp.	7.38
Anterhynchium flavomarginatum	36.27
Sceliphron deforme	32.26
Bombus ardens	14.78
Bombus consobrinus	18.23
Bombus ussurensis	57.24

Table S16. Primers used in quantitative real-time PCR.

Name	Sequence	Length (bp)	Tm (℃)	%GC
Arginine kinase-Prot-F	GTGGCAAAACTTGGCAGTCG	20	55	60.5
Arginine kinase-Prot-R	CAGTGCCGAGATTGGTCGAA	20	55	60.5
Defensin 1-Prot-F	TCGTCGTAGTGACAGTCAAC	20	50	58.4
Defensin 1-Prot-R	ACTCGACGTTGTCTATCAGC	20	50	58.4
Dipeptidyl peptidase 4-Prot-F	CTTCATTACGGAGAACCCGG	20	55	60.5
Dipeptidyl peptidase 4-Prot-R	GATCGTGAAGATCGACGAGC	20	55	60.5
Hyaluronidase-Prot-F	GTGGAGACCGATTTTCCGAC	20	55	60.5
Hyaluronidase-Prot-R	CGTAGCTTCCGCTTCGATCA	20	55	60.5
Icarapin-Prot-F	TGTCCTCGTCCTTCCTGGAT	20	55	60.5
Icarapin-Prot-R	AGGAGCTTGGCCTAAACGAG	20	55	60.5
Phospholipase A2-Prot-F	TCGAACACCAGGAGGTGGAT	20	55	60.5
Phospholipase A2-Prot-R	ATGATCGTGTTCCCTGCAGC	20	55	60.5
Serine protease inhibitor-Prot-F	GGTCGCCTCATCAAACAATG	20	50	58.4
Serine protease inhibitor-Prot-R	CGGAAGATTGAGAACCCTTC	20	50	58.4
Tachykinin-Prot-F	GCGGAACGTCGTACAACTTT	20	50	58.4
Tachykinin-Prot-R	CGCGAATTCCGAAATATCGG	20	50	58.4
Vitellogenin-Prot-F	GCGTTTGTTGTTGACACTGG	20	50	58.4
Vitellogenin-Prot-R	ACGTTGAGGTACGTTCATGC	20	50	58.4
Dimethyladenosine transferase-Prot-F	CCGACGATGCAGTCATCATG	20	55	60.5
Dimethyladenosine transferase-Prot-R	CTCCTTGGAGATGGTGAGTG	20	55	60.5
Arginine kinase-Psne-F	CGATGCTAAGACCTTCCTTG	20	50	58.4
Arginine kinase-Psne-R	GATGTCGTTAACGGCGGTTA	20	50	58.4
Hyaluronidase-Psne-F	CGATAACGGCAAATACGAGG	20	50	58.4
Hyaluronidase-Psne-R	AATCGATTACGCCGATGCCA	20	50	58.4
Icarapin-Psne-F	TCGATACCGTTCTCGTCCTT	20	50	58.4
Icarapin-Psne-R	CCTGATCTACACAAGGACCA	20	50	58.4
Phospholipase A2-Psne-F	TCGGCAAATGCCAAGCAATC	20	50	58.4
Phospholipase A2-Psne-R	AGAACTTGCCACGTTTCCAG	20	50	58.4
Serine protease inhibitor-Psne-F	CTCAAGATAGACCCTTCCAC	20	50	58.4
Serine protease inhibitor-Psne-R	CACCTTTGTATGGAAGCTCG	20	50	58.4
Tachykinin-Psne-F	CGTGCAATGGCAATGGGATT	20	50	58.4
Tachykinin-Psne-R	GACTTCTTTCCCCTCATACC	20	50	58.4
Vitellogenin-Psne-F	TGGGAATCCGGCAACAAAGT	20	50	58.4
Vitellogenin-Psne-R	CCAGACCAGTGTCAACAACA	20	50	58.4
Dimethyladenosine transferase-Psne-F	CATGGTCGTATGGTAGGACG	20	55	60.5
Dimethyladenosine transferase-Psne-R	GTTGTGACCATGCTTGTGCC	20	55	60.5
Arginine kinase-Edec-F	TGACCAGGGAAGTGTTCGAC	20	55	60.5
Arginine kinase-Edec-R	CAGGGGCGTAGATTCCAACA	20	55	60.5
Icarapin-Edec-F	GTTGATACGGTCGTGGTCCT	20	55	60.5
Icarapin-Edec-R	GTGTAGCTATCGGAGTCGTC	20	55	60.5
Serine protease inhibitor-Edec-F	CTGCTTTCCGTTCAACCTCT	20	50	58.4
Serine protease inhibitor-Edec-R	ATGAGGTCCTTGATACGGTG	20	50	58.4
Tachykinin-Edec-F	TGCACCGATGGGCTTTCAAG	20	55	60.5
Tachykinin-Edec-R	GTACGTTTGCTTACCCCTCG	20	55	60.5
Neprilysin-Edec-F	TGGCAAGCGAGATATCCCGT	20	55	60.5

Neprilysin-Edec-R	CAATGCCGTTGCTCGCTTCA	20	55	60.5
Dimethyladenosine transferase-Edec-F	GGCCCATGGTCATATGGTAG	20	55	60.5
Dimethyladenosine transferase-Edec-R	CCACGTTTGTGCCATCACTG	20	55	60.5
Defensin 1-Bard-F	CTTCTCTTTGTGGCTGTAGC	20	50	58.4
Defensin 1-Bard-R	AAGGTCACAGGTCACTCTTC	20	50	58.4
Dipeptidyl peptidase 4-Bard-F	GCGAACAGCTTCAATGCTAC	20	50	58.4
Dipeptidyl peptidase 4-Bard-R	CAAAAGGACCCAAGTGGTTC	20	50	58.4
Hyaluronidase-Bard-F	GGCATCCTACAGAATTCGAG	20	50	58.4
Hyaluronidase-Bard-R	ACCACCGTTTCTCGTCACTA	20	50	58.4
Icarapin-Bard-F	CACCGTCGTTGTTTTACCGT	20	50	58.4
Icarapin-Bard-R	CCTGTTCCAATTCCAGGTTG	20	50	58.4
Phospholipase A2-Bard-F	CTTCTCGTGACAAGGGAAAG	20	50	58.4
Phospholipase A2-Bard-R	GTCGTCGTACGATTTTGCCA	20	50	58.4
Serine protease inhibitor-Bard-F	GCTCGCGCTAATTTTTCTGG	20	50	58.4
Serine protease inhibitor-Bard-R	AAGCAAACCAGTTGCAGCAG	20	50	58.4
Tachykinin-Bard-F	TTGAAACGTGCGATCATGGG	20	50	58.4
Tachykinin-Bard-R	CTTGCCTCTCATGCCTTGAA	20	50	58.4
Vitellogenin-Bard-F	GCCAGCAGGATGAATCTAAC	20	50	58.4
Vitellogenin-Bard-R	GTTCTGCGTTCCTCGAAATC	20	50	58.4
Dimethyladenosine transferase-Bard-F	GCCGACGTTAGAAATGTTGG	20	50	58.4
Dimethyladenosine transferase-Bard-R	CAGTTCGTGGGAGAACAGTA	20	50	58.4

Table S16
